# Global Burden and Incidence Trends in Cancers Associated with Human Papillomavirus Infection: A Population-Based Systematic Study

**DOI:** 10.3390/pathogens14090880

**Published:** 2025-09-03

**Authors:** Xiaojun Meng, Bolin Yang, Hanlu Yin, Jibiao Chen, Wenjuan Ma, Zhuping Xu, Yuan Shen

**Affiliations:** The Affiliated Wuxi Center for Disease Control and Prevention of Nanjing Medical University, Wuxi 214023, China; mengxiaojun@njmu.edu.cn (X.M.);

**Keywords:** human papillomavirus, cancer, incidence, burden, global trend

## Abstract

Background: Human papillomavirus (HPV) is responsible for a substantial fraction of anogenital and head and neck cancers (HNC). HPV-related cancers cause a heavy burden globally, with disparities across different cancers. We aimed to present an up-to-date global view of the patterns and incidence trends among HPV-related cancers. Methods: We collected data on HPV-related cancers from the GLOBOCAN 2022 database and the Cancer Incidence in Five Continents plus Compendium. Age-standardized incidence and mortality rate (ASIR and ASMR) were calculated to estimate the cancer burden. Spearman’s correlation tests were used to evaluate the associations with the Human Development Index (HDI). Joinpoint regression was conducted to evaluate the incidence trends in ASIR. Results: In 2022, 1,505,394 HPV-related cancer cases and 755,303 deaths were newly estimated worldwide, corresponding to an overall ASIR and ASMR of 20.9 and 10.2 per 100,000 people, respectively. Africa had the highest ASIR and ASMR compared with Asia, accounting for the most new cases and deaths. The primary cause was cervical cancer (ASIR 14.1 per 100,000 people); however, HNC exhibited the largest number of cases (685,204 cases). The total rates of HPV-related cancers were 1.3 times higher for ASIR and nearly three times higher for ASMR in low-HDI countries than in very high-HDI countries. A decreasing trend was observed for the ASIR of cervical cancer in most studied countries, compared to the increasing trends in HNC in females and anal cancer in both sexes. Conclusions: The global burden and trends of HPV-related cancers vary significantly among different cancer types according to region and sex. Particularly, cervical, HNC, and anal cancers should attract global attention. However, specific cancer types contributing to the heaviest burden should be identified at the country level to adjust resource allocation and improve access to quality health services.

## 1. Introduction

Human papillomavirus (HPV) causes one of the most common sexually transmitted infections worldwide [1]. HPV infection is not only the predominant cause of cervical cancer but also plays a causal role in a varying proportion of other anogenital cancers, including vulvar, vaginal, penile, and anal cancers, as well as a specific subset of head and neck cancers (HNC), notably cancers at the base of the tongue, tonsils, and other oropharynx sites [1]. Almost 95% of cervical cancer cases are related to HPV infection [2]. In addition, it is estimated that 90% of anal cancers, 70% of vulvar and vaginal cancers, 60% of penile cancers, and 70% of oropharyngeal cancers are attributable to persistent and high-risk HPV infection [3]. HPV-associated cancers cause a heavy burden globally, with disparities across different cancer types.

Cervical cancer is the second-leading cause of death among women with specific cancers [4]. The mortality rate of cervical cancer has either decreased or stagnated in most world regions [5]. However, the mortality rate in Africa remains high and continues to rise [4]. As one of the most preventable cancers through vaccination, the World Health Organization (WHO) announced a global call for action to eliminate cervical cancer by achieving 90–70–90 targets in all countries by 2030 [6]. Although vulvar and vaginal cancers are relatively rare in females, estimated new cases attributable to HPV infection increased in 2018 compared to those in 2012 by 30.0% and 16.7%, respectively [7]. In South Korea, the 5-year survival rate for vaginal cancer is the lowest among all gynecological cancers [8]. In America, the incidence and number of deaths due to vulvar cancer each year have increased by 0.6% and 1.5%, respectively [9]. Although rare, vaginal and vulvar cancers are becoming increasingly serious threats to women’s health.

Penile cancer is usually neglected in males because of its low incidence. The incidence of penile cancer exhibits significant regional variations, with higher rates in developing countries (2.6–6.8 per 100,000 people) compared to Western developed countries (0.3 per 100,000 people) [10]. People living with HIV (PLHIV) have a higher risk of penile cancer than uninfected populations, and HIV infection may accelerate the progression of penile intraepithelial neoplasia, increasing the risk of death from penile cancer [11]. Anal HPV infection is common in both sexes, with variations in sex and sexual orientation. Higher anal HPV prevalence was noticed in PLHIV and men having sex with men (MSM) [12]. Increasing trends were observed in the incidence and mortality rate of anal cancer in America, with approximately 0.2% of men and women being diagnosed with anal cancer at some point during their lifetime [13].

HNC represents a large group of cancers, among which the oral cavity (including lips) is the most common, accounting for more than 40% of cases [14]. Dramatic variations were observed in the association between HPV and HNC across anatomical subsites and geographical regions. A certain proportion of these cancers are caused by HPV, particularly in the oropharynx and, to a lesser degree, in the oral cavity and larynx [15]. Although the incidence rate of alcohol- and tobacco-related HNC has decreased because of the reduction in smoking and drinking in developed countries, the number of HNCs due to HPV infection has increased by more than 225% in the past 50 years [16]. With the increasing incidence of these cancers, the burden of HNC related to HPV infection is becoming a significant health issue worldwide.

Over the past few decades, tremendous changes have occurred in the global economy, society, population, and disease risk factors, leading to significant variations in the incidence and mortality rates of different types of HPV-associated cancers. However, there is currently a lack of comprehensive and systematic analyses on the burden of these cancers as a cluster. We sought to estimate the global burden of HPV-associated cancers by describing the geographical disparities in incidence and mortality in 185 countries by 2022 and elucidating the incidence trends in 26 countries with eligible registered data.

## 2. Materials and Methods

### 2.1. Data Sources and Population

In this population-based study, we collected updated incidence and mortality data from the GLOBOCAN 2022 database [https://gco.iarc.who.int/en, accessed on 15 September 2024] to determine the global burden of HPV-associated cancers in 2022. GLOBOCAN 2022 estimates the latest worldwide incidence, prevalence, and mortality for 36 cancer types in 185 countries, disseminated as Cancer Today at the Global Cancer Observatory. Crude cancer incidence rates were computed by dividing the observed number of new cancer cases or cancer-related deaths by the corresponding number of individuals in the at-risk population. These crude rates were then standardized as age-standardized incidence or mortality rates (ASIR or ASMR). All cancer types were coded using the International Classification of Diseases, Tenth Edition (ICD-10). Cancers of the lip, oral cavity (C00–C06), oropharynx (C09–C10), and larynx (C32) were grouped as HNCs. By contrast, cancers of the anus (C21), vulva (C51), vagina (C52), cervix uteri (C53), and penis (C60) were considered anogenital cancers.

Data for the incidence trends of HPV-associated cancers in available countries from 1978 to 2017 were obtained from the Cancer Incidence in Five Continents (CI5) plus. The CI5 series database includes data from cancer registries worldwide, covering a wide range of geographical locations, and specifically updates the data published in CI5 volumes, offering the latest incidence rates for selected populations. The CI5 plus database encompasses a set of comparable high-quality data on the annual incidence rates of specific cancers for 135 selected populations from 111 cancer registries, spanning the longest available timeframe up to 2017, covering all cancers and 28 significant types. The original database contains cancer cases classified by registry, sex, cancer site, 5-year age group (from 0–4, 5–9, continuing in that order to 85+ years), and person-years at risk, which can be used to calculate the ASIR. To ensure representativeness, countries were eligible for HPV-associated cancer incidence trend analysis where cancer registries covered at least 2 million inhabitants. Finally, 26 countries were included in this study.

### 2.2. Statistical Analysis

The ASIR was calculated as per 100,000 population based on the World Standard Population first proposed by Segi (1960), using the following formula [17], where *d_i_* is the number of cases (deaths) in age group *i*, *y_i_* is the number of person-years at risk in age group *i*, and *w_i_* is the number of individuals in (or weight of) age group *i* in the world standard population:

(1) $$\mathrm{ASIR}\;(\mathrm{ASMR})\;=\;\sum_{i}d_{i}w_{i}/y_{i}$$

We used locally weighted regression to plot smoothed fitting curves to present the incidence trends in selected countries according to sex and cancer site. Joinpoint regression was conducted to evaluate the HPV-associated cancer incidence trends by dividing the long-term ASIR trend line into several segments at special joinpoints, each described by a continuous linear function, using the joinpoint regression program (version 5.2.0). The annual percent change (APC) and average annual percent change (AAPC) with their corresponding 95% CIs were calculated using the joinpoint regression model to evaluate the long-term and recent period trends in HPV-associated cancer incidence in selected countries. Long-term trend refers to the longest period of HPV-associated cancer incidence data retrieved from the CI5 Plus database from 1978 to 2017. The latest 15-year period (2003–2017) was designated as the short-term trend. Spearman’s correlation tests were used to evaluate the associations between the ASIR or ASMR and the Human Development Index (HDI). All data analyses and plots were conducted using R software (version 4.3.3).

## 3. Results

**Figure 1:**
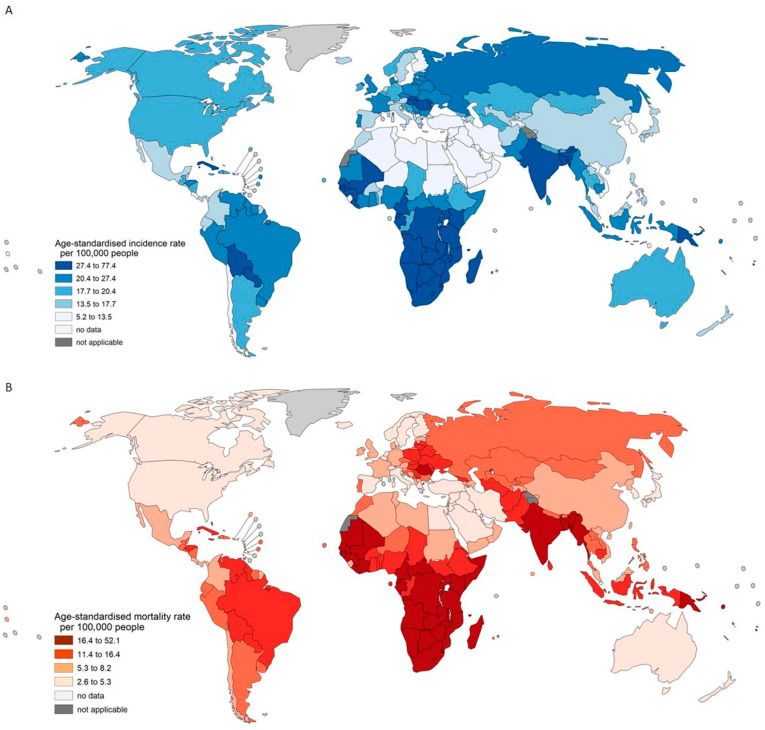
Estimated world age-standardized incidence rates and mortality rates of HPV-related cancers for males and females in 2022. Panel (**A**). Age-standardized incidence rates. Panel (**B**). Mortality rates. HPV—human papillomavirus.

According to the estimation from the GLOBOCAN 2022, a total of 1,505,394 HPV-associated cancer cases were newly diagnosed worldwide (582,666 males and 922,728 females), representing 7.5% of all cancer cases in 2022 and having a global ASIR of 20.9 (12.5 in males and 19.0 in females) per 100,000 people (Table S1). Overall, ASIR estimates of all HPV-associated cancers were highest in Africa (25.1 per 100,000 people) and Oceania (23.5) and lowest in Asia (20.0), accounting for the highest proportion of all cases at 56.9%, and in Northern America (20.1) (Figure 1). For cancer subtypes, cervical cancer contributed to 44.0% (662,044 cases) of HPV-associated cancers in 2022 with the highest ASIR of 14.1 per 100,000 people; HNC contributed mostly to 45.5% (685,204 cases, ASIR 7.0 per 100,000 people). For HNC, the ASIR in males was 11.2 per 100,000 people, approximately four times that of 3.1 per 100,000 people in females. At the country level, the total ASIR was the highest in Eswatini (77.4) and Zimbabwe (60.4), and the lowest in Saudi Arabia (5.2) and Kuwait (5.9).

In Africa, the high incidence burden of HPV-associated cancers was mainly driven by cervical cancer, with the highest ASIR of 26.4 per 100,000 people, far higher than other cancers (˂3.5; Table S1). Among the top 20 countries with the highest ASIR for cervical cancer worldwide, 17 (85.5%) were located in Africa, ranging from 39.1 per 100,000 in Botswana to 95.9 per 100,000 people in Eswatini. Similar to Africa, Latin America and the Caribbean, Asia, and Europe shared the same cancer type, with the highest ASIR of 15.1, 13.9, and 10.6 per 100,000 people, respectively. However, HNC had the highest ASIR in Oceania (10.5 per 100,000 people) and North America (9.0 per 100,000 people). Although Asia had the lowest total ASIR of HPV-associated cancers, India had the highest proportion—23.5% (354,044)—of all HPV-associated cancer cases worldwide in 2022, along with China, accommodating the largest proportion of 22.8% (150,659) of all cervical cancer cases.

Globally, 755,303 people died from HPV-associated cancers in 2022 (288,540 males and 466,763 females), representing 7.8% of all cancer deaths in 2022 and corresponding to a global ASMR of 10.2 (6.1 in males and 9.2 in females) per 100,000 people (Table S2). Overall, Africa suffered the highest ASMR of 16.5 per 100,000 people (Figure 1), whereas Asia contributed the most to the total deaths, amounting to a proportion of 60.9% (Table S2). Most deaths are caused by cervical cancer, accounting for 46.2% of all deaths worldwide, followed by HNC for 45.6%. At the country level, India (27.5%) and China (14.3%) had the most deaths caused by cervical cancer, owing to their high incidence rates. Cervical cancer has the highest ASMR (7.1 per 100,000 people) worldwide. More deaths were due to HNC than cervical cancer in North America, Europe, Oceania, and Asia. The highest ASMR for HNC was estimated for Bangladesh (10.3), Papua New Guinea (8.3), and India (8.2).

**Figure 2:**
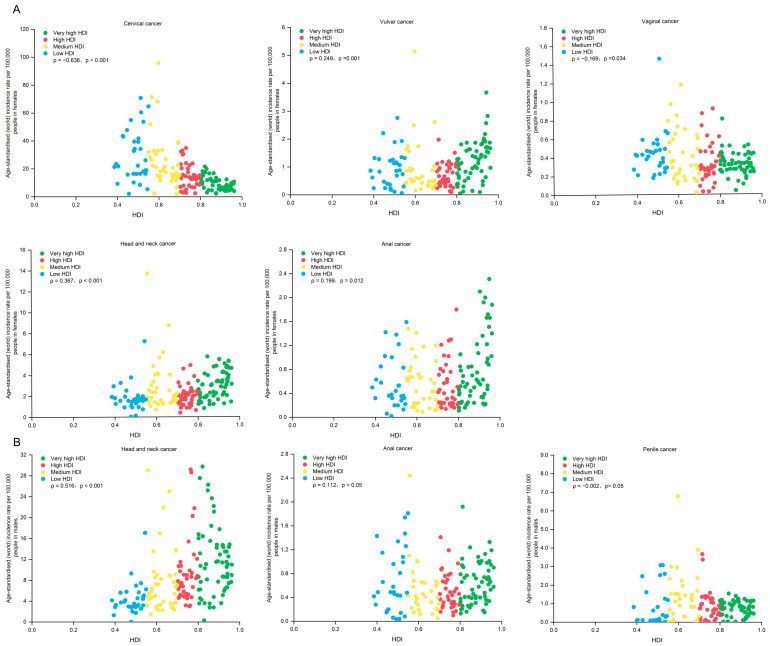
Age-standardized incidence rates of HPV-related cancers in females and males by site and HDI in 2022. Panel (**A**). Age-standardized incidence rates in females. Panel (**B**). Age-standardized incidence rates in males. HPV—human papillomavirus. HDI—Human Development Index.

**Figure 3:**
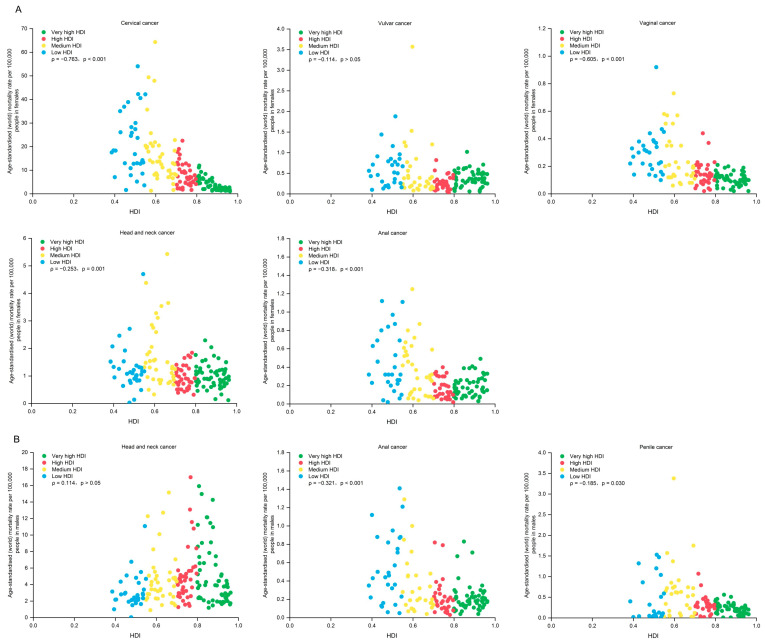
Age-standardized mortality rates of HPV-related cancers in females (**A**) and males (**B**) by site and HDI in 2022. Panel (**A**). Age-standardized mortality rates in females. Panel (**B**). Age-standardized mortality rates in males. HPV—human papillomavirus. HDI—Human Development Index.

The total ASIR of HPV-associated cancers was 1.3 times higher in countries with low HDI than countries with very high HDI (25.2 vs. 19.1 per 100,000 people; ρ = −0.308, *p* ˂ 0.001), whereas the total ASMR was nearly three times higher in low-HDI countries versus very high-HDI countries (17.1 vs. 6.4 per 100,000 people; ρ = −0.692, *p* ˂ 0.001). There were differences in the correlations between ASIR, ASMR, and HDI levels by subtype (Figure 2 and Figure 3, Table S3). In females, significant positive associations are observed between ASIR and HDI in vulvar cancer (ρ = 0.249, *p* = 0.001), anal cancer (ρ = 0.199, *p* = 0.012), and HNC (ρ = 0.367, *p* ˂ 0.001), as well as negative correlations in cervical cancer (ρ = −0.636, *p* ˂ 0.001) and vaginal cancer (ρ = −0.169, *p* = 0.034). In males, there are significant positive relationships between ASIR and HDI in HNC (ρ = 0.516, *p* ˂ 0.001). There was a negative correlation between HDI and ASMR in females for cervical cancer (ρ = −0.763, *p* ˂ 0.001), vaginal cancer (ρ = −0.605, *p* ˂ 0.001), and HNC (ρ = −0.253, *p* = 0.001).

**Figure 4:**
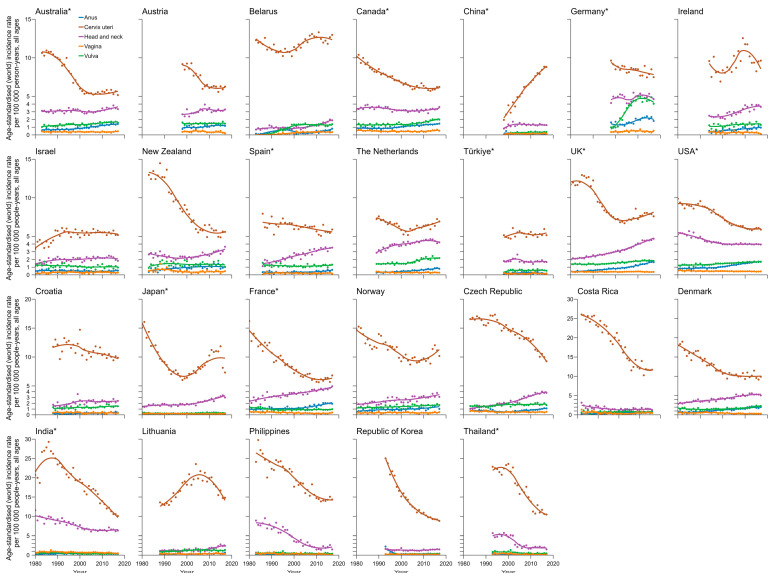
Trends in age-standardized incidence rates of HPV-related cancers in females by country and site (1978–2017). HPV—human papillomavirus. * Regional data.

**Figure 5:**
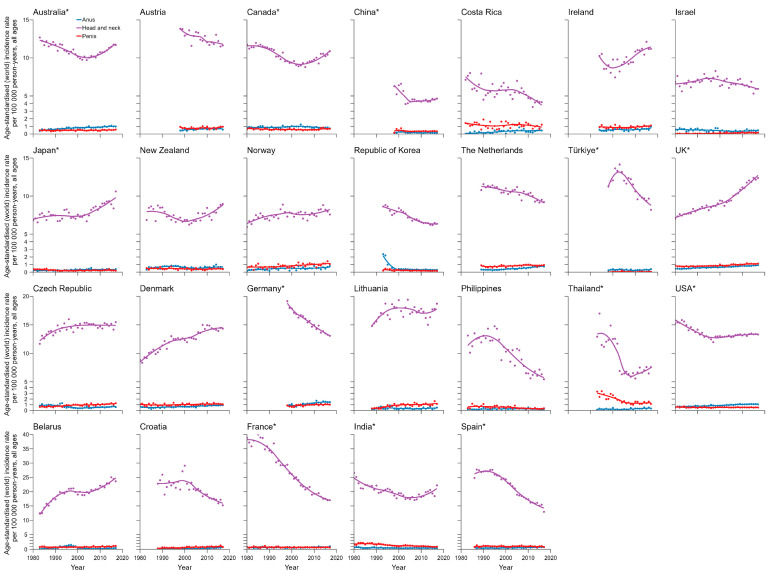
Trends in age-standardized incidence rates of HPV-related cancers in males by country and site (1978–2017). HPV—human papillomavirus. * Regional data.

**Figure 6:**
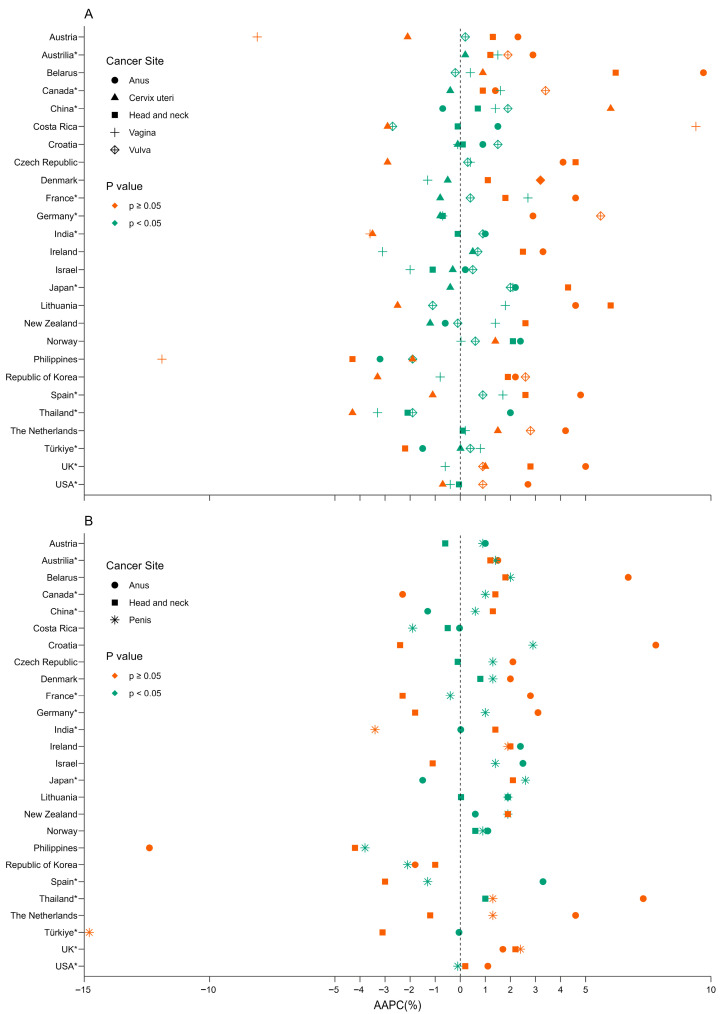
Average annual percent change in age-standardized incidence rates of HPV-related cancers in females (**A**) and males (**B**) by country and site (2003–2017). Panel **(A).** Average annual percent change in females. Panel (**B**). Average annual percent change in males. HPV—human papillomavirus. The orange shapes mean statistically significant (*p* < 0.05); green ones mean no statistical significance (*p* ≥ 0.05). * Regional data.

Long-term incidence trends of HPV-associated subtype cancers are shown in Figure 4 for females, Figure 5 for males, and Table S4 by population. A decreasing short-term trend was observed for the ASIR of cervical cancer in females in most countries in the 15 years before 2017. However, only the ASIR of cervical cancer in China showed an alarming rapid upward trend, with the highest AAPC of 6.0% (95% CI, 5.2–7.0%), followed by the Netherlands at 1.5% (95% CI, 0.8–2.1%) and Norway at 1.4% (95% CI, 0.1–2.7%). In comparison, the ASIR in Thailand decreased the most, with an AAPC of −4.3% (95% CI, −5.4% to −3.3%). Most countries presented an increasing trend of HNC incidence in females, with the highest AAPC in Belarus of 6.2% (95% CI, 4.7–8.0%). UK had the highest increase in ASIR of HNC and penile cancer in males, with an AAPC of 2.2% (95% CI, 2.0–2.4%) and 2.4% (95% CI, 1.2–3.5%), respectively. The ASIR of anal cancer increased in most countries, with the highest AAPC in females in Belarus of 9.7% (95% CI, 7.1–13.5%) and in males in Croatia of 7.8% (95% CI, 4.2–12.7%). The ASIRs of vulvar cancer and vaginal cancer displayed a relatively stable trend in females in most countries, with the largest growth in AAPC of 5.6% (95% CI, 4.0–7.5%) in Germany and AAPC of 9.4% (95% CI, 3.4–20.4%) in Costa Rica (Figure 6).

## 4. Discuss

To the best of our knowledge, this is the first study to use high-quality population-based data to provide an up-to-date global view of the patterns and incidence trends of HPV-associated cancers. In 2022, the incidence cases and deaths of HPV-associated cancers accounted for 7.5% and 7.8% of all cancers worldwide. The results showed that Africa has the heaviest burden of HPV-associated cancers because it has the highest ASIR and ASMR. However, India and China were jointly responsible for over 50% of estimated new cases and 40% of deaths, owing to their large national populations. The overall burden was primarily caused by cervical cancer, which had the highest ASIR in 148 countries and the highest ASMR in 149 countries. The HNC group had the highest number of cancer cases. The higher ASIR and ASMR of HPV-associated cancers had significant correlations with the increasing HDI, which indicated that these identified disparities suggested uneven allocations of socioeconomic resources, diverse degrees of risk factor control, and unequal access to healthcare services across countries.

Our study revealed that the total number of HNC cases exceeded that of cervical cancer and that HNC is the predominant HPV-associated cancer type in North America and Oceania. Tobacco and alcohol consumption are widely acknowledged as the main contributors to HNC worldwide [12]. However, there has been a consistent increase in the incidence of HPV-positive HNC over the past decade [18], coinciding with a decline in tobacco use, which is particularly evident in the rise of HPV-positive oropharyngeal cancers among young men in Northern Europe and North America [19]. In this study, the ASIR of HNC in males was approximately four times that in females, and an increasing trend of this cancer incidence in females was observed in most countries. The global burden of HNC poses a significant health challenge, requiring comprehensive and coordinated efforts. A systematic review and meta-analysis provided evidence for the potential efficacy of HPV vaccines in preventing oral infections [20]. Consequently, it should have the potential benefit of preventing HNC with an increasing number of countries introducing HPV vaccines to their national schedules for both sexes [21].

The incidence of cervical cancer has decreased worldwide and the highest ASIRs mainly occur in low- and middle-income countries (LMICs), especially in Africa, following the results of another global analysis [22]. However, the incidence rate in the vast majority of countries exceeds the threshold of 4 cases per 100,000 people set by the WHO initiative on cervical cancer elimination. Several countries have observed an increasing trend in the incidence of cervical cancer, particularly in China; among the 26 studied countries, China is experiencing a rapidly increasing trend. This increasing trend in China may be attributed to the period effects driven by the implementation of the National Cervical Cancer Screening Program in Rural Areas (NCCSPRA) in 2009 [23]. Another plausible explanation could be the cumulative exposure to risk factors during industrialization and urbanization, coupled with the enhancement of health service capabilities and increasing demand for cancer treatment [24]. These findings indicate that progress in reducing the cervical cancer burden has been heavily imbalanced across countries and regions over the past few decades. Assuming that rapid screening and vaccination coverage scale-ups are absent, particularly in LMICs, the process of eliminating cervical cancer is likely to be delayed, especially in Africa, which has the highest burden [25].

We found that penile cancer and anal cancer together accounted for 6% of all HPV-associated cancer cases worldwide, and the ASIR increased in some studied countries. Although they are relatively rare in the general population, PLHIV and MSM are disproportionately affected by these two types of cancers, especially HIV-positive MSM [11]. It has been proven that the HPV vaccine is effective against HPV infection and disease in males [26], but only 7.0% of males have received the first dose of HPV vaccine [13]. The incidence of anal cancer among men is predominantly observed within the MSM community, who are less prone to reap the benefits of herd immunity triggered by HPV vaccination programs primarily aimed at females [27]. In a context where unequal HPV vaccination access for males cannot be changed in the short term, it is even more important to explore other feasible measures for preventing anal cancer in males. The International Anal Neoplasia Society recommends anal cancer screening for high-risk groups. In their guidelines, MSM with HIV are recommended to initiate anal cancer screening at 35 years, as well as other PLHIV and MSM without HIV at age 45 years [28]. Because they are sexually transmitted diseases, practicing safe sexual practices, including heterosexual and homosexual behaviors, and reducing the number of sexual partners can also lower the chances of contracting HPV and HIV to prevent referring cancers.

More anogenital cancers and HNC are being found to be associated with HPV infection, suggesting that HPV is playing an increasingly significant role in the development of these cancers in both sexes [18]. Improving the awareness of HPV and access to vaccination are necessary to decrease HPV infection, associated cancers, and HPV-associated morbidity and mortality worldwide [1]. Prophylactic HPV vaccines are the most effective intervention for preventing HPV infections and related diseases. Some countries have gradually allowed males to receive the HPV vaccine; however, equal access to HPV vaccination for males remains unrealistic in many countries [29].Owing to limitations in vaccine production capacity, financial support, and vaccination strategies, even among women, HPV vaccination coverage remains low in some regions, especially in LMICs. Therefore, an integrated strategy should include reducing risky behaviors for acquiring HPV infection, recommending screening targeted risk populations, and improving access to quality treatment services for patients [30]. This study showed that the total ASIR and ASMR of HPV-associated cancers were much higher in low-HDI countries than in very high-HDI countries, which is consistent with other cancers [31]. However, a lower burden of cervical and vaginal cancers was found in countries with higher HDI levels, whereas a higher burden of vulvar cancer, anal cancer, and HNC was positively associated with higher HDI levels. Generally, countries with higher HDI tend to have better medical infrastructures, access to healthcare services, and cancer prevention programs, which may contribute to lower cancer incidence and mortality rates. However, some factors may be more prevalent in countries with higher HDI, such as obesity, smoking, alcohol consumption, and multiple sexual partners [32], which may increase the risk of developing HPV-associated cancers. Regardless, it is universally applicable that improving economic conditions and societal development to create a health-supporting environment is beneficial for reducing HPV-associated cancer risk. Therefore, governments and international organizations should prioritize resources in healthcare and HPV-associated cancer control initiatives in countries with lower HDI and continuously promote access to cancer prevention, screening, and treatment services for all individuals, regardless of their socioeconomic status or geographical location.

This study had some limitations. First, while the GLOBOCAN database provides valuable insights into global cancer statistics, it has certain weaknesses that should be acknowledged and considered because of various biases, including underreporting, misclassification, and selection. Noticeably, there is potential room to improve the data availability and quality across LMICs owing to their incomplete or inaccurate registration systems. Second, although only countries with cancer registries covering at least 2 million inhabitants were eligible for incidence trends analysis, the representativeness is still insufficient for entire countries with large populations such as India and China. Especially within the same country, owing to racial and cultural differences, there are also significant variations in incidence rates [33]. Third, advancements in HPV-associated cancer detection and diagnostic technologies, coupled with the refinement of monitoring systems, may lead to a passive increase in identified cancer cases, resulting in an overestimation of the upward trend in cancers.

## 5. Conclusions

HPV-associated cancers pose a significant global burden, with variations in cancer subtypes affecting different countries. The burden of cervical cancer remained the highest, while the burden of HNC and anal cancer showed an upward trend. All countries still need to invest more resources to continue expanding the coverage of HPV vaccine programs through promoting “gender-neutral” HPV immunization in both sexes, along with recommending screening for cervical and anal cancers among at-risk populations. Effectively mobilizing, coordinating, and allocating resources to facilitate the equitable distribution of health resources across different HDI regions, especially in LMICs, is crucial for the globally synchronized prevention of HPV-associated cancers.

## Data Availability

All data in the study are available at CI5 (https://ci5.iarc.who.int/previous/download, accessed on 15 September 2024) and GLOBOCAN (https://gco.iarc.fr/today/, accessed on 15 September 2024).

## Supplementary Materials

The following supporting information can be downloaded at: https://www.mdpi.com/article/10.3390/pathogens14090880/s1, Table S1: Estimated new cases and age standardized incidence rates (ASIR) for HPV related cancers by site and sex in 2022; Table S2: Estimated new cases and age standardized mortality rates (ASMR) for HPV rel ated cancers by site and sex in 2022; Table S3: Definitions of HDI regions; Table S4: International variation s in average annual percentage change (AAPC) of HPV related cancer incidence rates by site and sex.

## Abbreviations

The following abbreviations are used in this manuscript:

AAPC	average annual percent change
APC	annual percent change
ASIR	age-standardized incidence
ASMR	age-standardized mortality rate
CI5	Cancer Incidence in Five Continents
HDI	Human Development Index
HNC	head and neck cancer
HPV	human papillomavirus
ICD-10	International Classification of Diseases, Tenth Edition
LMICs	low-and middle-income countries
MSM	men having sex with men
NCCSPRAf	National Cervical Cancer Screening Program in Rural Areas
PLHIV	people living with HIV
WHO	World Health Organization
