# Supplementary material for: Global Burden and Incidence Trends in Cancers Associated with Human Papillomavirus Infection: A Population-Based Systematic Study
Source: Pathogens. 2025 Sep 3;14(9):880. doi: 10.3390/pathogens14090880 (PMC12472360; doi:10.3390/pathogens14090880)
Supplement: Supplementary file 1 [file pathogens-14-00880-s001.zip › pathogens-3811111-supplementary.pdf]

# Supplementary files

Supplementary Table S1 Estimated new cases and age-standardized incidence rates (ASIR) for HPV-related cancers by site and sex in 2022 ........ 2

Supplementary Table S2 Estimated new cases and age-standardized mortality rates (ASMR) for HPV-related cancers by site and sex in 2022. ........ 18

Supplementary Table S3 Definitions of HDI regions........ 30

Supplementary Table S4 International variations in average annual percentage change (AAPC) of HPV-related cancer incidence rates by site and sex ........ 32

**Supplementary Table S1: Estimated new cases and age-standardized incidence rates (ASIR) for HPV-related cancers by site and sex in 2022**

| Countries | Total, both sexes | | Total, males | | Anus, males | | Penis, males | | Head and neck, males | |
|---|---|---|---|---|---|---|---|---|---|---|
| | Cases | ASIR (World) | Cases | ASIR (World) | Cases | ASIR (World) | Cases | ASIR (World) | Cases | ASIR (World) |
| **World** | 1505394 | 20.9 | 582666 | 12.5 | 24035 | 0.51 | 37695 | 0.79 | 520936 | 11.2 |
| **HDI** | | | | | | | | | | |
| Very high HDI country | 413498 | 19.1 | 196583 | 14.0 | 10377 | 0.74 | 10778 | 0.69 | 175428 | 12.6 |
| High HDI country | 467827 | 16.9 | 142891 | 7.5 | 6707 | 0.35 | 12157 | 0.63 | 124027 | 6.53 |
| Medium HDI country | 487713 | 29.8 | 214250 | 20.3 | 4211 | 0.4 | 13170 | 1.3 | 196869 | 18.6 |
| Low HDI country | 136356 | 25.2 | 28942 | 8.7 | 2740 | 0.81 | 1590 | 0.45 | 24612 | 7.39 |
| **Continents** | | | | | | | | | | |
| Africa | 170237 | 25.1 | 25135 | 6.4 | 3274 | 0.79 | 2081 | 0.49 | 19780 | 5.15 |
| Latin America and the Caribbean | 125582 | 20.3 | 42467 | 11.2 | 2008 | 0.53 | 5194 | 1.3 | 35265 | 9.4 |
| Northern America | 96305 | 20.1 | 49963 | 15.8 | 3334 | 1.1 | 1964 | 0.54 | 44665 | 14.2 |
| Europe | 232245 | 22.5 | 112690 | 17.2 | 5470 | 0.8 | 6732 | 0.9 | 100488 | 15.5 |
| Oceania | 10602 | 23.5 | 5171 | 17.1 | 343 | 1.1 | 198 | 0.53 | 4630 | 15.5 |
| Asia | 870928 | 20.0 | 347439 | 12.2 | 9604 | 0.33 | 21531 | 0.74 | 316304 | 11.1 |
| **World Bank Classification** | | | | | | | | | | |
| High income | 307287 | 18.6 | 152773 | 13.9 | 9249 | 0.85 | 8330 | 0.65 | 135194 | 12.4 |
| Upper middle income | 473495 | 16.8 | 152815 | 8.2 | 6258 | 0.33 | 12736 | 0.68 | 133821 | 7.2 |
| Lower middle income | 641414 | 26.5 | 264834 | 17.1 | 7395 | 0.48 | 15263 | 0.99 | 242176 | 15.6 |
| Low income | 76174 | 23.5 | 10197 | 5.5 | 1032 | 0.52 | 1083 | 0.56 | 8082 | 4.38 |
| **Countries** | | | | | | | | | | |
| India | 354044 | 32.7 | 172220 | 23.9 | 2930 | 0.41 | 10443 | 1.5 | 985 | 9.28 |
| China | 243612 | 14.2 | 66868 | 5.5 | 3087 | 0.25 | 5973 | 0.49 | 223 | 8.89 |
| United States of America | 86301 | 20.3 | 45199 | 16.0 | 3069 | 1.1 | 1671 | 0.53 | 1192 | 5.41 |
| Indonesia | 52661 | 22.7 | 10435 | 7.5 | 491 | 0.37 | 1098 | 0.77 | 713 | 9.6 |
| Brazil | 50979 | 23.5 | 22984 | 17.6 | 1075 | 0.8 | 1688 | 1.3 | 2195 | 8.1 |
| Russian Federation | 44495 | 25.9 | 17992 | 17.7 | 407 | 0.41 | 785 | 0.76 | 187 | 9.9 |

| | | | | | | | | | | |
|---|---|---|---|---|---|---|---|---|---|---|
| Bangladesh | 37717 | 31.6 | 20718 | 26.3 | 257 | 0.32 | 684 | 0.83 | 3163 | 14.9 |
| Japan | 33472 | 16.5 | 14943 | 9.3 | 590 | 0.36 | 584 | 0.27 | 982 | 11.4 |
| Germany | 27261 | 19.6 | 12064 | 13.3 | 1153 | 1.3 | 856 | 0.87 | 502 | 9.07 |
| Pakistan | 27177 | 21 | 15647 | 18.0 | 428 | 0.48 | 318 | 0.34 | 17 | 7.7 |
| France (metropolitan) | 21312 | 23 | 11510 | 19.5 | 595 | 1 | 467 | 0.65 | 35 | 4.9 |
| United Kingdom | 19255 | 21.5 | 9533 | 15.7 | 604 | 0.98 | 799 | 1.2 | 19777 | 25.1 |
| Nigeria | 18751 | 24.3 | 2547 | 4.9 | 880 | 1.7 | 23 | 0.02 | 24 | 8.4 |
| Italy | 17417 | 16.3 | 8974 | 13.5 | 653 | 0.97 | 711 | 0.84 | 1872 | 27.6 |
| Thailand | 17322 | 19.6 | 5661 | 10.1 | 168 | 0.3 | 641 | 1.2 | 1497 | 13.6 |
| South Africa | 15404 | 34.8 | 2821 | 12.0 | 231 | 0.89 | 360 | 1.4 | 7 | 3.6 |
| Mexico | 15289 | 13.5 | 2967 | 4.3 | 156 | 0.23 | 758 | 1.1 | 81 | 2.53 |
| Poland | 14707 | 27.1 | 7485 | 23.3 | 253 | 0.72 | 509 | 1.4 | 34 | 8.8 |
| Ukraine | 13880 | 25.5 | 6749 | 21.5 | 165 | 0.5 | 235 | 0.71 | 207 | 3.7 |
| Philippines | 13666 | 17.4 | 3464 | 7.8 | 135 | 0.29 | 157 | 0.32 | 385 | 13 |
| Tanzania, United Republic of | 12975 | 55.1 | 1152 | 9.3 | 292 | 1.8 | 138 | 1.2 | 101 | 13.7 |
| Spain | 12362 | 16.6 | 6891 | 14.4 | 303 | 0.66 | 482 | 0.85 | 20221 | 15.5 |
| Myanmar | 12226 | 27.1 | 3823 | 15.4 | 99 | 0.43 | 347 | 1.5 | 1 | 0.32 |
| Viet Nam | 11152 | 12 | 5024 | 9.0 | 247 | 0.46 | 413 | 0.74 | 770 | 12.1 |
| Ethiopia | 10910 | 20.2 | 1191 | 3.4 | 161 | 0.44 | 39 | 0.11 | 224 | 4.16 |
| Congo, Democratic Republic of | 10703 | 28.1 | 998 | 4.4 | 234 | 0.96 | 144 | 0.63 | 138 | 5.64 |
| Canada | 9983 | 18.7 | 4748 | 13.0 | 265 | 0.74 | 293 | 0.68 | 497 | 8.4 |
| Türkiye | 9734 | 12.2 | 5495 | 11.4 | 130 | 0.26 | 18 | 0.04 | 328 | 4.64 |
| Romania | 9732 | 38.4 | 5040 | 31.6 | 127 | 0.73 | 183 | 1.1 | 4190 | 11.6 |
| Argentina | 8847 | 20.2 | 2769 | 10.1 | 104 | 0.41 | 470 | 1.6 | 19 | 9.06 |
| Colombia | 8605 | 17.4 | 2282 | 7.4 | 150 | 0.49 | 513 | 1.6 | 39 | 3.36 |
| Uganda | 8364 | 47.4 | 1015 | 11.6 | 84 | 1.1 | 316 | 3.1 | 67 | 1.29 |
| Republic of Korea | 7924 | 11.3 | 3294 | 6.4 | 144 | 0.29 | 109 | 0.2 | 518 | 3.57 |
| Kenya | 7564 | 31.1 | 869 | 7.0 | 65 | 0.58 | 51 | 0.45 | 57808 | 4.75 |
| Peru | 6857 | 22.4 | 1012 | 4.9 | 86 | 0.44 | 281 | 1.3 | 1619 | 5.3 |
| Venezuela | 6548 | 25.8 | 1785 | 11.5 | 99 | 0.64 | 268 | 1.7 | 11 | 4.7 |
| Australia | 6499 | 19.9 | 3552 | 16.6 | 262 | 1.2 | 127 | 0.51 | 620 | 2.78 |

| | | | | | | | | | | |
|---|---|---|---|---|---|---|---|---|---|---|
| Mozambique | 6466 | 41.2 | 464 | 5.7 | 20 | 0.15 | 136 | 1.6 | 23 | 1.48 |
| Iran, Islamic Republic of | 5821 | 8.3 | 3235 | 7.1 | 140 | 0.29 | 35 | 0.08 | 165 | 4.57 |
| Sri Lanka | 5760 | 23.7 | 3295 | 23.0 | 82 | 0.55 | 103 | 0.69 | 254 | 3.28 |
| Egypt | 5581 | 8.3 | 2658 | 6.5 | 230 | 0.55 | 11 | 0.03 | 598 | 15.6 |
| Malawi | 5351 | 57.1 | 303 | 6.2 | 22 | 0.43 | 136 | 2.5 | 2985 | 29.3 |
| Cuba | 5256 | 33.6 | 3184 | 30.9 | 36 | 0.33 | 163 | 1.3 | 75 | 7.8 |
| Madagascar | 5253 | 37.5 | 680 | 8.4 | 108 | 1.3 | 9 | 0.11 | 1423 | 14.4 |
| Morocco | 5200 | 16.1 | 1724 | 8.4 | 138 | 0.67 | 7 | 0.03 | 808 | 14.4 |
| The Netherlands | 4540 | 17.6 | 2045 | 11.2 | 172 | 1 | 181 | 0.86 | 13 | 3.2 |
| Uzbekistan | 4394 | 17.6 | 1119 | 7.7 | 115 | 0.76 | 10 | 0.08 | 490 | 8.6 |
| Angola | 4237 | 32.7 | 797 | 11.0 | 22 | 0.32 | 62 | 1.1 | 301 | 3.3 |
| Zambia | 4228 | 59.7 | 311 | 7.6 | 11 | 0.22 | 133 | 3 | 2417 | 5.93 |
| Zimbabwe | 4134 | 60.4 | 299 | 9.1 | 33 | 1 | 112 | 3 | 95 | 2.87 |
| Portugal | 4049 | 26.8 | 2360 | 25.6 | 91 | 0.78 | 117 | 0.98 | 27 | 7.3 |
| Hungary | 4032 | 30.4 | 2119 | 26.5 | 21 | 0.23 | 88 | 1 | 38 | 3.59 |
| Ghana | 3934 | 23.7 | 496 | 4.9 | 83 | 0.64 | 24 | 0.3 | 159 | 14.9 |
| Nepal | 3929 | 19.1 | 1272 | 10.8 | 26 | 0.19 | 174 | 1.5 | 8 | 2.81 |
| Malaysia | 3791 | 13.6 | 1217 | 6.8 | 25 | 0.13 | 72 | 0.38 | 991 | 2.87 |
| Algeria | 3503 | 10.4 | 1281 | 5.8 | 83 | 0.36 | 6 | 0.03 | 26 | 5.89 |
| Czechia | 3395 | 21.6 | 1669 | 16.7 | 107 | 1 | 139 | 1.3 | 489 | 8.7 |
| Kazakhstan | 3354 | 19.8 | 1075 | 11.5 | 24 | 0.26 | 38 | 0.42 | 10448 | 17.8 |
| Korea, Democratic People Republic of | 3334 | 12.1 | 843 | 5.1 | 14 | 0.09 | 57 | 0.38 | 57 | 18.3 |
| Belgium | 3273 | 19.7 | 1689 | 15.2 | 97 | 0.85 | 95 | 0.75 | 137 | 20.9 |
| Cameroon | 3195 | 29.2 | 387 | 5.4 | 50 | 0.68 | 9 | 0.11 | 36 | 9.8 |
| Belarus | 3139 | 26.1 | 1978 | 29.1 | 46 | 0.66 | 60 | 0.86 | 15 | 10.5 |
| Mali | 2952 | 36.7 | 213 | 4.5 | 33 | 0.66 | 6 | 0.11 | 26 | 13.7 |
| Côte d'Ivoire | 2928 | 25.6 | 295 | 3.8 | 37 | 0.43 | 4 | 0.05 | 78 | 10.7 |
| Afghanistan | 2796 | 16.5 | 1064 | 10.0 | 67 | 0.58 | 12 | 0.1 | 101 | 6.88 |
| Guinea | 2782 | 45.6 | 113 | 3.6 | 16 | 0.49 | 5 | 0.09 | 486 | 16.8 |
| Bolivia | 2774 | 31.6 | 405 | 6.2 | 4 | 0.05 | 194 | 2.4 | 10055 | 11.1 |
| Serbia | 2757 | 23.3 | 1352 | 17.7 | 65 | 0.79 | 63 | 0.76 | 389 | 3.93 |

| | | | | | | | | | | |
|---|---|---|---|---|---|---|---|---|---|---|
| Chile | 2677 | 12.3 | 668 | 4.6 | 52 | 0.39 | 98 | 0.67 | 1267 | 11.53 |
| Greece | 2653 | 15.2 | 1542 | 13.9 | 108 | 1.1 | 167 | 1.3 | 7 | 6.21 |
| Ecuador | 2645 | 17.6 | 480 | 5.1 | 40 | 0.42 | 139 | 1.4 | 146 | 2.22 |
| Papua New Guinea | 2603 | 51.3 | 995 | 33.8 | 52 | 2.4 | 44 | 2.4 | 92 | 3.01 |
| Senegal | 2457 | 30.2 | 177 | 4.1 | 36 | 0.81 | 3 | 0.04 | 9 | 2.04 |
| Sudan | 2417 | 11.4 | 657 | 4.9 | 12 | 0.04 | 20 | 0.18 | 21 | 5.33 |
| Sweden | 2388 | 16.7 | 953 | 9.2 | 55 | 0.53 | 143 | 1.2 | 210 | 4.8 |
| Cambodia | 2276 | 20.5 | 618 | 10.3 | 24 | 0.38 | 97 | 1.5 | 296 | 8.18 |
| Slovakia | 2230 | 31.5 | 1259 | 28.6 | 61 | 1.2 | 46 | 0.98 | 2010 | 25.3 |
| Austria | 2229 | 16.6 | 1129 | 12.9 | 66 | 0.74 | 81 | 0.79 | 24 | 7.8 |
| Bulgaria | 2114 | 21.8 | 865 | 13.5 | 30 | 0.46 | 65 | 0.9 | 158847 | 22 |
| Guatemala | 2087 | 18.2 | 167 | 2.5 | 0 | 0 | 21 | 0.3 | 8846 | 6.38 |
| Switzerland | 2020 | 15.1 | 1060 | 12.4 | 83 | 0.9 | 50 | 0.46 | 3060 | 6.71 |
| Denmark | 1983 | 24.5 | 937 | 16.5 | 55 | 0.95 | 74 | 1.1 | 581 | 5.46 |
| Burundi | 1826 | 39.2 | 231 | 9.3 | 29 | 1.2 | 64 | 2.5 | 507 | 12.5 |
| Dominican Republic | 1790 | 19.6 | 565 | 9.9 | 9 | 0.15 | 66 | 1.1 | 295 | 5.13 |
| Paraguay | 1730 | 32.2 | 498 | 14.9 | 22 | 0.67 | 120 | 3.4 | 7610 | 11.7 |
| Somalia | 1587 | 24.9 | 211 | 5.4 | 17 | 0.44 | 3 | 0.07 | 87 | 4.86 |
| Burkina Faso | 1506 | 17.1 | 244 | 4.3 | 20 | 0.18 | 0 | 0 | 13769 | 8.7 |
| Honduras | 1453 | 22.2 | 393 | 10.6 | 18 | 0.46 | 79 | 2 | 283 | 7.5 |
| Norway | 1425 | 19.7 | 576 | 10.8 | 30 | 0.58 | 68 | 1.2 | 1013 | 10.8 |
| Chad | 1390 | 19.7 | 76 | 1.6 | 9 | 0.28 | 0 | 0 | 753 | 5.97 |
| Tunisia | 1374 | 11.8 | 715 | 9.9 | 48 | 0.65 | 9 | 0.12 | 772 | 4.6 |
| Haiti | 1337 | 18.1 | 326 | 7.6 | 60 | 1.5 | 56 | 1.3 | 91 | 3.65 |
| Azerbaijan | 1315 | 13.9 | 571 | 10.3 | 68 | 1.2 | 1 | 0.02 | 154 | 6.74 |
| Moldova | 1313 | 28.6 | 795 | 29.3 | 3 | 0.1 | 17 | 0.62 | 202 | 7.24 |
| Iraq | 1260 | 6.7 | 607 | 5.7 | 23 | 0.21 | 3 | 0.03 | 272 | 18.4 |
| Rwanda | 1245 | 19.3 | 254 | 7.2 | 3 | 0.08 | 91 | 2.6 | 181 | 4.71 |
| Finland | 1193 | 13.5 | 552 | 9.7 | 20 | 0.34 | 43 | 0.68 | 37 | 5.72 |
| Croatia | 1192 | 19.4 | 653 | 17.0 | 20 | 0.53 | 35 | 0.87 | 46 | 2.88 |
| Saudi Arabia | 1170 | 5.2 | 559 | 3.2 | 60 | 0.31 | 4 | 0.03 | 193 | 7.18 |

| | | | | | | | | | | |
|---|---|---|---|---|---|---|---|---|---|---|
| Ireland | 1113 | 18.7 | 586 | 14.4 | 28 | 0.66 | 51 | 1.2 | 482 | 22.2 |
| Niger | 1075 | 11.1 | 255 | 4.3 | 91 | 1.4 | 4 | 0.08 | 36 | 6.4 |
| South Sudan | 1042 | 20.6 | 168 | 5.4 | 14 | 0.42 | 25 | 0.82 | 563 | 7 |
| Singapore | 1008 | 12.4 | 460 | 7.9 | 43 | 0.71 | 26 | 0.45 | 145 | 3.23 |
| Georgia | 1006 | 19.6 | 525 | 18.0 | 11 | 0.33 | 28 | 0.88 | 1120 | 6.24 |
| Lithuania | 972 | 26.1 | 519 | 23.7 | 10 | 0.42 | 27 | 1.1 | 9 | 4.5 |
| Nicaragua | 965 | 19.9 | 141 | 5.2 | 3 | 0.12 | 42 | 1.5 | 174 | 3.74 |
| Benin | 954 | 17.3 | 126 | 3.9 | 36 | 0.99 | 9 | 0.39 | 41 | 8.69 |
| Uruguay | 947 | 23.6 | 383 | 15.3 | 24 | 0.94 | 33 | 1.2 | 44 | 3.11 |
| Syrian Arab Republic | 932 | 8.2 | 460 | 6.7 | 11 | 0.15 | 5 | 0.08 | 84 | 8.7 |
| Yemen | 931 | 7.4 | 435 | 6.0 | 15 | 0.2 | 0 | 0 | 2053 | 3.01 |
| Israel | 925 | 10.2 | 329 | 5.7 | 25 | 0.42 | 9 | 0.14 | 775 | 28.6 |
| New Zealand | 921 | 14.7 | 482 | 11.6 | 29 | 0.62 | 17 | 0.35 | 62 | 4.95 |
| El Salvador | 880 | 15.2 | 123 | 3.7 | 0 | 0 | 28 | 0.83 | 54 | 10.3 |
| Liberia | 816 | 31.2 | 55 | 3.4 | 8 | 0.49 | 1 | 0.03 | 1579 | 7.67 |
| Lao People's Democratic Republic | 793 | 17.2 | 241 | 8.7 | 4 | 0.12 | 35 | 1.3 | 308 | 3.99 |
| Bosnia Herzegovina | 787 | 18.9 | 396 | 13.4 | 5 | 0.17 | 6 | 0.18 | 3377 | 13.5 |
| Turkmenistan | 781 | 17.9 | 219 | 9.1 | 20 | 0.78 | 0 | 0 | 109 | 17 |
| Puerto Rico | 769 | 18.9 | 403 | 15.3 | 19 | 0.73 | 45 | 1.6 | 1072 | 9.12 |
| Togo | 749 | 19.7 | 150 | 6.7 | 32 | 1.3 | 3 | 0.29 | 31 | 16.2 |
| Kyrgyzstan | 744 | 16.2 | 162 | 7.0 | 5 | 0.19 | 3 | 0.1 | 436 | 10.6 |
| Lesotho | 702 | 51.2 | 60 | 9.0 | 1 | 0.14 | 22 | 3.1 | 96 | 3.54 |
| Latvia | 686 | 27.4 | 299 | 20.1 | 8 | 0.46 | 19 | 1.2 | 160 | 2.84 |
| Costa Rica | 665 | 12.7 | 214 | 5.9 | 8 | 0.21 | 41 | 1.1 | 1644 | 3.21 |
| Botswana | 663 | 42.5 | 139 | 18.0 | 4 | 0.4 | 34 | 3.9 | 162 | 9.46 |
| Slovenia | 601 | 19.1 | 319 | 15.6 | 3 | 0.15 | 14 | 0.55 | 478 | 9 |
| Mauritania | 589 | 25.1 | 60 | 4.2 | 16 | 1.1 | 0 | 0 | 108 | 4.32 |
| Panama | 585 | 14.7 | 131 | 4.8 | 5 | 0.2 | 29 | 1 | 14901 | 17.14 |
| Namibia | 571 | 42.1 | 130 | 19.9 | 5 | 0.66 | 16 | 2.2 | 97 | 3.6 |
| Libya | 569 | 12 | 203 | 7.4 | 10 | 0.26 | 0 | 0 | 899 | 29 |
| Jamaica | 548 | 19.8 | 122 | 6.7 | 16 | 0.87 | 19 | 0.96 | 356 | 10.8 |

| | | | | | | | | | | |
|---|---|---|---|---|---|---|---|---|---|---|
| Albania | 529 | 14.7 | 242 | 9.7 | 7 | 0.28 | 12 | 0.51 | 645 | 3.15 |
| Jordan | 524 | 8.9 | 295 | 7.8 | 12 | 0.32 | 0 | 0 | 3172 | 7.18 |
| Congo, Republic of | 495 | 19.6 | 38 | 2.4 | 14 | 0.83 | 1 | 0.05 | 6723 | 21.2 |
| Sierra Leone | 491 | 11.3 | 3 | 0.1 | 2 | 0.06 | 0 | 0 | 2152 | 23.8 |
| Lebanon | 488 | 8.3 | 196 | 5.1 | 6 | 0.16 | 9 | 0.23 | 339 | 13 |
| Mongolia | 485 | 20.1 | 78 | 6.3 | 8 | 0.7 | 8 | 0.67 | 50 | 2.76 |
| Eswatini | 484 | 77.4 | 31 | 9.9 | 1 | 0.28 | 22 | 6.8 | 3041 | 5.9 |
| Tajikistan | 449 | 8 | 90 | 3.0 | 22 | 0.6 | 0 | 0 | 4730 | 29.8 |
| Armenia | 415 | 12.1 | 200 | 10.5 | 6 | 0.3 | 7 | 0.33 | 16800 | 16.5 |
| Gabon | 397 | 33 | 91 | 12.2 | 12 | 1.4 | 1 | 0.13 | 160 | 4.49 |
| Estonia | 393 | 21.9 | 178 | 16.6 | 5 | 0.58 | 14 | 1.1 | 14 | 11.52 |
| Central African Republic | 389 | 20.6 | 47 | 4.0 | 7 | 0.49 | 1 | 0.12 | 6 | 7.2 |
| The Republic of the Gambia | 343 | 28.9 | 16 | 2.5 | 0 | 0 | 5 | 0.82 | 2 | 3.3 |
| North Macedonia | 342 | 12.8 | 180 | 10.4 | 6 | 0.33 | 12 | 0.57 | 495 | 2.87 |
| Trinidad and Tobago | 319 | 20.4 | 82 | 8.3 | 10 | 1.1 | 9 | 0.9 | 138 | 3.28 |
| United Arab Emirates | 318 | 7.5 | 127 | 3.9 | 5 | 0.18 | 3 | 0.11 | 1224 | 16.1 |
| Eritrea | 282 | 16.2 | 40 | 3.7 | 1 | 0.04 | 1 | 0.11 | 1 | 0.04 |
| Mauritius | 275 | 17.7 | 92 | 9.5 | 4 | 0.38 | 4 | 0.39 | 391 | 6.69 |
| France, La Réunion | 254 | 24.4 | 144 | 21.9 | 1 | 0.16 | 6 | 0.81 | 1152 | 26.4 |
| Oman | 246 | 8.8 | 129 | 5.3 | 20 | 0.91 | 1 | 0.03 | 302 | 14.9 |
| Guinea-Bissau | 242 | 27.5 | 11 | 2.5 | 2 | 0.46 | 0 | 0 | 13 | 5.49 |
| Fiji | 225 | 32.1 | 31 | 7.2 | 0 | 0 | 5 | 1.3 | 191 | 4.91 |
| Kuwait | 190 | 5.9 | 98 | 3.8 | 7 | 0.16 | 0 | 0 | 2230 | 9.7 |
| Cyprus | 189 | 12.7 | 89 | 9.1 | 4 | 0.4 | 10 | 0.9 | 129 | 4.11 |
| Gaza Strip and West Bank | 189 | 7.9 | 104 | 7.1 | 3 | 0.23 | 0 | 0 | 6106 | 12.9 |
| Comoros | 182 | 40.1 | 13 | 5.4 | 0 | 0 | 2 | 0.68 | 3110 | 21.8 |
| Equatorial Guinea | 169 | 28.5 | 32 | 8.3 | 5 | 1 | 0 | 0 | 625 | 4.64 |
| Guyana | 160 | 25.4 | 25 | 6.3 | 0 | 0 | 4 | 0.99 | 15 | 4.99 |
| Montenegro | 155 | 19 | 60 | 11.4 | 4 | 0.77 | 2 | 0.34 | 755 | 7.5 |
| Suriname | 111 | 22.6 | 19 | 6.4 | 0 | 0 | 4 | 1.4 | 927 | 11 |
| France, Guadeloupe | 106 | 19.9 | 60 | 19.0 | 0 | 0 | 3 | 0.65 | 444 | 6.45 |

| | | | | | | | | | | |
|---|---|---|---|---|---|---|---|---|---|---|
| Bhutan | 99 | 18.2 | 38 | 10.0 | 0 | 0 | 4 | 1.2 | 68 | 2.42 |
| Solomon Islands | 99 | 24.6 | 17 | 7.0 | 0 | 0 | 4 | 1.5 | 722 | 6.28 |
| Malta | 98 | 14.1 | 49 | 10.5 | 3 | 0.48 | 5 | 1.3 | 4852 | 8.6 |
| Luxembourg | 96 | 11.5 | 42 | 7.4 | 4 | 0.71 | 2 | 0.31 | 1692 | 9.3 |
| Djibouti | 95 | 14.7 | 13 | 3.2 | 0 | 0 | 0 | 0 | 11 | 1.71 |
| Timor-Leste | 93 | 12.2 | 10 | 2.3 | 0 | 0 | 0 | 0 | 10 | 2.26 |
| Qatar | 86 | 6.1 | 56 | 3.3 | 4 | 0.28 | 2 | 0.27 | 115 | 5.1 |
| Cape Verde | 78 | 19.6 | 23 | 10.9 | 1 | 0.51 | 3 | 1.3 | 63 | 6.3 |
| France, Martinique | 78 | 12.7 | 40 | 10.7 | 0 | 0 | 4 | 0.88 | 658 | 9.09 |
| Barbados | 76 | 18.3 | 27 | 9.8 | 2 | 0.97 | 1 | 0.41 | 5347 | 11.09 |
| Bahamas | 69 | 18 | 23 | 10.3 | 4 | 1.9 | 2 | 0.7 | 199 | 8.3 |
| New Caledonia | 66 | 22.7 | 31 | 16.2 | 0 | 0 | 0 | 0 | 615 | 7.4 |
| Iceland | 65 | 14.9 | 27 | 8.6 | 0 | 0 | 3 | 0.83 | 6349 | 20.3 |
| Bahrain | 63 | 6.3 | 38 | 5.3 | 3 | 0.39 | 0 | 0 | 119 | 3.65 |
| Maldives | 60 | 15.8 | 9 | 4.5 | 0 | 0 | 0 | 0 | 8130 | 13.5 |
| Brunei Darussalam | 56 | 13.7 | 1 | 0.3 | 0 | 0 | 0 | 0 | 40459 | 14.4 |
| French Polynesia | 46 | 16.4 | 26 | 13.7 | 0 | 0 | 0 | 0 | 326 | 13.2 |
| Saint Lucia | 43 | 22.7 | 19 | 15.2 | 0 | 0 | 5 | 3.7 | 994 | 6.83 |
| Belize | 41 | 14 | 7 | 3.6 | 0 | 0 | 0 | 0 | 4 | 3.2 |
| French Guyana | 38 | 17.1 | 18 | 12.6 | 1 | 0.63 | 2 | 1.5 | 1418 | 9.2 |
| Vanuatu | 34 | 18.1 | 5 | 4.1 | 0 | 0 | 1 | 0.89 | 4364 | 7.8 |
| Guam | 27 | 16.9 | 7 | 6.2 | 0 | 0 | 0 | 0 | 420 | 5.78 |
| Samoa | 17 | 13.4 | 6 | 7.2 | 0 | 0 | 0 | 0 | 167 | 4.41 |
| Sao Tome and Principe | 16 | 15.1 | 2 | 3.3 | 0 | 0 | 0 | 0 | 154 | 5.07 |

**Supplementary Table S1: Estimated new cases and age-standardized incidence rates (ASIR) for HPV-related cancers by site and sex in 2022 (Continued)**

| Countries | Total, females | | Cervix uteri, females | | Vagina, females | | Vulva, females | | Anus, females | | Head and neck, females | |
|---|---|---|---|---|---|---|---|---|---|---|---|---|
| | Cases | ASIR (World) | Cases | ASIR (World) | Cases | ASIR (World) | Cases | ASIR (World) | Cases | ASIR (World) | Cases | ASIR (World) |
| **World** | 922728 | 19.0 | 662044 | 14.1 | 18817 | 0.36 | 47328 | 0.83 | 30271 | 0.57 | 164268 | 3.14 |
| **HDI** | | | | | | | | | | | | |
| Very high HDI country | 216915 | 15.6 | 107148 | 9.3 | 5877 | 0.33 | 27558 | 1.4 | 17104 | 1.1 | 59228 | 3.42 |
| High HDI country | 324936 | 16.9 | 265503 | 14.1 | 5188 | 0.25 | 10718 | 0.5 | 7331 | 0.34 | 36196 | 1.72 |
| Medium HDI country | 273463 | 24.7 | 200389 | 18 | 6238 | 0.57 | 5213 | 0.47 | 3731 | 0.34 | 57892 | 5.35 |
| Low HDI country | 107414 | 28.7 | 89004 | 23.8 | 1514 | 0.4 | 3839 | 1 | 2105 | 0.56 | 10952 | 2.96 |
| **Continents** | | | | | | | | | | | | |
| Africa | 145102 | 30.6 | 125699 | 26.4 | 2054 | 0.43 | 5629 | 1.2 | 3082 | 0.65 | 8638 | 1.88 |
| Latin America and the Caribbean | 83115 | 19.4 | 63171 | 15.1 | 1618 | 0.36 | 3655 | 0.75 | 3780 | 0.83 | 10891 | 2.33 |
| Northern America | 46342 | 14.9 | 15654 | 6.4 | 1664 | 0.43 | 7187 | 1.9 | 6578 | 1.9 | 15259 | 4.31 |
| Europe | 119555 | 17.4 | 58219 | 10.6 | 3164 | 0.34 | 17651 | 1.7 | 8749 | 1.1 | 31772 | 3.66 |
| Oceania | 5431 | 18.5 | 2476 | 9.6 | 148 | 0.45 | 497 | 1.3 | 487 | 1.5 | 1823 | 5.65 |
| Asia | 523489 | 18.0 | 397082 | 13.9 | 10171 | 0.34 | 12717 | 0.4 | 7597 | 0.24 | 95922 | 3.14 |
| **World Bank Classification** | | | | | | | | | | | | |
| High income | 154514 | 14.5 | 62809 | 7.5 | 4706 | 0.34 | 23458 | 1.6 | 15235 | 1.3 | 48306 | 3.77 |
| Upper middle income | 320680 | 17.3 | 256507 | 14.2 | 5419 | 0.27 | 12149 | 0.56 | 7916 | 0.37 | 38689 | 1.85 |
| Lower middle income | 376580 | 22.8 | 280854 | 16.9 | 7628 | 0.47 | 9377 | 0.57 | 5684 | 0.35 | 73037 | 4.52 |
| Low income | 65977 | 29.0 | 57754 | 25.4 | 948 | 0.42 | 2237 | 0.95 | 1200 | 0.52 | 3838 | 1.73 |
| **Countries** | | | | | | | | | | | | |
| India | 181824 | 25.3 | 127526 | 17.7 | 5000 | 0.7 | 3112 | 0.43 | 2245 | 0.31 | 413 | 3.83 |
| China | 176744 | 15.8 | 150659 | 13.8 | 2685 | 0.23 | 4117 | 0.32 | 2481 | 0.19 | 65 | 2.15 |
| Indonesia | 42226 | 26.7 | 36964 | 23.3 | 462 | 0.3 | 1307 | 0.84 | 299 | 0.19 | 202 | 0.88 |
| United States of America | 41102 | 14.8 | 13920 | 6.3 | 1436 | 0.42 | 6228 | 1.8 | 5955 | 1.9 | 430 | 4.91 |
| Brazil | 27995 | 18.4 | 18715 | 12.7 | 462 | 0.28 | 1432 | 0.84 | 2063 | 1.3 | 743 | 2.08 |
| Russian Federation | 26503 | 22.8 | 18369 | 17.6 | 533 | 0.35 | 2104 | 1.1 | 959 | 0.69 | 28 | 0.89 |

| | | | | | | | | | | | | |
|---|---|---|---|---|---|---|---|---|---|---|---|---|
| Japan | 18529 | 15.7 | 10958 | 12.5 | 378 | 0.19 | 1341 | 0.49 | 501 | 0.21 | 1152 | 4.69 |
| Bangladesh | 16999 | 20.8 | 9640 | 11.3 | 238 | 0.31 | 287 | 0.37 | 115 | 0.15 | 345 | 3.32 |
| Nigeria | 16204 | 30.8 | 13676 | 26.2 | 173 | 0.28 | 1056 | 1.9 | 423 | 0.83 | 139 | 2.08 |
| Germany | 15197 | 16.4 | 4544 | 7.1 | 592 | 0.44 | 4633 | 3.7 | 1540 | 1.7 | 0 | 0 |
| South Africa | 12583 | 39.7 | 10532 | 33.2 | 230 | 0.74 | 672 | 2 | 270 | 0.81 | 6 | 1.2 |
| Mexico | 12322 | 15.6 | 10348 | 13.2 | 246 | 0.31 | 475 | 0.56 | 192 | 0.23 | 6719 | 8.71 |
| Tanzania, United Republic of | 11823 | 70.0 | 10868 | 64.8 | 115 | 0.66 | 289 | 1.3 | 264 | 1.6 | 4 | 0.64 |
| Thailand | 11661 | 19.2 | 8662 | 14.9 | 166 | 0.26 | 252 | 0.37 | 169 | 0.24 | 219 | 2.07 |
| Pakistan | 11530 | 13.5 | 4762 | 5.4 | 252 | 0.29 | 252 | 0.3 | 198 | 0.22 | 592 | 4.83 |
| Philippines | 10202 | 18.5 | 8549 | 15.5 | 121 | 0.22 | 190 | 0.35 | 106 | 0.19 | 0 | 0 |
| France (metropolitan) | 9802 | 15.6 | 3185 | 6.6 | 246 | 0.33 | 1078 | 1.1 | 1387 | 2.1 | 66 | 1.62 |
| United Kingdom | 9722 | 16.9 | 3235 | 7.5 | 237 | 0.34 | 1520 | 1.9 | 1325 | 2 | 12 | 3.99 |
| Ethiopia | 9719 | 26.1 | 8168 | 22.3 | 220 | 0.59 | 483 | 1.1 | 185 | 0.44 | 95 | 1.56 |
| Congo, Democratic Republic of | 9705 | 36.6 | 8705 | 32.9 | 118 | 0.45 | 332 | 1.2 | 261 | 1 | 76 | 2.04 |
| Italy | 8443 | 11.4 | 2479 | 5 | 280 | 0.29 | 1728 | 1.6 | 878 | 1.1 | 17 | 1.61 |
| Myanmar | 8403 | 25.7 | 7028 | 21.4 | 68 | 0.21 | 151 | 0.47 | 79 | 0.24 | 5323 | 3.27 |
| Uganda | 7349 | 57.2 | 6938 | 53.8 | 43 | 0.35 | 143 | 1.1 | 32 | 0.28 | 2 | 0.83 |
| Poland | 7222 | 18.3 | 4008 | 11.3 | 154 | 0.27 | 771 | 1.4 | 249 | 0.5 | 188 | 2.44 |
| Ukraine | 7131 | 19.0 | 5163 | 15.2 | 145 | 0.3 | 658 | 1.1 | 200 | 0.43 | 110 | 1.97 |
| Kenya | 6695 | 38.2 | 5845 | 32.8 | 59 | 0.36 | 163 | 0.94 | 177 | 1.1 | 50 | 1.57 |
| Colombia | 6323 | 18.4 | 4570 | 13.7 | 183 | 0.51 | 394 | 1.1 | 382 | 1 | 318 | 4.08 |
| Viet Nam | 6128 | 9.3 | 4612 | 7.1 | 62 | 0.09 | 188 | 0.28 | 295 | 0.42 | 124 | 1.81 |
| Argentina | 6078 | 20.6 | 4696 | 16.8 | 106 | 0.32 | 343 | 0.83 | 190 | 0.56 | 1695 | 4.22 |
| Mozambique | 6002 | 52.3 | 5456 | 47.8 | 48 | 0.38 | 266 | 2.2 | 114 | 1 | 7 | 2.52 |
| Peru | 5845 | 28.7 | 4809 | 23.9 | 100 | 0.45 | 276 | 1.2 | 158 | 0.76 | 19 | 1.27 |
| Spain | 5471 | 10.9 | 2020 | 5.4 | 196 | 0.26 | 925 | 1.3 | 288 | 0.55 | 156 | 2.99 |
| Canada | 5235 | 15.3 | 1730 | 6.6 | 228 | 0.53 | 959 | 2.2 | 623 | 1.7 | 200 | 1.18 |
| Malawi | 5048 | 76.2 | 4701 | 70.9 | 85 | 1.5 | 94 | 1.1 | 73 | 1.2 | 16802 | 1.29 |
| Venezuela | 4763 | 27.0 | 3965 | 22.7 | 114 | 0.64 | 93 | 0.49 | 218 | 1.2 | 794 | 2.12 |
| Romania | 4692 | 27.4 | 3368 | 21.7 | 68 | 0.31 | 472 | 1.8 | 134 | 0.59 | 0 | 0 |
| Republic of Korea | 4630 | 10.8 | 3397 | 8.6 | 95 | 0.17 | 204 | 0.35 | 233 | 0.39 | 289 | 1.08 |

| | | | | | | | | | | | |
|---|---|---|---|---|---|---|---|---|---|---|---|
| Madagascar | 4573 | 47.0 | 4060 | 41.8 | 54 | 0.54 | 206 | 1.9 | 126 | 1.4 | 17 | 1.04 |
| Türkiye | 4239 | 7.6 | 2593 | 4.8 | 90 | 0.16 | 313 | 0.5 | 130 | 0.21 | 60 | 1.43 |
| Zambia | 3917 | 76.4 | 3640 | 71.5 | 46 | 0.97 | 106 | 1.7 | 50 | 0.78 | 126 | 1.8 |
| Zimbabwe | 3835 | 74.0 | 3520 | 68.2 | 46 | 0.85 | 147 | 2.5 | 53 | 1 | 121 | 2.28 |
| Morocco | 3476 | 15.6 | 2644 | 12 | 66 | 0.31 | 206 | 0.88 | 129 | 0.57 | 626 | 4.99 |
| Angola | 3440 | 37.0 | 2823 | 30.4 | 82 | 0.65 | 89 | 0.91 | 16 | 0.17 | 18 | 1.69 |
| Ghana | 3438 | 30.3 | 3072 | 27 | 22 | 0.15 | 70 | 0.65 | 131 | 1.2 | 579 | 4.88 |
| Uzbekistan | 3275 | 18.5 | 2654 | 14.8 | 40 | 0.26 | 79 | 0.47 | 132 | 0.77 | 326 | 5.35 |
| Australia | 2947 | 13.6 | 898 | 5.3 | 104 | 0.43 | 409 | 1.5 | 384 | 1.7 | 7 | 1.6 |
| Egypt | 2923 | 6.3 | 1302 | 2.8 | 95 | 0.21 | 253 | 0.53 | 116 | 0.24 | 180 | 2.75 |
| Cameroon | 2808 | 37.0 | 2525 | 33.1 | 47 | 0.59 | 59 | 0.87 | 53 | 0.67 | 177 | 1.69 |
| Mali | 2739 | 48.4 | 2436 | 43.1 | 37 | 0.59 | 46 | 0.83 | 32 | 0.58 | 1157 | 2.49 |
| Guinea | 2669 | 57.8 | 2551 | 55 | 22 | 0.46 | 16 | 0.4 | 15 | 0.36 | 73 | 1.45 |
| Nepal | 2657 | 17.5 | 2169 | 14.2 | 36 | 0.23 | 25 | 0.16 | 18 | 0.11 | 7 | 2.34 |
| Côte d'Ivoire | 2633 | 35.7 | 2360 | 32 | 59 | 0.79 | 34 | 0.41 | 54 | 0.65 | 22 | 1.68 |
| Iran, Islamic Republic of | 2586 | 5.4 | 1265 | 2.5 | 55 | 0.12 | 48 | 0.1 | 100 | 0.23 | 46 | 3.34 |
| Malaysia | 2574 | 13.8 | 1913 | 10.3 | 49 | 0.26 | 55 | 0.29 | 74 | 0.38 | 3 | 0.69 |
| The Netherlands | 2495 | 15.4 | 756 | 6.7 | 60 | 0.3 | 537 | 2.6 | 174 | 0.98 | 663 | 1.7 |
| Korea, Democratic People Republic of | 2491 | 13.3 | 2153 | 11.8 | 30 | 0.14 | 56 | 0.26 | 24 | 0.1 | 22 | 4.68 |
| Sri Lanka | 2465 | 14.0 | 1579 | 9.2 | 56 | 0.35 | 59 | 0.25 | 57 | 0.3 | 266 | 3.58 |
| Bolivia | 2369 | 41.4 | 2213 | 38.7 | 28 | 0.49 | 27 | 0.51 | 6 | 0.12 | 3906 | 5.45 |
| Senegal | 2280 | 38.0 | 2064 | 34.3 | 30 | 0.48 | 38 | 0.69 | 31 | 0.53 | 6 | 1.23 |
| Kazakhstan | 2279 | 19.0 | 1824 | 15.5 | 51 | 0.4 | 127 | 0.92 | 33 | 0.24 | 17 | 2.22 |
| Algeria | 2222 | 9.8 | 1799 | 8 | 49 | 0.21 | 97 | 0.41 | 75 | 0.31 | 2 | 0.23 |
| Ecuador | 2165 | 21.2 | 1792 | 17.7 | 30 | 0.28 | 73 | 0.66 | 93 | 0.88 | 0 | 0 |
| Cuba | 2072 | 20.6 | 1122 | 12.8 | 66 | 0.55 | 112 | 0.98 | 146 | 1.3 | 4 | 2 |
| Chile | 2009 | 13.8 | 1559 | 11.3 | 57 | 0.32 | 127 | 0.6 | 66 | 0.36 | 23 | 3.29 |
| Guatemala | 1920 | 23.4 | 1761 | 21.5 | 15 | 0.19 | 14 | 0.2 | 6 | 0.09 | 19 | 1.25 |
| Hungary | 1913 | 20.7 | 964 | 12.6 | 71 | 0.52 | 251 | 1.6 | 33 | 0.25 | 64 | 1.22 |
| Sudan | 1760 | 12.2 | 1234 | 8.6 | 49 | 0.31 | 157 | 1.2 | 38 | 0.2 | 3888 | 3.47 |

| | | | | | | | | | | | | |
|---|---|---|---|---|---|---|---|---|---|---|---|---|
| Afghanistan | 1732 | 15.1 | 1218 | 10.3 | 20 | 0.18 | 38 | 0.37 | 43 | 0.38 | 143 | 1.25 |
| Czechia | 1726 | 16.4 | 658 | 8 | 61 | 0.43 | 272 | 1.9 | 156 | 1.2 | 339 | 2.22 |
| Portugal | 1689 | 15.9 | 897 | 11.1 | 47 | 0.27 | 196 | 0.78 | 112 | 0.7 | 1 | 0.82 |
| Cambodia | 1658 | 20.1 | 1274 | 15.2 | 10 | 0.12 | 39 | 0.5 | 17 | 0.22 | 124 | 1.43 |
| Papua New Guinea | 1608 | 44.1 | 1053 | 27.8 | 19 | 0.56 | 17 | 0.44 | 48 | 1.5 | 65 | 1.55 |
| Burundi | 1595 | 47.8 | 1457 | 43.6 | 14 | 0.43 | 49 | 1.3 | 25 | 0.85 | 5 | 0.92 |
| Belgium | 1584 | 14.7 | 531 | 6.3 | 53 | 0.44 | 265 | 1.9 | 143 | 1.2 | 2 | 0.39 |
| Sweden | 1435 | 16.1 | 561 | 8.6 | 37 | 0.33 | 222 | 1.7 | 155 | 1.5 | 108 | 1.97 |
| Serbia | 1405 | 18.2 | 906 | 13.4 | 36 | 0.36 | 136 | 1.3 | 52 | 0.46 | 71 | 1.58 |
| Somalia | 1376 | 31.4 | 1167 | 26.6 | 26 | 0.57 | 51 | 1.1 | 26 | 0.58 | 594 | 5.7 |
| Chad | 1314 | 27.5 | 1111 | 23.5 | 17 | 0.43 | 30 | 0.62 | 0 | 0 | 7 | 1.47 |
| Burkina Faso | 1262 | 20.2 | 988 | 15.9 | 29 | 0.47 | 41 | 0.47 | 94 | 1.4 | 43941 | 6.12 |
| Bulgaria | 1249 | 19.9 | 877 | 15.4 | 35 | 0.41 | 111 | 1.1 | 38 | 0.51 | 3194 | 2.03 |
| Paraguay | 1232 | 33.7 | 1115 | 30.6 | 1 | 0.03 | 39 | 0.96 | 43 | 1.2 | 1118 | 2.42 |
| Dominican Republic | 1225 | 19.6 | 967 | 15.6 | 57 | 0.92 | 10 | 0.13 | 11 | 0.18 | 277 | 2.05 |
| Belarus | 1161 | 13.8 | 668 | 9.3 | 24 | 0.24 | 169 | 1.3 | 81 | 0.85 | 159 | 3.71 |
| Greece | 1111 | 9.5 | 473 | 5.5 | 17 | 0.14 | 205 | 1 | 77 | 0.63 | 155 | 2.13 |
| Austria | 1100 | 12.4 | 409 | 5.9 | 38 | 0.33 | 175 | 1.4 | 133 | 1.4 | 3078 | 3.43 |
| Honduras | 1060 | 22.8 | 916 | 19.5 | 19 | 0.45 | 27 | 0.59 | 27 | 0.67 | 27 | 1.26 |
| Denmark | 1046 | 20.5 | 373 | 9.7 | 22 | 0.3 | 197 | 2.8 | 128 | 2.3 | 5351 | 2.35 |
| Haiti | 1011 | 19.3 | 869 | 16.6 | 23 | 0.49 | 3 | 0.06 | 8 | 0.13 | 64 | 1.64 |
| Rwanda | 991 | 21.6 | 866 | 18.9 | 11 | 0.24 | 64 | 1.4 | 16 | 0.36 | 244 | 1.91 |
| Slovakia | 971 | 19.9 | 557 | 12.9 | 17 | 0.27 | 122 | 1.9 | 55 | 0.84 | 451 | 3.02 |
| Switzerland | 960 | 10.3 | 269 | 4.1 | 27 | 0.24 | 142 | 0.97 | 181 | 1.9 | 228 | 0.97 |
| South Sudan | 874 | 25.0 | 749 | 21.4 | 9 | 0.27 | 30 | 0.87 | 18 | 0.5 | 26 | 1.81 |
| Norway | 849 | 19.1 | 376 | 10.9 | 24 | 0.44 | 103 | 1.7 | 73 | 1.4 | 51 | 1.58 |
| Benin | 828 | 21.3 | 701 | 18.1 | 13 | 0.39 | 35 | 0.88 | 13 | 0.35 | 117 | 4.08 |
| Nicaragua | 824 | 23.5 | 721 | 20.6 | 15 | 0.41 | 18 | 0.49 | 22 | 0.63 | 64 | 2.98 |
| Niger | 820 | 12.1 | 624 | 9.3 | 30 | 0.42 | 16 | 0.24 | 30 | 0.32 | 80 | 2.08 |
| Liberia | 761 | 42.2 | 717 | 39.6 | 10 | 0.55 | 4 | 0.23 | 6 | 0.32 | 9 | 0.94 |
| El Salvador | 757 | 17.7 | 627 | 15.2 | 10 | 0.17 | 28 | 0.48 | 19 | 0.43 | 24 | 1.49 |

| | | | | | | | | | | | | |
|---|---|---|---|---|---|---|---|---|---|---|---|---|
| Azerbaijan | 744 | 11.2 | 547 | 8.2 | 2 | 0.03 | 18 | 0.3 | 38 | 0.59 | 59 | 1.93 |
| Tunisia | 659 | 8.2 | 414 | 5.3 | 16 | 0.19 | 50 | 0.54 | 34 | 0.4 | 75 | 2.34 |
| Iraq | 653 | 4.7 | 311 | 2.2 | 19 | 0.14 | 28 | 0.22 | 18 | 0.12 | 17 | 2.85 |
| Lesotho | 642 | 65.1 | 598 | 60.5 | 3 | 0.34 | 27 | 2.8 | 5 | 0.53 | 127 | 1.31 |
| Finland | 641 | 10.7 | 179 | 4.6 | 23 | 0.33 | 140 | 1.7 | 33 | 0.53 | 95 | 1.54 |
| Saudi Arabia | 611 | 4.9 | 332 | 2.4 | 4 | 0.04 | 16 | 0.14 | 28 | 0.24 | 483 | 2.56 |
| Togo | 599 | 22.6 | 511 | 19.1 | 17 | 0.68 | 18 | 0.74 | 9 | 0.32 | 0 | 0 |
| Israel | 596 | 9.7 | 330 | 6.2 | 20 | 0.25 | 74 | 0.85 | 17 | 0.23 | 188 | 3.28 |
| Kyrgyzstan | 582 | 17.3 | 479 | 14.1 | 9 | 0.28 | 34 | 1 | 9 | 0.29 | 22 | 4.24 |
| Uruguay | 564 | 20.9 | 377 | 15.9 | 18 | 0.44 | 47 | 1.1 | 37 | 1.1 | 30 | 1.89 |
| Turkmenistan | 562 | 17.8 | 451 | 14.1 | 7 | 0.25 | 16 | 0.54 | 20 | 0.64 | 30 | 2.66 |
| Lao People's Democratic Republic | 552 | 17.2 | 401 | 12 | 8 | 0.27 | 19 | 0.61 | 7 | 0.24 | 1061 | 1.27 |
| Singapore | 548 | 11.0 | 353 | 7.4 | 22 | 0.38 | 38 | 0.63 | 26 | 0.47 | 44 | 1.06 |
| Croatia | 539 | 13.0 | 272 | 8.3 | 20 | 0.36 | 103 | 1.6 | 23 | 0.44 | 28 | 1.85 |
| Mauritania | 529 | 32.1 | 468 | 28.3 | 8 | 0.46 | 13 | 0.86 | 10 | 0.61 | 29 | 4.13 |
| Ireland | 527 | 13.9 | 242 | 7.5 | 13 | 0.24 | 73 | 1.6 | 40 | 0.88 | 431 | 1.87 |
| Botswana | 524 | 44.9 | 454 | 39.1 | 7 | 0.6 | 34 | 2.6 | 12 | 0.94 | 118 | 0.92 |
| Moldova | 518 | 16.5 | 420 | 14.2 | 3 | 0.07 | 43 | 0.95 | 8 | 0.24 | 1077 | 3.35 |
| Yemen | 496 | 5.4 | 212 | 2.1 | 36 | 0.41 | 18 | 0.24 | 7 | 0.06 | 54 | 5.65 |
| Sierra Leone | 488 | 16.6 | 486 | 16.5 | 0 | 0 | 0 | 0 | 1 | 0.02 | 409 | 2.79 |
| Georgia | 481 | 13.3 | 330 | 10.4 | 16 | 0.32 | 63 | 1.2 | 8 | 0.11 | 6 | 2.9 |
| Syrian Arab Republic | 472 | 5.8 | 206 | 2.5 | 14 | 0.17 | 45 | 0.56 | 13 | 0.17 | 153 | 3.06 |
| Congo, Republic of | 457 | 26.1 | 397 | 22.3 | 5 | 0.29 | 23 | 1.6 | 15 | 0.84 | 48 | 1.33 |
| Panama | 454 | 17.3 | 371 | 14.5 | 13 | 0.45 | 13 | 0.4 | 12 | 0.41 | 120 | 1.77 |
| Eswatini | 453 | 103.8 | 417 | 95.9 | 3 | 0.73 | 24 | 5.1 | 6 | 1.4 | 876 | 1.55 |
| Lithuania | 453 | 17.8 | 296 | 13.7 | 13 | 0.33 | 46 | 0.95 | 23 | 0.5 | 36 | 1.62 |
| Costa Rica | 451 | 13.2 | 341 | 10.6 | 10 | 0.24 | 30 | 0.67 | 10 | 0.24 | 273 | 4.69 |
| Namibia | 441 | 42.7 | 350 | 33.5 | 12 | 1.2 | 19 | 1.7 | 6 | 0.66 | 18 | 1.39 |
| New Zealand | 439 | 10.7 | 149 | 4.9 | 22 | 0.45 | 71 | 1.4 | 44 | 0.92 | 6066 | 7.25 |
| Jamaica | 426 | 22.8 | 376 | 20.4 | 6 | 0.31 | 7 | 0.37 | 10 | 0.46 | 45 | 1.51 |
| Mongolia | 407 | 23.2 | 361 | 20.2 | 10 | 0.63 | 6 | 0.42 | 2 | 0.14 | 471 | 13.75 |

| | | | | | | | | | | | | |
|---|---|---|---|---|---|---|---|---|---|---|---|---|
| Bosnia Herzegovina | 391 | 15.7 | 263 | 12.3 | 14 | 0.35 | 33 | 0.8 | 5 | 0.16 | 34 | 0.86 |
| Latvia | 387 | 22.8 | 238 | 16.9 | 13 | 0.44 | 53 | 1.6 | 19 | 0.85 | 502 | 2.35 |
| Libya | 366 | 10.8 | 278 | 8 | 9 | 0.28 | 14 | 0.45 | 6 | 0.16 | 1236 | 2.21 |
| Puerto Rico | 366 | 14.3 | 212 | 10.6 | 24 | 0.6 | 35 | 0.73 | 29 | 0.76 | 2040 | 4.78 |
| Tajikistan | 359 | 9.0 | 277 | 6.6 | 1 | 0.02 | 9 | 0.3 | 16 | 0.43 | 437 | 3.07 |
| Central African Republic | 342 | 25.2 | 295 | 21.8 | 3 | 0.21 | 16 | 1.3 | 9 | 0.63 | 66 | 1.62 |
| The Republic of the Gambia | 327 | 39.7 | 325 | 39.4 | 0 | 0 | 1 | 0.11 | 0 | 0 | 5 | 1.5 |
| Gabon | 306 | 37.2 | 271 | 32.5 | 4 | 0.46 | 2 | 0.24 | 6 | 0.71 | 701 | 1.27 |
| Lebanon | 292 | 7.3 | 144 | 3.6 | 12 | 0.27 | 46 | 1.1 | 10 | 0.25 | 650 | 3.03 |
| Albania | 287 | 12.7 | 171 | 8.7 | 17 | 0.62 | 30 | 1 | 4 | 0.21 | 4538 | 3.05 |
| Slovenia | 282 | 13.3 | 119 | 7.2 | 12 | 0.51 | 61 | 1.8 | 10 | 0.33 | 34 | 0.73 |
| Eritrea | 242 | 20.1 | 196 | 16.4 | 5 | 0.47 | 16 | 1.3 | 3 | 0.2 | 2 | 1.45 |
| Trinidad and Tobago | 237 | 22.2 | 192 | 18.3 | 6 | 0.52 | 8 | 0.61 | 7 | 0.52 | 0 | 0 |
| Guinea-Bissau | 231 | 35.6 | 224 | 34.3 | 1 | 0.21 | 1 | 0.18 | 0 | 0 | 0 | 0 |
| Jordan | 229 | 5.7 | 133 | 3.2 | 11 | 0.28 | 12 | 0.32 | 9 | 0.23 | 231 | 2.04 |
| Armenia | 215 | 8.7 | 156 | 6.8 | 8 | 0.28 | 17 | 0.48 | 6 | 0.21 | 117 | 2.02 |
| Estonia | 215 | 17.6 | 121 | 11.8 | 5 | 0.34 | 32 | 1.4 | 11 | 0.75 | 275 | 2.65 |
| Fiji | 194 | 41.1 | 165 | 35 | 2 | 0.46 | 0 | 0 | 5 | 1 | 1 | 0.03 |
| United Arab Emirates | 191 | 9.1 | 138 | 5.7 | 2 | 0.11 | 3 | 0.1 | 7 | 0.34 | 109 | 2.08 |
| Mauritius | 183 | 16.9 | 136 | 12.9 | 5 | 0.44 | 10 | 0.77 | 2 | 0.16 | 220 | 4 |
| Comoros | 169 | 53.6 | 163 | 52 | 0 | 0 | 6 | 1.6 | 0 | 0 | 80 | 3.46 |
| North Macedonia | 162 | 9.2 | 107 | 6.7 | 4 | 0.22 | 8 | 0.33 | 7 | 0.33 | 7 | 2.6 |
| Equatorial Guinea | 137 | 36.1 | 127 | 33.2 | 1 | 0.14 | 0 | 0 | 2 | 0.43 | 106 | 2.55 |
| Guyana | 135 | 31.6 | 129 | 30.3 | 0 | 0 | 3 | 0.67 | 1 | 0.25 | 879 | 2.94 |
| Oman | 117 | 8.5 | 90 | 6.4 | 4 | 0.38 | 1 | 0.05 | 4 | 0.26 | 68 | 1.96 |
| France, La Réunion | 110 | 15.3 | 75 | 11 | 0 | 0 | 9 | 0.99 | 9 | 1.1 | 2042 | 3.37 |
| Cyprus | 100 | 10.2 | 62 | 7 | 0 | 0 | 13 | 0.96 | 7 | 0.59 | 714 | 3.88 |
| Montenegro | 95 | 17.2 | 58 | 12 | 2 | 0.26 | 2 | 0.3 | 4 | 0.51 | 282 | 1.85 |
| Kuwait | 92 | 5.2 | 61 | 3.1 | 0 | 0 | 2 | 0.12 | 3 | 0.16 | 4 | 1.1 |
| Suriname | 92 | 27.3 | 81 | 24.2 | 1 | 0.21 | 4 | 1.2 | 2 | 0.57 | 460 | 3.96 |
| Gaza Strip and West Bank | 85 | 4.9 | 59 | 3.1 | 2 | 0.16 | 5 | 0.35 | 0 | 0 | 341 | 3.09 |

| | | | | | | | | | | | | |
|---|---|---|---|---|---|---|---|---|---|---|---|---|
| Timor-Leste | 83 | 16.0 | 77 | 14.8 | 0 | 0 | 0 | 0 | 0 | 0 | 194 | 2.38 |
| Djibouti | 82 | 19.2 | 71 | 16.5 | 1 | 0.22 | 3 | 0.86 | 0 | 0 | 56 | 1.6 |
| Solomon Islands | 82 | 29.8 | 72 | 26.2 | 1 | 0.35 | 0 | 0 | 2 | 0.67 | 287 | 1.65 |
| Bhutan | 61 | 17.6 | 49 | 13.6 | 0 | 0 | 0 | 0 | 0 | 0 | 2412 | 3.44 |
| Brunei Darussalam | 55 | 20.8 | 53 | 20 | 0 | 0 | 0 | 0 | 0 | 0 | 968 | 4.86 |
| Cape Verde | 55 | 19.3 | 46 | 16.1 | 0 | 0 | 1 | 0.32 | 1 | 0.32 | 1 | 0.14 |
| Luxembourg | 54 | 9.8 | 22 | 4.4 | 3 | 0.28 | 10 | 2 | 2 | 0.22 | 6 | 1.2 |
| Maldives | 51 | 22.6 | 51 | 22.6 | 0 | 0 | 0 | 0 | 0 | 0 | 44 | 1.71 |
| Barbados | 49 | 17.7 | 36 | 13.8 | 0 | 0 | 3 | 1.5 | 6 | 1.8 | 24 | 2.23 |
| Malta | 49 | 10.7 | 13 | 4.2 | 2 | 0.23 | 10 | 1.4 | 2 | 0.63 | 145 | 1.75 |
| Bahamas | 46 | 16.8 | 42 | 15.2 | 2 | 0.81 | 0 | 0 | 2 | 0.78 | 1113 | 1.88 |
| France, Guadeloupe | 46 | 12.4 | 32 | 9.9 | 0 | 0 | 5 | 0.66 | 3 | 0.6 | 68 | 2.22 |
| France, Martinique | 38 | 8.9 | 29 | 7.5 | 2 | 0.27 | 0 | 0 | 5 | 0.88 | 193 | 1.71 |
| Iceland | 38 | 14.0 | 19 | 9.4 | 1 | 0.27 | 8 | 1.9 | 3 | 1 | 965 | 1.94 |
| New Caledonia | 35 | 18.1 | 27 | 14.2 | 0 | 0 | 0 | 0 | 2 | 1 | 41 | 2.85 |
| Belize | 34 | 17.3 | 34 | 17.3 | 0 | 0 | 0 | 0 | 0 | 0 | 3405 | 5.14 |
| Qatar | 30 | 6.2 | 25 | 4.7 | 0 | 0 | 0 | 0 | 0 | 0 | 13563 | 4.34 |
| Vanuatu | 29 | 23.1 | 23 | 18.2 | 0 | 0 | 0 | 0 | 1 | 0.72 | 85 | 2.36 |
| Bahrain | 25 | 4.1 | 18 | 2.7 | 0 | 0 | 1 | 0.15 | 0 | 0 | 370 | 2.21 |
| Saint Lucia | 24 | 18.9 | 20 | 15.7 | 1 | 0.87 | 1 | 0.92 | 0 | 0 | 5 | 4.2 |
| French Guyana | 20 | 13.1 | 19 | 12.3 | 0 | 0 | 0 | 0 | 1 | 0.78 | 373 | 1.99 |
| French Polynesia | 20 | 10.7 | 15 | 8.3 | 0 | 0 | 0 | 0 | 1 | 0.41 | 971 | 1.42 |
| Guam | 20 | 19.8 | 19 | 19 | 0 | 0 | 0 | 0 | 0 | 0 | 223 | 2.58 |
| Sao Tome and Principe | 14 | 18.8 | 14 | 18.8 | 0 | 0 | 0 | 0 | 0 | 0 | 75 | 1.45 |
| Samoa | 11 | 13.3 | 11 | 13.3 | 0 | 0 | 0 | 0 | 0 | 0 | 69 | 1.44 |

**Supplementary Table S2: Estimated new cases and age-standardized mortality rates (ASMR) for HPV-related cancers by site and sex in 2022**

| Countries | Total, both sexes | | Total, males | | Anus, males | | Penis, males | | Head and neck, males | |
|---|---|---|---|---|---|---|---|---|---|---|
| | Cases | ASMR (World) | Cases | ASMR (World) | Cases | ASMR (World) | Cases | ASMR (World) | Cases | ASMR (World) |
| **World** | 755303 | 10.2 | 288540 | 6.1 | 10874 | 0.22 | 13738 | 0.28 | 263928 | 5.6 |
| **HDI** | | | | | | | | | | |
| Very high HDI country | 155038 | 6.4 | 70291 | 4.8 | 3147 | 0.2 | 3138 | 0.19 | 64006 | 4.4 |
| High HDI country | 225118 | 7.3 | 77807 | 3.9 | 3702 | 0.18 | 4348 | 0.22 | 69757 | 3.54 |
| Medium HDI country | 285948 | 17.7 | 121734 | 11.6 | 2156 | 0.21 | 5558 | 0.54 | 114020 | 10.8 |
| Low HDI country | 89199 | 17.1 | 18708 | 5.7 | 1869 | 0.58 | 694 | 0.2 | 16145 | 4.95 |
| **Continents** | | | | | | | | | | |
| Africa | 107220 | 16.5 | 15248 | 4.1 | 2181 | 0.55 | 894 | 0.21 | 12173 | 3.34 |
| Latin America and the Caribbean | 62809 | 9.9 | 21395 | 5.7 | 583 | 0.15 | 1674 | 0.43 | 19138 | 5.1 |
| Northern America | 24531 | 4.5 | 10886 | 3.1 | 759 | 0.23 | 461 | 0.12 | 9666 | 2.74 |
| Europe | 97760 | 8.4 | 48430 | 7.1 | 1819 | 0.24 | 2011 | 0.25 | 44600 | 6.6 |
| Oceania | 3418 | 7 | 1364 | 4.1 | 98 | 0.26 | 48 | 0.12 | 1218 | 3.72 |
| Asia | 459830 | 10.3 | 191299 | 6.7 | 5434 | 0.18 | 8650 | 0.29 | 177215 | 6.21 |
| **World Bank Classification** | | | | | | | | | | |
| High income | 102809 | 5.2 | 47787 | 3.9 | 2669 | 0.22 | 2260 | 0.16 | 42858 | 3.5 |
| Upper middle income | 222557 | 7.3 | 81777 | 4.3 | 3443 | 0.18 | 4516 | 0.23 | 73818 | 3.84 |
| Lower middle income | 373841 | 15.6 | 151287 | 9.9 | 4004 | 0.26 | 6366 | 0.42 | 140917 | 9.2 |
| Low income | 52462 | 16.9 | 6720 | 3.8 | 733 | 0.39 | 508 | 0.26 | 5479 | 3.12 |
| **Countries** | | | | | | | | | | |
| India | 207634 | 19.3 | 97402 | 13.5 | 1488 | 0.21 | 4450 | 0.63 | 91464 | 12.7 |
| China | 107748 | 5.6 | 37667 | 3.0 | 2293 | 0.18 | 2275 | 0.18 | 33099 | 2.63 |
| Indonesia | 29123 | 12.8 | 6014 | 4.5 | 259 | 0.21 | 419 | 0.3 | 5336 | 3.94 |
| Brazil | 25690 | 11.6 | 12151 | 9.2 | 310 | 0.23 | 547 | 0.4 | 11294 | 8.6 |
| Bangladesh | 22512 | 19.1 | 12257 | 15.7 | 148 | 0.19 | 244 | 0.31 | 11865 | 15.2 |
| United States of America | 21362 | 4.5 | 9486 | 3.1 | 692 | 0.24 | 389 | 0.12 | 8405 | 2.72 |

| | | | | | | | | | | |
|---|---|---|---|---|---|---|---|---|---|---|
| Russian Federation | 21171 | 11.3 | 9784 | 9.5 | 167 | 0.17 | 289 | 0.27 | 9328 | 9.1 |
| Pakistan | 17123 | 13.5 | 9814 | 11.4 | 193 | 0.23 | 93 | 0.11 | 9528 | 11.07 |
| Japan | 11338 | 3.6 | 4461 | 2.1 | 269 | 0.13 | 177 | 0.07 | 4015 | 1.9 |
| Nigeria | 10491 | 14.2 | 1816 | 3.8 | 696 | 1.4 | 13 | 0.01 | 1107 | 2.38 |
| Germany | 10194 | 6.3 | 4807 | 4.9 | 270 | 0.27 | 279 | 0.23 | 4258 | 4.4 |
| Thailand | 9037 | 9.3 | 2835 | 4.8 | 60 | 0.1 | 243 | 0.41 | 2532 | 4.31 |
| Tanzania, United Republic of | 8160 | 36.4 | 750 | 6.5 | 175 | 1.2 | 68 | 0.59 | 507 | 4.75 |
| South Africa | 8041 | 18.7 | 1245 | 5.4 | 110 | 0.39 | 96 | 0.37 | 1039 | 4.68 |
| Ethiopia | 7805 | 15 | 818 | 2.4 | 116 | 0.32 | 20 | 0.05 | 682 | 2.02 |
| Poland | 7722 | 13 | 3934 | 11.7 | 126 | 0.33 | 191 | 0.51 | 3617 | 10.9 |
| Congo, Democratic Republic of | 7612 | 21 | 706 | 3.3 | 181 | 0.82 | 70 | 0.32 | 455 | 2.15 |
| Myanmar | 7367 | 16.6 | 2218 | 9.2 | 39 | 0.19 | 177 | 0.85 | 2002 | 8.2 |
| Ukraine | 7289 | 12.9 | 3778 | 12.1 | 64 | 0.2 | 86 | 0.26 | 3628 | 11.6 |
| Philippines | 7268 | 9.5 | 2010 | 4.6 | 61 | 0.14 | 53 | 0.12 | 1896 | 4.33 |
| Mexico | 7134 | 6.3 | 1439 | 2.1 | 43 | 0.06 | 241 | 0.35 | 1155 | 1.67 |
| Italy | 6838 | 5.1 | 3345 | 4.3 | 177 | 0.22 | 162 | 0.17 | 3006 | 3.95 |
| France (metropolitan) | 6517 | 5.9 | 3184 | 4.8 | 158 | 0.23 | 132 | 0.16 | 2894 | 4.4 |
| United Kingdom | 6379 | 5.7 | 3042 | 4.3 | 224 | 0.3 | 194 | 0.25 | 2624 | 3.74 |
| Viet Nam | 5999 | 6.4 | 2580 | 4.8 | 126 | 0.23 | 152 | 0.28 | 2302 | 4.25 |
| Uganda | 5660 | 35.2 | 602 | 7.8 | 55 | 0.75 | 146 | 1.5 | 401 | 5.53 |
| Romania | 4960 | 17.7 | 2605 | 15.7 | 82 | 0.43 | 55 | 0.3 | 2468 | 15 |
| Mozambique | 4634 | 31.6 | 288 | 3.9 | 12 | 0.13 | 67 | 0.86 | 209 | 2.89 |
| Kenya | 4628 | 20.3 | 530 | 4.5 | 46 | 0.45 | 22 | 0.17 | 462 | 3.89 |
| Argentina | 4581 | 9.9 | 1399 | 5.0 | 41 | 0.15 | 160 | 0.56 | 1198 | 4.3 |
| Spain | 4577 | 5.2 | 2520 | 4.8 | 88 | 0.18 | 131 | 0.22 | 2301 | 4.4 |
| Colombia | 4033 | 7.8 | 972 | 3.1 | 35 | 0.11 | 154 | 0.5 | 783 | 2.45 |
| Türkiye | 3775 | 4.4 | 1976 | 4.0 | 35 | 0.07 | 6 | 0.01 | 1935 | 3.93 |
| Malawi | 3740 | 43 | 176 | 3.9 | 16 | 0.36 | 63 | 1.2 | 97 | 2.33 |
| Madagascar | 3444 | 26.8 | 439 | 5.9 | 70 | 0.95 | 4 | 0.04 | 365 | 4.89 |
| Venezuela | 3431 | 13.3 | 864 | 5.6 | 25 | 0.16 | 85 | 0.55 | 754 | 4.85 |
| Peru | 3311 | 10.4 | 410 | 1.9 | 21 | 0.1 | 88 | 0.42 | 301 | 1.42 |

| | | | | | | | | | | |
|---|---|---|---|---|---|---|---|---|---|---|
| Iran, Islamic Republic of | 3298 | 4.9 | 1744 | 3.8 | 55 | 0.12 | 21 | 0.05 | 1668 | 3.67 |
| Egypt | 3170 | 4.8 | 1554 | 4.0 | 107 | 0.26 | 4 | 0.01 | 1443 | 3.72 |
| Canada | 3169 | 4.8 | 1400 | 3.2 | 67 | 0.18 | 72 | 0.15 | 1261 | 2.9 |
| Sri Lanka | 2980 | 12.1 | 1620 | 11.3 | 34 | 0.22 | 43 | 0.29 | 1543 | 10.8 |
| Morocco | 2972 | 9.1 | 1056 | 5.1 | 69 | 0.34 | 6 | 0.03 | 981 | 4.72 |
| Zimbabwe | 2667 | 42 | 169 | 5.6 | 22 | 0.72 | 49 | 1.4 | 98 | 3.44 |
| Zambia | 2625 | 41.3 | 169 | 4.6 | 6 | 0.14 | 67 | 1.6 | 96 | 2.89 |
| Republic of Korea | 2580 | 2.9 | 1037 | 1.9 | 42 | 0.08 | 23 | 0.04 | 972 | 1.78 |
| Cuba | 2536 | 14.6 | 1493 | 13.5 | 9 | 0.09 | 43 | 0.35 | 1441 | 13.1 |
| Angola | 2507 | 21.2 | 454 | 6.8 | 14 | 0.19 | 31 | 0.58 | 409 | 6 |
| Uzbekistan | 2418 | 10 | 515 | 3.7 | 45 | 0.31 | 4 | 0.04 | 466 | 3.36 |
| Ghana | 2327 | 15 | 285 | 3.0 | 41 | 0.32 | 6 | 0.06 | 238 | 2.65 |
| Nepal | 2282 | 11.2 | 698 | 5.9 | 12 | 0.09 | 89 | 0.76 | 597 | 5.06 |
| Cameroon | 2279 | 22.3 | 256 | 3.9 | 35 | 0.46 | 5 | 0.06 | 216 | 3.42 |
| Malaysia | 2037 | 7.3 | 689 | 3.8 | 12 | 0.07 | 23 | 0.12 | 654 | 3.6 |
| Afghanistan | 1984 | 12.1 | 743 | 7.2 | 38 | 0.36 | 7 | 0.05 | 698 | 6.79 |
| Algeria | 1971 | 5.8 | 767 | 3.4 | 43 | 0.18 | 2 | 0.01 | 722 | 3.19 |
| Hungary | 1934 | 13.1 | 1041 | 12.6 | 12 | 0.13 | 27 | 0.29 | 1002 | 12.2 |
| Korea, Democratic People Republic of | 1932 | 7 | 502 | 3.0 | 10 | 0.06 | 23 | 0.15 | 469 | 2.8 |
| Guinea | 1845 | 32.5 | 70 | 2.4 | 14 | 0.44 | 0 | 0 | 56 | 1.97 |
| Côte d'Ivoire | 1838 | 16.5 | 193 | 2.5 | 24 | 0.28 | 2 | 0.03 | 167 | 2.2 |
| Kazakhstan | 1819 | 10.6 | 654 | 7.0 | 21 | 0.22 | 17 | 0.19 | 616 | 6.6 |
| Mali | 1790 | 23 | 151 | 3.3 | 25 | 0.5 | 3 | 0.04 | 123 | 2.79 |
| Portugal | 1740 | 9.3 | 975 | 9.5 | 23 | 0.18 | 32 | 0.21 | 920 | 9.1 |
| Belarus | 1737 | 13.9 | 1119 | 16.5 | 20 | 0.28 | 18 | 0.24 | 1081 | 16 |
| Senegal | 1582 | 20.9 | 113 | 2.7 | 25 | 0.57 | 2 | 0.03 | 86 | 2.1 |
| Australia | 1549 | 3.9 | 781 | 3.1 | 57 | 0.21 | 31 | 0.11 | 693 | 2.74 |
| Czechia | 1546 | 8.9 | 766 | 7.5 | 80 | 0.71 | 43 | 0.36 | 643 | 6.4 |
| Serbia | 1471 | 11.4 | 741 | 9.2 | 28 | 0.3 | 28 | 0.32 | 685 | 8.6 |
| Chile | 1378 | 5.6 | 346 | 2.3 | 19 | 0.13 | 43 | 0.27 | 284 | 1.86 |
| The Netherlands | 1374 | 4.3 | 590 | 2.9 | 46 | 0.22 | 44 | 0.18 | 500 | 2.46 |

| | | | | | | | | | | |
|---|---|---|---|---|---|---|---|---|---|---|
| Sudan | 1367 | 6.6 | 370 | 2.9 | 0 | 0 | 10 | 0.09 | 360 | 2.76 |
| Bolivia | 1359 | 14.5 | 156 | 2.2 | 0 | 0 | 61 | 0.72 | 95 | 1.48 |
| Burundi | 1328 | 31 | 151 | 6.6 | 21 | 0.88 | 32 | 1.3 | 98 | 4.38 |
| Ecuador | 1290 | 8.3 | 206 | 2.1 | 10 | 0.1 | 45 | 0.46 | 151 | 1.58 |
| Cambodia | 1276 | 11.8 | 364 | 6.4 | 6 | 0.1 | 47 | 0.73 | 311 | 5.57 |
| Papua New Guinea | 1258 | 26.3 | 393 | 14.3 | 26 | 1.3 | 13 | 0.65 | 354 | 12.3 |
| Somalia | 1235 | 20.1 | 162 | 4.3 | 15 | 0.38 | 2 | 0.04 | 145 | 3.89 |
| Guatemala | 1158 | 10.6 | 103 | 1.6 | 0 | 0 | 7 | 0.11 | 96 | 1.51 |
| Bulgaria | 1157 | 10.9 | 567 | 8.9 | 13 | 0.19 | 24 | 0.32 | 530 | 8.4 |
| Burkina Faso | 1147 | 13.6 | 167 | 3.1 | 13 | 0.12 | 0 | 0 | 154 | 3.01 |
| Greece | 1105 | 5.2 | 587 | 4.4 | 28 | 0.23 | 43 | 0.28 | 516 | 3.9 |
| Slovakia | 1058 | 13.7 | 602 | 13.2 | 42 | 0.83 | 14 | 0.29 | 546 | 12.1 |
| Dominican Republic | 1035 | 11.1 | 291 | 4.9 | 3 | 0.05 | 19 | 0.31 | 269 | 4.54 |
| Chad | 1033 | 15.2 | 58 | 1.2 | 7 | 0.22 | 0 | 0 | 51 | 1.01 |
| Belgium | 996 | 5.1 | 484 | 4.2 | 31 | 0.21 | 21 | 0.15 | 432 | 3.8 |
| Austria | 961 | 6.1 | 505 | 5.4 | 20 | 0.19 | 26 | 0.2 | 459 | 5 |
| Honduras | 905 | 14.7 | 173 | 4.6 | 8 | 0.19 | 36 | 0.91 | 129 | 3.46 |
| Paraguay | 856 | 16 | 206 | 6.1 | 5 | 0.14 | 36 | 1.1 | 165 | 4.9 |
| Sweden | 838 | 4.2 | 300 | 2.4 | 16 | 0.14 | 37 | 0.25 | 247 | 1.97 |
| Iraq | 828 | 4.6 | 405 | 4.0 | 16 | 0.16 | 2 | 0.01 | 387 | 3.8 |
| Rwanda | 825 | 13.3 | 141 | 4.0 | 2 | 0.05 | 41 | 1 | 98 | 2.96 |
| South Sudan | 800 | 16.4 | 118 | 3.9 | 11 | 0.35 | 13 | 0.4 | 94 | 3.17 |
| Niger | 758 | 8.3 | 188 | 3.2 | 70 | 1.1 | 1 | 0.02 | 117 | 2.12 |
| Moldova | 753 | 15.8 | 470 | 17.3 | 2 | 0.07 | 7 | 0.26 | 461 | 17 |
| Azerbaijan | 749 | 7.9 | 313 | 5.6 | 45 | 0.79 | 1 | 0.02 | 267 | 4.79 |
| Tunisia | 728 | 6.1 | 392 | 5.4 | 26 | 0.35 | 1 | 0.01 | 365 | 5.01 |
| Haiti | 714 | 9.7 | 175 | 4.1 | 35 | 0.87 | 23 | 0.51 | 117 | 2.74 |
| Yemen | 708 | 5.9 | 348 | 5.2 | 12 | 0.16 | 0 | 0 | 336 | 5.07 |
| Georgia | 655 | 12 | 353 | 11.9 | 6 | 0.2 | 14 | 0.44 | 333 | 11.3 |
| Switzerland | 639 | 3.9 | 324 | 3.3 | 21 | 0.18 | 11 | 0.09 | 292 | 2.99 |
| Benin | 624 | 11.7 | 79 | 2.5 | 26 | 0.71 | 3 | 0.13 | 50 | 1.65 |

| | | | | | | | | | | |
|---|---|---|---|---|---|---|---|---|---|---|
| Croatia | 611 | 8.3 | 334 | 8.1 | 7 | 0.13 | 11 | 0.21 | 316 | 7.8 |
| Lithuania | 611 | 14.7 | 331 | 14.8 | 6 | 0.2 | 9 | 0.3 | 316 | 14.3 |
| Syrian Arab Republic | 578 | 5.1 | 304 | 4.4 | 5 | 0.07 | 3 | 0.05 | 296 | 4.29 |
| Denmark | 572 | 5.5 | 271 | 4.2 | 22 | 0.29 | 19 | 0.24 | 230 | 3.67 |
| Saudi Arabia | 558 | 2.7 | 268 | 1.6 | 27 | 0.15 | 2 | 0.01 | 239 | 1.47 |
| Liberia | 555 | 22.8 | 42 | 2.7 | 8 | 0.49 | 1 | 0.03 | 33 | 2.13 |
| El Salvador | 499 | 8.3 | 69 | 2.0 | 0 | 0 | 8 | 0.23 | 61 | 1.78 |
| Nicaragua | 497 | 10.4 | 96 | 3.7 | 1 | 0.04 | 20 | 0.74 | 75 | 2.89 |
| Togo | 489 | 13.5 | 97 | 4.4 | 23 | 0.88 | 1 | 0.06 | 73 | 3.41 |
| Turkmenistan | 488 | 11.4 | 137 | 5.7 | 10 | 0.42 | 0 | 0 | 127 | 5.3 |
| Lesotho | 485 | 36.5 | 38 | 5.9 | 1 | 0.14 | 10 | 1.5 | 27 | 4.22 |
| Uruguay | 455 | 9.5 | 214 | 8.1 | 6 | 0.21 | 14 | 0.46 | 194 | 7.38 |
| Kyrgyzstan | 445 | 10 | 108 | 4.8 | 3 | 0.11 | 1 | 0.03 | 104 | 4.62 |
| Lao People's Democratic Republic | 427 | 9.8 | 133 | 5.0 | 2 | 0.07 | 17 | 0.62 | 114 | 4.26 |
| Bosnia Herzegovina | 413 | 8.1 | 202 | 6.2 | 1 | 0.03 | 3 | 0.09 | 198 | 6.11 |
| Singapore | 404 | 4.6 | 180 | 3.1 | 10 | 0.17 | 6 | 0.1 | 164 | 2.8 |
| Latvia | 396 | 13 | 192 | 12.1 | 5 | 0.24 | 7 | 0.37 | 180 | 11.5 |
| Ireland | 392 | 5.7 | 205 | 4.6 | 10 | 0.2 | 15 | 0.3 | 180 | 4.1 |
| Mauritania | 386 | 17.6 | 43 | 3.3 | 13 | 0.85 | 0 | 0 | 30 | 2.41 |
| Norway | 363 | 3.6 | 126 | 2.1 | 11 | 0.17 | 12 | 0.2 | 103 | 1.74 |
| Finland | 362 | 3.4 | 172 | 2.6 | 10 | 0.11 | 13 | 0.17 | 149 | 2.32 |
| Botswana | 352 | 23.7 | 64 | 9.1 | 2 | 0.2 | 14 | 1.8 | 48 | 7.08 |
| Libya | 352 | 7.8 | 136 | 5.3 | 5 | 0.14 | 0 | 0 | 131 | 5.16 |
| Israel | 349 | 3.3 | 116 | 1.8 | 5 | 0.09 | 2 | 0.03 | 109 | 1.64 |
| Jamaica | 322 | 11.4 | 68 | 3.5 | 5 | 0.27 | 7 | 0.32 | 56 | 2.95 |
| Namibia | 314 | 24.9 | 65 | 11.2 | 3 | 0.46 | 5 | 0.61 | 57 | 10.1 |
| Costa Rica | 313 | 5.5 | 107 | 2.9 | 3 | 0.08 | 11 | 0.27 | 93 | 2.51 |
| Eswatini | 311 | 52.1 | 17 | 6.0 | 1 | 0.28 | 10 | 3.4 | 6 | 2.34 |
| Panama | 307 | 7.3 | 78 | 2.8 | 2 | 0.08 | 10 | 0.36 | 66 | 2.36 |
| Central African Republic | 305 | 16.7 | 31 | 2.6 | 5 | 0.43 | 0 | 0 | 26 | 2.2 |
| Puerto Rico | 305 | 6.1 | 159 | 5.3 | 0 | 0 | 10 | 0.32 | 149 | 5 |

| | | | | | | | | | | |
|---|---|---|---|---|---|---|---|---|---|---|
| Congo, Republic of | 300 | 12.3 | 23 | 1.5 | 11 | 0.59 | 0 | 0 | 12 | 0.9 |
| New Zealand | 298 | 3.9 | 135 | 2.8 | 15 | 0.35 | 2 | 0.04 | 118 | 2.45 |
| Sierra Leone | 297 | 7.6 | 3 | 0.1 | 2 | 0.06 | 0 | 0 | 1 | 0.04 |
| Albania | 294 | 7.5 | 143 | 5.6 | 3 | 0.08 | 7 | 0.23 | 133 | 5.28 |
| Jordan | 287 | 5.2 | 159 | 4.5 | 5 | 0.13 | 0 | 0 | 154 | 4.33 |
| Lebanon | 282 | 4.7 | 107 | 2.8 | 2 | 0.06 | 4 | 0.1 | 101 | 2.62 |
| Tajikistan | 278 | 5.4 | 61 | 2.2 | 14 | 0.48 | 0 | 0 | 47 | 1.69 |
| Armenia | 250 | 6.9 | 119 | 6.0 | 2 | 0.1 | 6 | 0.27 | 111 | 5.67 |
| Mongolia | 241 | 11.3 | 54 | 4.9 | 2 | 0.16 | 3 | 0.22 | 49 | 4.5 |
| Slovenia | 238 | 6.3 | 120 | 5.5 | 1 | 0.04 | 6 | 0.21 | 113 | 5.2 |
| The Republic of the Gambia | 217 | 20.2 | 12 | 1.9 | 0 | 0 | 1 | 0.16 | 11 | 1.71 |
| Eritrea | 204 | 12.1 | 25 | 2.4 | 0 | 0 | 0 | 0 | 25 | 2.4 |
| Gabon | 199 | 17.6 | 45 | 6.5 | 7 | 0.82 | 0 | 0 | 38 | 5.71 |
| North Macedonia | 193 | 6.9 | 107 | 6.0 | 0 | 0 | 7 | 0.33 | 100 | 5.69 |
| Estonia | 182 | 8.1 | 92 | 8.0 | 3 | 0.27 | 5 | 0.32 | 84 | 7.4 |
| Trinidad and Tobago | 182 | 11.2 | 44 | 4.2 | 2 | 0.23 | 1 | 0.1 | 41 | 3.83 |
| Guinea-Bissau | 170 | 21 | 8 | 2.0 | 2 | 0.46 | 0 | 0 | 6 | 1.51 |
| United Arab Emirates | 148 | 4.2 | 57 | 2.1 | 3 | 0.16 | 1 | 0.06 | 53 | 1.88 |
| Mauritius | 141 | 8.5 | 52 | 5.2 | 0 | 0 | 0 | 0 | 52 | 5.2 |
| Oman | 130 | 5.4 | 63 | 3.1 | 13 | 0.67 | 0 | 0 | 50 | 2.4 |
| Fiji | 119 | 17.4 | 9 | 2.0 | 0 | 0 | 0 | 0 | 9 | 2.02 |
| Gaza Strip and West Bank | 118 | 5.3 | 67 | 4.8 | 2 | 0.18 | 0 | 0 | 65 | 4.64 |
| Comoros | 114 | 27.4 | 8 | 3.2 | 0 | 0 | 1 | 0.34 | 7 | 2.9 |
| France, La Réunion | 110 | 9.7 | 60 | 8.9 | 0 | 0 | 2 | 0.22 | 58 | 8.7 |
| Equatorial Guinea | 103 | 19.2 | 20 | 5.7 | 5 | 1 | 0 | 0 | 15 | 4.71 |
| Kuwait | 95 | 3.4 | 48 | 2.1 | 2 | 0.04 | 0 | 0 | 46 | 2.05 |
| Montenegro | 88 | 10 | 39 | 7.0 | 1 | 0.14 | 1 | 0.17 | 37 | 6.66 |
| Guyana | 80 | 12.5 | 13 | 3.3 | 0 | 0 | 1 | 0.26 | 12 | 3 |
| Solomon Islands | 70 | 17.8 | 14 | 5.8 | 0 | 0 | 2 | 0.72 | 12 | 5.09 |
| Djibouti | 68 | 11 | 9 | 2.4 | 0 | 0 | 0 | 0 | 9 | 2.4 |
| Cyprus | 66 | 3.5 | 24 | 2.3 | 1 | 0.11 | 2 | 0.22 | 21 | 1.94 |

| | | | | | | | | | | |
|---|---|---|---|---|---|---|---|---|---|---|
| Bhutan | 58 | 11.1 | 21 | 5.9 | 0 | 0 | 2 | 0.52 | 19 | 5.4 |
| Suriname | 56 | 11.2 | 11 | 3.8 | 0 | 0 | 2 | 0.79 | 9 | 2.98 |
| Timor-Leste | 53 | 7.6 | 10 | 2.3 | 0 | 0 | 0 | 0 | 10 | 2.26 |
| Bahamas | 47 | 12.1 | 14 | 6.2 | 0 | 0 | 1 | 0.35 | 13 | 5.8 |
| Barbados | 44 | 9.5 | 18 | 6.2 | 0 | 0 | 0 | 0 | 18 | 6.2 |
| France, Guadeloupe | 44 | 7.4 | 23 | 7.1 | 0 | 0 | 0 | 0 | 23 | 7.1 |
| Cape Verde | 42 | 10.8 | 10 | 4.6 | 0 | 0 | 0 | 0 | 10 | 4.56 |
| France, Martinique | 34 | 4.3 | 17 | 3.7 | 0 | 0 | 1 | 0.11 | 16 | 3.57 |
| Malta | 34 | 3.6 | 16 | 2.9 | 0 | 0 | 0 | 0 | 16 | 2.85 |
| Qatar | 33 | 3.1 | 18 | 1.3 | 1 | 0.11 | 0 | 0 | 17 | 1.19 |
| Belize | 29 | 10.4 | 5 | 2.5 | 0 | 0 | 0 | 0 | 5 | 2.5 |
| Vanuatu | 27 | 13.9 | 4 | 3.2 | 0 | 0 | 0 | 0 | 4 | 3.2 |
| Maldives | 26 | 7.7 | 0 | 0.0 | 0 | 0 | 0 | 0 | 0 | 0 |
| New Caledonia | 26 | 8.8 | 9 | 4.9 | 0 | 0 | 0 | 0 | 9 | 4.9 |
| Bahrain | 24 | 3 | 13 | 2.3 | 1 | 0.17 | 0 | 0 | 12 | 2.11 |
| Luxembourg | 24 | 2.6 | 11 | 1.9 | 0 | 0 | 0 | 0 | 11 | 1.87 |
| French Polynesia | 22 | 7.4 | 14 | 7.0 | 0 | 0 | 0 | 0 | 14 | 6.99 |
| Brunei Darussalam | 16 | 4.4 | 0 | 0.0 | 0 | 0 | 0 | 0 | 0 | 0 |
| Saint Lucia | 16 | 8.7 | 3 | 2.5 | 0 | 0 | 0 | 0 | 3 | 2.52 |
| French Guyana | 15 | 7 | 5 | 3.7 | 0 | 0 | 0 | 0 | 5 | 3.74 |
| Guam | 14 | 7.9 | 1 | 0.9 | 0 | 0 | 0 | 0 | 1 | 0.92 |
| Iceland | 14 | 2.9 | 5 | 1.6 | 0 | 0 | 0 | 0 | 5 | 1.58 |
| Sao Tome and Principe | 12 | 11.7 | 2 | 3.3 | 0 | 0 | 0 | 0 | 2 | 3.3 |
| Samoa | 7 | 5.6 | 1 | 1.3 | 0 | 0 | 0 | 0 | 1 | 1.3 |

**Supplementary Table S2: Estimated new cases and age-standardized mortality rates (ASMR) for HPV-related cancers by site and sex in 2022 (Continued)**

| Countries | Total, females | | Cervix uteri, females | | Vagina, females | | Vulva, females | | Anus, females | | Head and neck, females | |
|---|---|---|---|---|---|---|---|---|---|---|---|---|
| | Cases | ASMR (World) | Cases | ASMR (World) | Cases | ASMR (World) | Cases | ASMR (World) | Cases | ASMR (World) | Cases | ASMR (World) |
| **World** | 466763 | 9.3 | 348709 | 7.1 | 8239 | 0.15 | 18580 | 0.3 | 11161 | 0.2 | 80074 | 1.51 |
| **HDI** | | | | | | | | | | | | |
| Very high HDI country | 84747 | 5.0 | 48363 | 3.3 | 2238 | 0.1 | 9307 | 0.37 | 4311 | 0.21 | 20528 | 1 |
| High HDI country | 147311 | 7.1 | 118418 | 5.9 | 2021 | 0.09 | 4573 | 0.19 | 3402 | 0.15 | 18897 | 0.8 |
| Medium HDI country | 164214 | 15.0 | 123222 | 11.2 | 3086 | 0.28 | 2541 | 0.23 | 1928 | 0.18 | 33437 | 3.06 |
| Low HDI country | 70491 | 19.6 | 58706 | 16.3 | 894 | 0.25 | 2159 | 0.57 | 1520 | 0.42 | 7212 | 2.06 |
| **Continents** | | | | | | | | | | | | |
| Africa | 91972 | 20.1 | 80614 | 17.6 | 1119 | 0.24 | 2994 | 0.63 | 2100 | 0.46 | 5145 | 1.15 |
| Latin America and the Caribbean | 41414 | 9.3 | 33514 | 7.7 | 573 | 0.12 | 1359 | 0.25 | 980 | 0.2 | 4988 | 1 |
| Northern America | 13645 | 3.7 | 6692 | 2.2 | 471 | 0.1 | 1876 | 0.37 | 1142 | 0.27 | 3464 | 0.78 |
| Europe | 49330 | 6.0 | 26950 | 3.9 | 1341 | 0.12 | 6734 | 0.5 | 2535 | 0.24 | 11770 | 1.2 |
| Oceania | 2054 | 6.4 | 1309 | 4.5 | 60 | 0.17 | 131 | 0.27 | 113 | 0.29 | 441 | 1.19 |
| Asia | 268531 | 8.9 | 199795 | 6.7 | 4676 | 0.15 | 5485 | 0.16 | 4291 | 0.13 | 54284 | 1.79 |
| **World Bank Classification** | | | | | | | | | | | | |
| High income | 55022 | 4.0 | 26800 | 2.4 | 1730 | 0.09 | 7426 | 0.36 | 3722 | 0.23 | 15344 | 0.94 |
| Upper middle income | 140780 | 6.8 | 110230 | 5.5 | 2115 | 0.09 | 5318 | 0.22 | 3428 | 0.15 | 19689 | 0.82 |
| Lower middle income | 222554 | 13.6 | 169019 | 10.3 | 3773 | 0.23 | 4474 | 0.27 | 3066 | 0.19 | 42222 | 2.57 |
| Low income | 45742 | 21.0 | 40335 | 18.5 | 583 | 0.27 | 1320 | 0.58 | 880 | 0.4 | 2624 | 1.21 |
| **Countries** | | | | | | | | | | | | |
| India | 110232 | 15.5 | 79906 | 11.2 | 2465 | 0.35 | 1539 | 0.21 | 1138 | 0.16 | 25184 | 3.59 |
| China | 70081 | 5.5 | 55694 | 4.5 | 1057 | 0.08 | 1956 | 0.14 | 1866 | 0.13 | 9508 | 0.63 |
| Indonesia | 23109 | 14.7 | 20708 | 13.2 | 189 | 0.13 | 470 | 0.3 | 115 | 0.07 | 1627 | 1.04 |
| Brazil | 13539 | 8.6 | 9905 | 6.5 | 167 | 0.09 | 545 | 0.29 | 510 | 0.3 | 2412 | 1.38 |
| United States of America | 11876 | 3.7 | 5932 | 2.2 | 404 | 0.1 | 1569 | 0.36 | 998 | 0.27 | 2973 | 0.78 |
| Russian Federation | 11387 | 8.5 | 7903 | 6.4 | 227 | 0.13 | 1046 | 0.51 | 223 | 0.13 | 1988 | 1.28 |
| Bangladesh | 10255 | 12.8 | 5826 | 7 | 108 | 0.14 | 139 | 0.18 | 68 | 0.09 | 4114 | 5.38 |

| | | | | | | | | | | | | |
|---|---|---|---|---|---|---|---|---|---|---|---|---|
| Nigeria | 8675 | 17.2 | 7093 | 14.3 | 95 | 0.16 | 574 | 0.96 | 349 | 0.69 | 564 | 1.05 |
| Tanzania, United Republic of | 7410 | 45.6 | 6832 | 42.2 | 72 | 0.45 | 137 | 0.67 | 178 | 1.1 | 191 | 1.16 |
| Pakistan | 7309 | 8.7 | 3069 | 3.6 | 121 | 0.14 | 128 | 0.16 | 86 | 0.1 | 3905 | 4.74 |
| Ethiopia | 6987 | 19.4 | 5975 | 16.8 | 137 | 0.39 | 274 | 0.7 | 133 | 0.32 | 468 | 1.2 |
| Congo, Democratic Republic of | 6906 | 27.4 | 6187 | 24.6 | 75 | 0.3 | 218 | 0.79 | 213 | 0.84 | 213 | 0.83 |
| Japan | 6877 | 3.4 | 3864 | 2.6 | 170 | 0.06 | 328 | 0.09 | 270 | 0.08 | 2245 | 0.57 |
| South Africa | 6796 | 21.7 | 5976 | 19 | 69 | 0.23 | 267 | 0.82 | 114 | 0.35 | 370 | 1.25 |
| Thailand | 6202 | 9.0 | 4576 | 6.9 | 79 | 0.12 | 88 | 0.12 | 66 | 0.08 | 1393 | 1.79 |
| Mexico | 5695 | 7.1 | 4909 | 6.2 | 72 | 0.09 | 186 | 0.21 | 51 | 0.06 | 477 | 0.54 |
| Germany | 5387 | 4.7 | 2071 | 2.3 | 215 | 0.13 | 1190 | 0.71 | 430 | 0.34 | 1481 | 1.19 |
| Philippines | 5258 | 9.6 | 4380 | 8 | 61 | 0.11 | 76 | 0.14 | 36 | 0.06 | 705 | 1.25 |
| Myanmar | 5149 | 15.9 | 4374 | 13.4 | 38 | 0.12 | 55 | 0.17 | 32 | 0.1 | 650 | 2.08 |
| Uganda | 5058 | 43.1 | 4782 | 40.6 | 23 | 0.22 | 95 | 0.71 | 25 | 0.22 | 133 | 1.31 |
| Mozambique | 4346 | 40.0 | 4000 | 36.9 | 26 | 0.22 | 158 | 1.4 | 83 | 0.8 | 79 | 0.64 |
| Kenya | 4098 | 24.8 | 3591 | 21.4 | 32 | 0.21 | 82 | 0.56 | 126 | 0.8 | 267 | 1.86 |
| Poland | 3788 | 8.2 | 2188 | 5.2 | 83 | 0.14 | 443 | 0.67 | 134 | 0.23 | 940 | 2 |
| Malawi | 3564 | 57.7 | 3340 | 54.1 | 50 | 0.92 | 59 | 0.78 | 52 | 0.87 | 63 | 1.02 |
| Ukraine | 3511 | 8.6 | 2598 | 7 | 78 | 0.15 | 347 | 0.51 | 95 | 0.19 | 393 | 0.79 |
| Italy | 3493 | 3.5 | 1156 | 1.6 | 122 | 0.09 | 684 | 0.46 | 249 | 0.24 | 1282 | 1.12 |
| Viet Nam | 3419 | 4.9 | 2571 | 3.8 | 32 | 0.04 | 73 | 0.1 | 161 | 0.22 | 582 | 0.77 |
| United Kingdom | 3337 | 4.4 | 1154 | 2 | 129 | 0.14 | 579 | 0.49 | 414 | 0.49 | 1061 | 1.23 |
| France (metropolitan) | 3333 | 4.2 | 1530 | 2.3 | 83 | 0.07 | 385 | 0.28 | 345 | 0.39 | 990 | 1.17 |
| Argentina | 3182 | 9.9 | 2559 | 8.4 | 45 | 0.11 | 148 | 0.32 | 64 | 0.18 | 366 | 0.91 |
| Colombia | 3061 | 8.4 | 2435 | 6.9 | 57 | 0.14 | 114 | 0.27 | 82 | 0.21 | 373 | 0.92 |
| Madagascar | 3005 | 33.4 | 2690 | 30 | 34 | 0.38 | 116 | 1.2 | 84 | 0.97 | 81 | 0.89 |
| Peru | 2901 | 13.6 | 2545 | 12.1 | 32 | 0.13 | 77 | 0.3 | 46 | 0.2 | 201 | 0.9 |
| Venezuela | 2567 | 14.1 | 2246 | 12.5 | 36 | 0.19 | 35 | 0.17 | 61 | 0.32 | 189 | 0.96 |
| Zimbabwe | 2498 | 51.4 | 2318 | 47.9 | 26 | 0.51 | 80 | 1.5 | 33 | 0.63 | 41 | 0.88 |
| Zambia | 2456 | 52.7 | 2285 | 49.4 | 26 | 0.57 | 64 | 1.2 | 37 | 0.57 | 44 | 0.96 |
| Romania | 2355 | 11.5 | 1793 | 9.3 | 31 | 0.12 | 193 | 0.62 | 60 | 0.23 | 278 | 1.21 |
| Spain | 2057 | 3.2 | 802 | 1.6 | 75 | 0.08 | 377 | 0.37 | 77 | 0.1 | 726 | 1.02 |

| | | | | | | | | | | | | |
|---|---|---|---|---|---|---|---|---|---|---|---|---|
| Angola | 2053 | 24.2 | 1715 | 20.2 | 41 | 0.35 | 52 | 0.67 | 9 | 0.11 | 236 | 2.84 |
| Ghana | 2042 | 19.1 | 1815 | 16.9 | 9 | 0.07 | 35 | 0.39 | 92 | 0.87 | 91 | 0.85 |
| Cameroon | 2023 | 28.3 | 1837 | 25.7 | 30 | 0.38 | 32 | 0.44 | 37 | 0.46 | 87 | 1.3 |
| Morocco | 1916 | 8.5 | 1468 | 6.6 | 29 | 0.13 | 89 | 0.37 | 71 | 0.31 | 259 | 1.1 |
| Uzbekistan | 1903 | 11.1 | 1585 | 9.2 | 23 | 0.14 | 41 | 0.25 | 50 | 0.3 | 204 | 1.22 |
| Türkiye | 1799 | 2.9 | 1203 | 2 | 30 | 0.04 | 126 | 0.19 | 38 | 0.06 | 402 | 0.61 |
| Guinea | 1775 | 40.8 | 1695 | 38.9 | 15 | 0.31 | 9 | 0.19 | 13 | 0.32 | 43 | 1.08 |
| Canada | 1769 | 4.0 | 760 | 2.3 | 67 | 0.11 | 307 | 0.47 | 144 | 0.28 | 491 | 0.85 |
| Côte d'Ivoire | 1645 | 23.0 | 1461 | 20.4 | 42 | 0.58 | 19 | 0.24 | 36 | 0.42 | 87 | 1.36 |
| Mali | 1639 | 29.9 | 1431 | 26.1 | 21 | 0.38 | 25 | 0.46 | 25 | 0.46 | 137 | 2.5 |
| Egypt | 1616 | 3.5 | 820 | 1.8 | 39 | 0.09 | 137 | 0.29 | 54 | 0.11 | 566 | 1.2 |
| Nepal | 1584 | 10.6 | 1313 | 8.7 | 20 | 0.13 | 13 | 0.08 | 9 | 0.06 | 229 | 1.59 |
| Iran, Islamic Republic of | 1554 | 3.5 | 743 | 1.6 | 16 | 0.03 | 14 | 0.03 | 40 | 0.09 | 741 | 1.7 |
| Republic of Korea | 1543 | 2.5 | 1143 | 2 | 41 | 0.06 | 53 | 0.06 | 58 | 0.07 | 248 | 0.33 |
| Senegal | 1469 | 26.3 | 1327 | 23.7 | 23 | 0.37 | 21 | 0.36 | 23 | 0.4 | 75 | 1.42 |
| Korea, Democratic People Republic of | 1430 | 7.6 | 1237 | 6.8 | 12 | 0.06 | 30 | 0.15 | 10 | 0.04 | 141 | 0.57 |
| Sri Lanka | 1360 | 7.5 | 946 | 5.3 | 23 | 0.14 | 26 | 0.11 | 26 | 0.13 | 339 | 1.82 |
| Malaysia | 1348 | 7.2 | 1018 | 5.5 | 19 | 0.11 | 14 | 0.07 | 41 | 0.21 | 256 | 1.35 |
| Afghanistan | 1241 | 11.3 | 888 | 7.9 | 14 | 0.14 | 23 | 0.21 | 25 | 0.24 | 291 | 2.76 |
| Algeria | 1204 | 5.4 | 1013 | 4.6 | 24 | 0.1 | 36 | 0.15 | 27 | 0.11 | 104 | 0.43 |
| Bolivia | 1203 | 19.4 | 1138 | 18.3 | 11 | 0.2 | 10 | 0.18 | 0 | 0 | 44 | 0.68 |
| Burundi | 1177 | 38.2 | 1081 | 35 | 10 | 0.3 | 30 | 0.91 | 19 | 0.69 | 37 | 1.26 |
| Kazakhstan | 1165 | 9.5 | 918 | 7.7 | 28 | 0.21 | 48 | 0.34 | 24 | 0.17 | 147 | 1.11 |
| Ecuador | 1084 | 10.1 | 939 | 8.9 | 9 | 0.07 | 28 | 0.23 | 23 | 0.21 | 85 | 0.72 |
| Somalia | 1073 | 25.4 | 919 | 21.8 | 18 | 0.42 | 33 | 0.76 | 20 | 0.43 | 83 | 1.99 |
| Guatemala | 1055 | 13.5 | 973 | 12.5 | 7 | 0.08 | 8 | 0.1 | 3 | 0.04 | 64 | 0.74 |
| Cuba | 1043 | 8.9 | 695 | 6.4 | 28 | 0.21 | 37 | 0.24 | 39 | 0.34 | 244 | 1.74 |
| Chile | 1032 | 6.2 | 825 | 5.2 | 25 | 0.13 | 64 | 0.27 | 21 | 0.09 | 97 | 0.47 |
| Sudan | 997 | 6.9 | 738 | 5.2 | 20 | 0.13 | 72 | 0.52 | 22 | 0.14 | 145 | 0.94 |
| Burkina Faso | 980 | 16.3 | 775 | 13 | 22 | 0.35 | 26 | 0.3 | 73 | 1.1 | 84 | 1.5 |
| Chad | 975 | 21.2 | 841 | 18.4 | 13 | 0.33 | 20 | 0.43 | 0 | 0 | 101 | 2.08 |

| | | | | | | | | | | | |
|---|---|---|---|---|---|---|---|---|---|---|---|
| Cambodia | 912 | 11.3 | 670 | 8.1 | 5 | 0.06 | 16 | 0.2 | 10 | 0.13 | 211 | 2.77 |
| Hungary | 893 | 8.0 | 482 | 4.8 | 30 | 0.19 | 114 | 0.6 | 14 | 0.09 | 253 | 2.27 |
| Papua New Guinea | 865 | 25.5 | 686 | 19.9 | 17 | 0.51 | 5 | 0.15 | 16 | 0.52 | 141 | 4.42 |
| The Netherlands | 784 | 3.6 | 275 | 1.7 | 24 | 0.08 | 166 | 0.52 | 38 | 0.18 | 281 | 1.12 |
| Czechia | 780 | 6.0 | 369 | 3.2 | 30 | 0.17 | 103 | 0.53 | 58 | 0.41 | 220 | 1.7 |
| Australia | 768 | 2.8 | 323 | 1.4 | 30 | 0.1 | 103 | 0.26 | 78 | 0.26 | 234 | 0.73 |
| Portugal | 765 | 5.0 | 459 | 3.5 | 24 | 0.1 | 94 | 0.31 | 31 | 0.17 | 157 | 0.88 |
| Dominican Republic | 744 | 11.7 | 622 | 9.9 | 24 | 0.37 | 5 | 0.06 | 3 | 0.05 | 90 | 1.27 |
| Honduras | 732 | 17.1 | 669 | 15.6 | 8 | 0.19 | 6 | 0.13 | 12 | 0.28 | 37 | 0.86 |
| Serbia | 730 | 8.3 | 505 | 6.3 | 19 | 0.17 | 69 | 0.57 | 23 | 0.18 | 114 | 1.06 |
| Rwanda | 684 | 15.5 | 609 | 13.8 | 4 | 0.1 | 36 | 0.85 | 12 | 0.26 | 23 | 0.5 |
| South Sudan | 682 | 20.2 | 593 | 17.6 | 7 | 0.22 | 18 | 0.56 | 12 | 0.32 | 52 | 1.53 |
| Paraguay | 650 | 17.9 | 601 | 16.7 | 0 | 0 | 16 | 0.35 | 12 | 0.31 | 21 | 0.51 |
| Belarus | 618 | 6.4 | 371 | 4.4 | 10 | 0.06 | 84 | 0.52 | 30 | 0.25 | 123 | 1.12 |
| Bulgaria | 590 | 7.7 | 453 | 6.2 | 12 | 0.1 | 42 | 0.38 | 12 | 0.1 | 71 | 0.92 |
| Niger | 570 | 9.1 | 440 | 7.1 | 19 | 0.27 | 8 | 0.1 | 21 | 0.23 | 82 | 1.37 |
| Benin | 545 | 14.6 | 475 | 12.8 | 7 | 0.22 | 20 | 0.5 | 10 | 0.26 | 33 | 0.84 |
| Haiti | 539 | 10.2 | 451 | 8.6 | 12 | 0.23 | 0 | 0 | 4 | 0.06 | 72 | 1.3 |
| Sweden | 538 | 3.8 | 238 | 2 | 13 | 0.11 | 96 | 0.49 | 43 | 0.32 | 148 | 0.92 |
| Greece | 518 | 3.5 | 260 | 2.2 | 8 | 0.05 | 91 | 0.36 | 21 | 0.14 | 138 | 0.74 |
| Liberia | 513 | 30.4 | 478 | 28.3 | 8 | 0.44 | 3 | 0.16 | 6 | 0.32 | 18 | 1.14 |
| Belgium | 512 | 3.5 | 231 | 1.9 | 20 | 0.09 | 69 | 0.29 | 29 | 0.16 | 163 | 1.07 |
| Austria | 456 | 3.9 | 180 | 1.9 | 9 | 0.05 | 87 | 0.49 | 35 | 0.24 | 145 | 1.22 |
| Slovakia | 456 | 7.9 | 276 | 5.2 | 7 | 0.1 | 48 | 0.62 | 29 | 0.39 | 96 | 1.59 |
| Lesotho | 447 | 45.9 | 413 | 42.3 | 3 | 0.34 | 18 | 1.9 | 5 | 0.53 | 8 | 0.83 |
| Azerbaijan | 436 | 6.4 | 325 | 4.8 | 1 | 0.02 | 10 | 0.16 | 27 | 0.4 | 73 | 1.06 |
| El Salvador | 430 | 9.6 | 367 | 8.4 | 4 | 0.08 | 14 | 0.25 | 4 | 0.08 | 41 | 0.79 |
| Iraq | 423 | 3.2 | 216 | 1.6 | 10 | 0.08 | 14 | 0.11 | 10 | 0.08 | 173 | 1.34 |
| Nicaragua | 401 | 11.5 | 345 | 9.9 | 8 | 0.23 | 11 | 0.3 | 10 | 0.29 | 27 | 0.74 |
| Togo | 392 | 15.5 | 334 | 13.3 | 12 | 0.47 | 8 | 0.26 | 9 | 0.32 | 29 | 1.17 |
| Yemen | 360 | 4.0 | 153 | 1.6 | 28 | 0.32 | 10 | 0.13 | 5 | 0.04 | 164 | 1.95 |

| | | | | | | | | | | | | |
|---|---|---|---|---|---|---|---|---|---|---|---|---|
| Turkmenistan | 351 | 11.3 | 279 | 8.9 | 4 | 0.14 | 9 | 0.32 | 9 | 0.3 | 50 | 1.65 |
| Mauritania | 343 | 22.2 | 302 | 19.5 | 3 | 0.2 | 7 | 0.38 | 10 | 0.61 | 21 | 1.53 |
| Kyrgyzstan | 337 | 10.2 | 278 | 8.4 | 5 | 0.15 | 13 | 0.41 | 8 | 0.25 | 33 | 0.99 |
| Tunisia | 336 | 4.0 | 210 | 2.6 | 6 | 0.08 | 25 | 0.25 | 16 | 0.17 | 79 | 0.88 |
| Switzerland | 315 | 2.6 | 99 | 1.1 | 13 | 0.1 | 46 | 0.25 | 44 | 0.33 | 113 | 0.85 |
| Georgia | 302 | 7.4 | 210 | 5.8 | 9 | 0.13 | 34 | 0.57 | 6 | 0.12 | 43 | 0.81 |
| Denmark | 301 | 4.1 | 115 | 1.8 | 8 | 0.07 | 42 | 0.38 | 27 | 0.33 | 109 | 1.5 |
| Eswatini | 294 | 70.3 | 269 | 64.3 | 3 | 0.73 | 16 | 3.6 | 5 | 1.3 | 1 | 0.33 |
| Lao People's Democratic Republic | 294 | 9.7 | 200 | 6.5 | 7 | 0.23 | 9 | 0.28 | 5 | 0.16 | 73 | 2.55 |
| Sierra Leone | 294 | 11.0 | 292 | 10.9 | 0 | 0 | 0 | 0 | 1 | 0.02 | 1 | 0.03 |
| Saudi Arabia | 290 | 2.5 | 164 | 1.3 | 3 | 0.03 | 6 | 0.06 | 13 | 0.12 | 104 | 0.94 |
| Botswana | 288 | 25.6 | 253 | 22.8 | 3 | 0.19 | 15 | 1.2 | 8 | 0.59 | 9 | 0.86 |
| Moldova | 283 | 8.3 | 236 | 7.2 | 2 | 0.04 | 23 | 0.49 | 0 | 0 | 22 | 0.53 |
| Lithuania | 280 | 8.8 | 191 | 6.8 | 7 | 0.09 | 27 | 0.37 | 15 | 0.39 | 40 | 1.17 |
| Congo, Republic of | 277 | 16.1 | 248 | 14.2 | 2 | 0.12 | 10 | 0.77 | 10 | 0.53 | 7 | 0.5 |
| Croatia | 277 | 4.6 | 138 | 2.9 | 10 | 0.12 | 59 | 0.63 | 10 | 0.08 | 60 | 0.89 |
| Central African Republic | 274 | 20.7 | 240 | 18.3 | 2 | 0.14 | 9 | 0.71 | 9 | 0.63 | 14 | 0.95 |
| Syrian Arab Republic | 274 | 3.4 | 106 | 1.3 | 10 | 0.12 | 30 | 0.39 | 5 | 0.06 | 123 | 1.54 |
| Jamaica | 254 | 13.2 | 236 | 12.4 | 1 | 0.06 | 2 | 0.08 | 3 | 0.14 | 12 | 0.52 |
| Namibia | 249 | 25.4 | 203 | 20.5 | 6 | 0.57 | 9 | 0.85 | 3 | 0.37 | 28 | 3.13 |
| Uruguay | 241 | 6.8 | 160 | 5.2 | 5 | 0.09 | 21 | 0.4 | 13 | 0.25 | 42 | 0.88 |
| Norway | 237 | 3.3 | 104 | 1.7 | 3 | 0.02 | 45 | 0.48 | 10 | 0.15 | 75 | 0.97 |
| Israel | 233 | 3.2 | 142 | 2.2 | 6 | 0.06 | 34 | 0.35 | 3 | 0.03 | 48 | 0.54 |
| Panama | 229 | 8.2 | 192 | 7.1 | 5 | 0.14 | 5 | 0.14 | 6 | 0.16 | 21 | 0.66 |
| Singapore | 224 | 3.9 | 153 | 2.8 | 11 | 0.16 | 12 | 0.17 | 9 | 0.14 | 39 | 0.64 |
| Tajikistan | 217 | 5.8 | 159 | 4.1 | 0 | 0 | 4 | 0.13 | 9 | 0.27 | 45 | 1.26 |
| Libya | 216 | 6.6 | 169 | 5.1 | 7 | 0.21 | 7 | 0.22 | 3 | 0.08 | 30 | 0.98 |
| Bosnia Herzegovina | 211 | 6.3 | 151 | 4.9 | 5 | 0.13 | 18 | 0.39 | 2 | 0.05 | 35 | 0.78 |
| Costa Rica | 206 | 5.4 | 167 | 4.6 | 3 | 0.04 | 12 | 0.24 | 3 | 0.06 | 21 | 0.46 |
| The Republic of the Gambia | 205 | 27.5 | 204 | 27.4 | 0 | 0 | 0 | 0 | 0 | 0 | 1 | 0.14 |
| Latvia | 204 | 8.8 | 113 | 6 | 8 | 0.2 | 38 | 1 | 9 | 0.26 | 36 | 1.35 |

| | | | | | | | | | | | | |
|---|---|---|---|---|---|---|---|---|---|---|---|---|
| Finland | 190 | 2.6 | 65 | 1.3 | 12 | 0.12 | 40 | 0.35 | 10 | 0.14 | 63 | 0.68 |
| Ireland | 187 | 4.1 | 89 | 2.3 | 7 | 0.11 | 20 | 0.31 | 7 | 0.13 | 64 | 1.25 |
| Mongolia | 187 | 11.7 | 156 | 9.6 | 7 | 0.44 | 4 | 0.27 | 1 | 0.05 | 19 | 1.38 |
| Eritrea | 179 | 15.0 | 150 | 12.7 | 2 | 0.18 | 10 | 0.85 | 3 | 0.2 | 14 | 1.1 |
| Lebanon | 175 | 4.3 | 93 | 2.3 | 10 | 0.21 | 24 | 0.57 | 4 | 0.08 | 44 | 1.14 |
| New Zealand | 163 | 3.1 | 63 | 1.5 | 11 | 0.19 | 23 | 0.38 | 15 | 0.28 | 51 | 0.77 |
| Guinea-Bissau | 162 | 26.9 | 157 | 25.9 | 1 | 0.21 | 0 | 0 | 0 | 0 | 4 | 0.75 |
| Gabon | 154 | 19.7 | 139 | 17.8 | 2 | 0.16 | 0 | 0 | 4 | 0.33 | 9 | 1.41 |
| Albania | 151 | 5.7 | 97 | 4.1 | 8 | 0.23 | 15 | 0.46 | 1 | 0.02 | 30 | 0.92 |
| Puerto Rico | 146 | 4.3 | 100 | 3.4 | 9 | 0.17 | 9 | 0.11 | 7 | 0.12 | 21 | 0.48 |
| Trinidad and Tobago | 138 | 12.4 | 122 | 11.2 | 2 | 0.15 | 3 | 0.2 | 2 | 0.15 | 9 | 0.74 |
| Armenia | 131 | 4.9 | 99 | 4 | 5 | 0.18 | 8 | 0.21 | 2 | 0.07 | 17 | 0.48 |
| Jordan | 128 | 3.3 | 84 | 2.1 | 7 | 0.19 | 4 | 0.11 | 3 | 0.09 | 30 | 0.79 |
| Slovenia | 118 | 4.0 | 61 | 2.4 | 4 | 0.08 | 26 | 0.57 | 2 | 0.03 | 25 | 0.87 |
| Fiji | 110 | 23.6 | 105 | 22.5 | 1 | 0.21 | 0 | 0 | 0 | 0 | 4 | 0.86 |
| Comoros | 106 | 37.0 | 102 | 35.7 | 0 | 0 | 4 | 1.3 | 0 | 0 | 0 | 0 |
| United Arab Emirates | 91 | 5.3 | 69 | 3.5 | 0 | 0 | 0 | 0 | 2 | 0.19 | 20 | 1.64 |
| Estonia | 90 | 4.9 | 60 | 3.9 | 1 | 0.02 | 11 | 0.29 | 4 | 0.07 | 14 | 0.66 |
| Mauritius | 89 | 7.3 | 67 | 5.7 | 0 | 0 | 6 | 0.47 | 0 | 0 | 16 | 1.12 |
| North Macedonia | 86 | 4.6 | 61 | 3.5 | 2 | 0.11 | 5 | 0.19 | 0 | 0 | 18 | 0.78 |
| Equatorial Guinea | 83 | 24.1 | 76 | 21.9 | 1 | 0.14 | 0 | 0 | 2 | 0.43 | 4 | 1.6 |
| Guyana | 67 | 15.3 | 65 | 14.9 | 0 | 0 | 0 | 0 | 0 | 0 | 2 | 0.39 |
| Oman | 67 | 5.4 | 56 | 4.5 | 0 | 0 | 0 | 0 | 1 | 0.11 | 10 | 0.8 |
| Djibouti | 59 | 14.3 | 54 | 13 | 0 | 0 | 1 | 0.28 | 0 | 0 | 4 | 1 |
| Solomon Islands | 56 | 20.7 | 48 | 17.9 | 1 | 0.35 | 0 | 0 | 2 | 0.67 | 5 | 1.77 |
| Gaza Strip and West Bank | 51 | 3.3 | 37 | 2.3 | 2 | 0.16 | 2 | 0.16 | 0 | 0 | 10 | 0.69 |
| France, La Réunion | 50 | 6.1 | 39 | 5.1 | 0 | 0 | 4 | 0.32 | 3 | 0.24 | 4 | 0.39 |
| Montenegro | 49 | 8.4 | 32 | 6.3 | 1 | 0.13 | 1 | 0.15 | 2 | 0.26 | 13 | 1.54 |
| Kuwait | 47 | 3.1 | 34 | 2 | 0 | 0 | 0 | 0 | 0 | 0 | 13 | 1.13 |
| Suriname | 45 | 12.9 | 43 | 12.4 | 0 | 0 | 0 | 0 | 0 | 0 | 2 | 0.51 |
| Timor-Leste | 43 | 9.1 | 37 | 7.9 | 0 | 0 | 0 | 0 | 0 | 0 | 6 | 1.2 |

| | | | | | | | | | | | | |
|---|---|---|---|---|---|---|---|---|---|---|---|---|
| Cyprus | 42 | 3.0 | 31 | 2.4 | 0 | 0 | 7 | 0.33 | 2 | 0.08 | 2 | 0.16 |
| Bhutan | 37 | 10.9 | 26 | 7.2 | 0 | 0 | 0 | 0 | 0 | 0 | 11 | 3.69 |
| Bahamas | 33 | 12.0 | 33 | 12 | 0 | 0 | 0 | 0 | 0 | 0 | 0 | 0 |
| Cape Verde | 32 | 11.3 | 27 | 9.8 | 0 | 0 | 0 | 0 | 0 | 0 | 5 | 1.5 |
| Barbados | 26 | 8.2 | 24 | 7.9 | 0 | 0 | 0 | 0 | 0 | 0 | 2 | 0.32 |
| Maldives | 26 | 12.9 | 26 | 12.9 | 0 | 0 | 0 | 0 | 0 | 0 | 0 | 0 |
| Belize | 24 | 13.0 | 24 | 13 | 0 | 0 | 0 | 0 | 0 | 0 | 0 | 0 |
| Vanuatu | 23 | 17.6 | 18 | 13.6 | 0 | 0 | 0 | 0 | 1 | 0.72 | 4 | 3.3 |
| France, Guadeloupe | 21 | 4.4 | 16 | 4 | 0 | 0 | 3 | 0.26 | 1 | 0.12 | 1 | 0.06 |
| Malta | 18 | 2.7 | 6 | 1.4 | 0 | 0 | 5 | 0.59 | 0 | 0 | 7 | 0.66 |
| France, Martinique | 17 | 2.8 | 14 | 2.5 | 2 | 0.27 | 0 | 0 | 0 | 0 | 1 | 0.06 |
| New Caledonia | 17 | 8.3 | 16 | 8 | 0 | 0 | 0 | 0 | 0 | 0 | 1 | 0.27 |
| Brunei Darussalam | 16 | 6.6 | 16 | 6.6 | 0 | 0 | 0 | 0 | 0 | 0 | 0 | 0 |
| Qatar | 15 | 4.1 | 13 | 3.2 | 0 | 0 | 0 | 0 | 0 | 0 | 2 | 0.9 |
| Guam | 13 | 11.2 | 13 | 11.2 | 0 | 0 | 0 | 0 | 0 | 0 | 0 | 0 |
| Luxembourg | 13 | 2.0 | 10 | 1.7 | 1 | 0.17 | 2 | 0.13 | 0 | 0 | 0 | 0 |
| Saint Lucia | 13 | 10.4 | 13 | 10.4 | 0 | 0 | 0 | 0 | 0 | 0 | 0 | 0 |
| Bahrain | 11 | 2.2 | 8 | 1.6 | 0 | 0 | 0 | 0 | 0 | 0 | 3 | 0.6 |
| French Guyana | 10 | 6.7 | 10 | 6.7 | 0 | 0 | 0 | 0 | 0 | 0 | 0 | 0 |
| Sao Tome and Principe | 10 | 13.7 | 10 | 13.7 | 0 | 0 | 0 | 0 | 0 | 0 | 0 | 0 |
| Iceland | 9 | 2.8 | 8 | 2.7 | 0 | 0 | 0 | 0 | 0 | 0 | 1 | 0.12 |
| French Polynesia | 8 | 4.0 | 6 | 3.2 | 0 | 0 | 0 | 0 | 1 | 0.41 | 1 | 0.41 |
| Samoa | 6 | 7.4 | 6 | 7.4 | 0 | 0 | 0 | 0 | 0 | 0 | 0 | 0 |

**Supplementary Table S3: Definition of HDI regions**

| HDI region | Country | HDI region | Country | HDI region | Country |
|---|---|---|---|---|---|
| Very high HDI | Switzerland | Very high HDI | Czechia | Very high HDI | Georgia |
| Very high HDI | Norway | Very high HDI | Greece | Very high HDI | Mauritius |
| Very high HDI | Iceland | Very high HDI | Poland | Very high HDI | Serbia |
| Very high HDI | Australia | Very high HDI | Bahrain | Very high HDI | Thailand |
| Very high HDI | Denmark | Very high HDI | Lithuania | High HDI | Albania |
| Very high HDI | Sweden | Very high HDI | Saudi Arabia | High HDI | Bulgaria |
| Very high HDI | Ireland | Very high HDI | Portugal | High HDI | Barbados |
| Very high HDI | Germany | Very high HDI | Latvia | High HDI | Sri Lanka |
| Very high HDI | The Netherlands | Very high HDI | Croatia | High HDI | Bosnia Herzegovina |
| Very high HDI | Finland | Very high HDI | Chile | High HDI | Iran (Islamic Republic of) |
| Very high HDI | Singapore | Very high HDI | Qatar | High HDI | Ukraine |
| Very high HDI | Belgium | Very high HDI | Slovakia | High HDI | North Macedonia |
| Very high HDI | New Zealand | Very high HDI | Hungary | High HDI | China |
| Very high HDI | Canada | Very high HDI | Argentina | High HDI | Dominican Republic |
| Very high HDI | Luxembourg | Very high HDI | Türkiye | High HDI | Moldova |
| Very high HDI | United Kingdom | Very high HDI | Montenegro | High HDI | Cuba |
| Very high HDI | Japan | Very high HDI | Kuwait | High HDI | Peru |
| Very high HDI | Republic of Korea | Very high HDI | Brunei Darussalam | High HDI | Armenia |
| Very high HDI | United States of America | Very high HDI | Russian Federation | High HDI | Mexico |
| Very high HDI | Israel | Very high HDI | Romania | High HDI | Brazil |
| Very high HDI | Malta | Very high HDI | Oman | High HDI | Colombia |
| Very high HDI | Slovenia | Very high HDI | Bahamas | High HDI | Maldives |
| Very high HDI | Austria | Very high HDI | Kazakhstan | High HDI | Algeria |
| Very high HDI | United Arab Emirates | Very high HDI | Trinidad and Tobago | High HDI | Azerbaijan |
| Very high HDI | Spain | Very high HDI | Costa Rica | High HDI | Turkmenistan |
| Very high HDI | France (metropolitan) | Very high HDI | Uruguay | High HDI | Ecuador |
| Very high HDI | Cyprus | Very high HDI | Belarus | High HDI | Mongolia |
| Very high HDI | Italy | Very high HDI | Panama | High HDI | Tunisia |
| Very high HDI | Estonia | Very high HDI | Malaysia | High HDI | Egypt |

**Supplementary Table S3: Definition of HDI regions (Continued)**

| HDI region | Country | HDI region | Country | HDI region | Country |
|---|---|---|---|---|---|
| High HDI | Fiji | Medium HDI | India | Low HDI | Nigeria |
| High HDI | Suriname | Medium HDI | Ghana | Low HDI | Rwanda |
| High HDI | Uzbekistan | Medium HDI | Guatemala | Low HDI | Benin |
| High HDI | Jordan | Medium HDI | Honduras | Low HDI | Uganda |
| High HDI | Libya | Medium HDI | Sao Tome and Principe | Low HDI | Lesotho |
| High HDI | Paraguay | Medium HDI | Namibia | Low HDI | Malawi |
| High HDI | Gaza Strip and West Bank | Medium HDI | Lao People's Democratic Republic | Low HDI | Senegal |
| High HDI | Saint Lucia | Medium HDI | Vanuatu | Low HDI | Djibouti |
| High HDI | Guyana | Medium HDI | Timor-Leste | Low HDI | Sudan |
| High HDI | South Africa | Medium HDI | Nepal | Low HDI | Madagascar |
| High HDI | Jamaica | Medium HDI | Eswatini | Low HDI | The Republic of the Gambia |
| High HDI | Samoa | Medium HDI | Equatorial Guinea | Low HDI | Ethiopia |
| High HDI | Gabon | Medium HDI | Cambodia | Low HDI | Eritrea |
| High HDI | Lebanon | Medium HDI | Zimbabwe | Low HDI | Guinea-Bissau |
| High HDI | Indonesia | Medium HDI | Angola | Low HDI | Liberia |
| High HDI | Viet Nam | Medium HDI | Myanmar | Low HDI | Congo, Democratic Republic of |
| Medium HDI | Philippines | Medium HDI | Syrian Arab Republic | Low HDI | Afghanistan |
| Medium HDI | Botswana | Medium HDI | Cameroon | Low HDI | Sierra Leone |
| Medium HDI | Bolivia | Medium HDI | Kenya | Low HDI | Guinea |
| Medium HDI | Kyrgyzstan | Medium HDI | Congo, Republic of | Low HDI | Yemen |
| Medium HDI | Venezuela | Medium HDI | Zambia | Low HDI | Burkina Faso |
| Medium HDI | Iraq | Medium HDI | Solomon Islands | Low HDI | Mozambique |
| Medium HDI | Tajikistan | Medium HDI | Comoros | Low HDI | Mali |
| Medium HDI | Belize | Medium HDI | Papua New Guinea | Low HDI | Burundi |
| Medium HDI | Morocco | Medium HDI | Mauritania | Low HDI | Central African Republic |
| Medium HDI | El Salvador | Medium HDI | Côte d'Ivoire | Low HDI | Niger |
| Medium HDI | Nicaragua | Low HDI | Tanzania, United Republic of | Low HDI | Chad |
| Medium HDI | Bhutan | Low HDI | Pakistan | Low HDI | South Sudan |
| Medium HDI | Cape Verde | Low HDI | Togo | | |
| Medium HDI | Bangladesh | Low HDI | Haiti | | |

**Supplementary Table S4: International variations in average annual percentage change (AAPC) of HPV-related cancer incidence rates by site and sex**

| Countries | Registries | Period | Anus, females | | | Period | Anus, males | | |
|---|---|---|---|---|---|---|---|---|---|
| | | | APC | AAPC(%) | 95%CI | | APC | AAPC(%) | 95%CI |
| Australia | New South Wales & Australian Capital Territory, Queensland, South Tasmania, Victoria, Western, Northern Territory | 1983–1995 | 0.9 | | | 1983–2017 | 1.8* | | |
| | | 1995–2017 | 3.3* | | | 1983–2017 | | 1.8* | 1.5 to 2.3 |
| | | 1983–2017 | | 2.4* | 1.8 to 3.2 | 2003–2017 | | 1.5* | 0.5 to 2.7 |
| | | 2003–2017 | | 2.9* | 1.6 to 4.5 | | | | |
| Austria | National | 1998–2017 | 1.7* | | | 1998–2017 | 1.6* | | |
| | | 1998–2017 | | 1.7* | 0.7 to 2.9 | 1998–2017 | | 1.6* | 0.2 to 3.3 |
| | | 2003–2017 | | 2.3* | 0.9 to 3.8 | 2003–2017 | | 1.0 | -0.8 to 3.0 |
| Belarus | National | 1983–1997 | 19.4* | | | 1983–1997 | 21.4* | | |
| | | 1997–2002 | -30.4* | | | 1997–2002 | -37.8* | | |
| | | 2002–2017 | 10.2* | | | 2002–2017 | 6.1* | | |
| | | 1983–2017 | | 6.4* | 5.3 to 8.4 | 1983–2017 | | 3.7* | 2.5 to 5.5 |
| | | 2003–2017 | | 9.7* | 7.1 to 13.5 | 2003–2017 | | 6.7* | 3.7 to 10.7 |
| Canada | Excl Nova Scotia, Northwest Territories, Nunavut, Quebec and Yukon | 1978–1983 | 8.0* | | | 1978–1980 | 33.2* | | |
| | | 1983–1991 | -2.6* | | | 1980–2004 | 0.8 | | |
| | | 1991–2003 | 3.3* | | | 2004–2017 | -2.1 | | |
| | | 2003–2017 | 1.6 | | | 1978–2017 | | 1.3 | -0.05 to 2.4 |
| | | 1978–2017 | | 2.0* | 1.6 to 2.5 | 2003–2017 | | -2.3* | -3.9 to -0.7 |
| | | 2003–2017 | | 1.4* | 0.8 to 2.1 | | | | |
| China | Beijing City, Qidong City, Shanghai City, Jiashan County, Wuhan City, Zhongshan City, Nangang District (Harbin City), Yanting County | 1998–2017 | -2.0 | | | 1998–2017 | -2.4 | | |
| | | 1998–2017 | | -2.0 | -4.5 to 1.1 | 1998–2017 | | -2.4 | -4.5 to 0.1 |
| | | 2003–2017 | | -0.7 | -3.4 to 2.3 | 2003–2017 | | -1.3 | -3.3 to 0.9 |
| Costa Rica | National | 1984–2016 | 1.1 | | | 1992–2016 | 2.0 | | |
| | | 1984–2016 | | 1.1 | -0.4 to 3.6 | 1992–2016 | | 2.0 | -0.8 to 6.3 |
| | | 2003–2016 | | 1.5 | -3.1 to 5.3 | 2003–2016 | | -0.03 | -9.1 to 10.7 |
| Croatia | National | 1988–2017 | 3.1* | | | 1988–2007 | 1.3 | | |

| | | | | | | | | | |
|---|---|---|---|---|---|---|---|---|---|
| | | 1988–2017 | | 3.1* | 1.8 to 4.6 | 2007–2017 | 10.0* | | |
| | | 2003–2017 | | 0.9 | -2.5 to 6.6 | 1988–2017 | | 4.2* | 2.0 to 6.7 |
| | | | | | | 2003–2017 | | 7.8* | 4.2 to 12.7 |
| Czech Republic | National | 1983–1992 | 3.2 | | | 1983–1993 | 1.7 | | |
| | | 1992–1995 | -20.4* | | | 1993–1998 | -17.5* | | |
| | | 1995–2017 | 4.3* | | | 1998–2017 | 3.0* | | |
| | | 1983–2017 | | 1.5* | 1.0 to 2.6 | 1983–2017 | | -0.7 | -1.3 to 0.1 |
| | | 2003–2017 | | 4.1* | 2.6 to 5.8 | 2003–2017 | | 2.1* | 0.03 to 4.5 |
| Denmark | National | 1978–2017 | 3.3* | | | 1978–2017 | 1.8* | | |
| | | 1978–2017 | | 3.3* | 2.9 to 4.0 | 1978–2017 | | 1.8* | 1.3 to 2.4 |
| | | 2003–2017 | | 3.2* | 0.9 to 5.7 | 2003–2017 | | 2.0* | 0.4 to 3.8 |
| France | Martinique, Bas-Rhin, Calvados, Doubs, Haut-Rhin, Isère, Somme, Hérault, Loire-Atlantique, Manche, Vendée | 1979–2003 | 0.5 | | | 1979–2002 | -1.1 | | |
| | | 2003–2017 | 4.7* | | | 2002–2017 | 2.9* | | |
| | | 1979–2017 | | 2.0* | 1.4 to 2.8 | 1979–2017 | | 0.5 | -0.8 to 1.7 |
| | | 2003–2017 | | 4.6* | 2.2 to 7.5 | 2003–2017 | | 2.8* | 0.2 to 5.8 |
| Germany | Hamburg, Bremen, Schleswig-Holstein, Saarland | 1998–2017 | 3.4* | | | 1998–2017 | 3.3* | | |
| | | 1998–2017 | | 3.4* | 1.7 to 5.8 | 1998–2017 | | 3.3* | 2.1 to 4.8 |
| | | 2003–2017 | | 2.9* | 0.8 to 5.4 | 2003–2017 | | 3.1* | 1.6 to 4.8 |
| India | Mumbai, Chennai, Barshi, Dindigul Ambilikkai | 1978–2017 | 0.04 | | | 1978–2017 | -0.7 | | |
| | | 1978–2017 | | 0.04 | -0.6 to 1.0 | 1978–2017 | | -0.7 | -1.3 to 0.1 |
| | | 2003–2017 | | 1.0 | -2.3 to 4.7 | 2003–2017 | | 0.02 | -2.2 to 2.5 |
| Ireland | National | 1994–2017 | 3.6* | | | 1994–2017 | 1.8* | | |
| | | 1994–2017 | | 3.6* | 2.0 to 5.8 | 1994–2017 | | 1.8* | 0.3 to 3.6 |
| | | 2003–2017 | | 3.3* | 0.2 to 6.9 | 2003–2017 | | 2.4 | -1.1 to 6.5 |
| Israel | National | 1978–2017 | -0.5 | | | 1978–2017 | -1.6* | | |
| | | 1978–2017 | | -0.5 | -1.1 to 0.3 | 1978–2017 | | -1.6* | -2.5 to -0.7 |
| | | 2003–2017 | | 0.2 | -1.6 to 2.9 | 2003–2017 | | 2.5 | -0.6 to 6.4 |
| Japan | Miyagi Prefecture, Osaka | 1978–2017 | 1.2* | | | 1978–2017 | 1.7* | | |
| | | 1978–2017 | | 1.2* | 0.5 to 2.1 | 1978–2017 | | 1.7* | 1.0 to 2.8 |

| | | | | | | | | | |
|---|---|---|---|---|---|---|---|---|---|
| | | 2003–2017 | | 2.2 | -1.0 to 5.6 | 2003–2017 | | -1.5 | -7.0 to 3.6 |
| Lithuania | National | 1988–2017 | 2.4* | | | 1988–2017 | -0.3 | | |
| | | 1988–2017 | | 2.4* | 1.0 to 4.2 | 1988–2017 | | -0.3 | -2.6 to 2.2 |
| | | 2003–2017 | | 4.6* | 0.4 to 9.6 | 2003–2017 | | 1.9 | -4.1 to 8.9 |
| New Zealand | National | 1983–2017 | 1.8* | | | 1983–1995 | 4.7* | | |
| | | 1983–2017 | | 1.8* | 1.0 to 2.8 | 1995–2005 | -6.4* | | |
| | | 2003–2017 | | -0.6 | -3.2 to 2.3 | 2005–2014 | 7.7* | | |
| | | | | | | 2014–2017 | -16.7* | | |
| | | | | | | 1983–2017 | | 0.03 | -1.1 to 1.4 |
| | | | | | | 2003–2017 | | 0.6 | -6.0 to 10.5 |
| Norway | National | 1978–2017 | 1.9* | | | 1978–2017 | 1.8* | | |
| | | 1978–2017 | | 1.9* | 1.3 to 2.7 | 1978–2017 | | 1.8* | 0.9 to 2.9 |
| | | 2003–2017 | | 2.4 | -0.5 to 5.9 | 2003–2017 | | 1.1 | -3.6 to 6.4 |
| Philippines | National | 1983–2017 | -0.8 | | | 1995–2014 | -0.8 | | |
| | | 1983–2017 | | -0.8 | -2.4 to 1.4 | 2014–2017 | -45.6 | | |
| | | 2003–2017 | | -3.2 | -8.9 to 2.8 | 1995–2017 | | -8.6* | -15.2 to -1.9 |
| | | | | | | 2003–2017 | | -12.4* | -22.8 to -1.0 |
| Republic of Korea | National | 1993–1997 | -41.1* | | | 1993–1997 | -37.3* | | |
| | | 1997–2017 | 1.8* | | | 1997–2017 | -1.8* | | |
| | | 1993–2017 | | -7.1* | -8.0 to -5.9 | 1993–2017 | | -8.9* | -9.9 to -7.3 |
| | | 2003–2017 | | 2.2* | 0.2 to 4.6 | 2003–2017 | | -1.8* | -3.0 to -0.6 |
| Spain | Tarragona, Granada, Murcia, Navarra, Basque Country, Girona, Canary Islands, La Rioja | 1986–2017 | 1.9* | | | 1986–2017 | 2.0* | | |
| | | 1986–2017 | | 1.9* | 0.8 to 3.3 | 1986–2017 | | 2.0* | 0.9 to 3.5 |
| | | 2003–2017 | | 4.8* | 1.7 to 8.4 | 2003–2017 | | 3.3 | -0.4 to 7.5 |
| Thailand | Chiang Mai, Khon Kaen, Songkhla, Lampang | 1993–2017 | 0.2 | | | 1993–2017 | 4.4* | | |
| | | 1993–2017 | | 0.2 | -2.1 to 3.6 | 1993–2017 | | 4.4* | 2.3 to 7.9 |
| | | 2003–2017 | | 2.0 | -2.8 to 8.0 | 2003–2017 | | 7.3* | 0.9 to 16.5 |
| The Netherlands | National | 1989–2017 | 3.9* | | | 1989–1994 | -3.2 | | |
| | | 1989–2017 | | 3.9* | 3.3 to 4.7 | 1994–2017 | 4.6* | | |

| | | | | | | | | | |
|---|---|---|---|---|---|---|---|---|---|
| | | 2003–2017 | | 4.2* | 2.6 to 6.2 | 1989–2017 | | 3.1* | 2.4 to 4.4 |
| | | | | | | 2003–2017 | | 4.6* | 2.7 to 6.9 |
| Türkiye | Izmir, Antalya | 1998–2017 | -0.2 | | | 1998–2017 | -0.05 | | |
| | | 1998–2017 | | -0.2 | -4.5 to 5.4 | 1998–2017 | | -0.05 | -2.4 to 2.8 |
| | | 2003–2017 | | -1.5 | -8.3 to 6.2 | 2003–2017 | | -0.05 | -4.4 to 5.0 |
| UK | England, Scotland, Northern Ireland, Wales | 1978–1980 | 33.6* | | | 1978–1989 | 3.7* | | |
| | | 1980–2005 | 3.5 | | | 1989–2017 | 1.7* | | |
| | | 2005–2017 | 5.0* | | | 1978–2017 | | 2.3* | 2.0 to 2.7 |
| | | 1978–2017 | | 5.3* | 4.6 to 6.2 | 2003–2017 | | 1.7* | 1.1 to 2.3 |
| | | 2003–2017 | | 5.0* | 4.2 to 6.0 | | | | |
| USA | California, Los Angeles County; USA, SEER (9 registries); USA, NPCR (45 States and Washington DC) | 1978–1997 | 1.0 | | | 1978–2000 | 1.5 | | |
| | | 1997–2009 | 3.8* | | | 2000–2009 | 2.8* | | |
| | | 2009–2017 | 2.0* | | | 2009–2017 | 0.04 | | |
| | | 1978–2017 | | 2.0* | 1.8 to 2.3 | 1978–2017 | | 1.5* | 1.1 to 1.8 |
| | | 2003–2017 | | 2.7* | 2.2 to 3.3 | 2003–2017 | | 1.1* | 0.2 to 2.2 |

APC, annual percentage change; AAPC, Average annual percentage change

* Statistically significant (P<0.05)

**Supplementary Table S4: International variations in average annual percentage change (AAPC) of HPV-related cancer incidence rates by site and sex (Continued)**

| Countries | Registries | Period | Vulva, females | | | Period | Vagina, females | | |
|---|---|---|---|---|---|---|---|---|---|
| | | | APC | AAPC(%) | 95%CI | | APC | AAPC(%) | 95%CI |
| Australia | New South Wales & Australian Capital Territory, Queensland, South Tasmania, Victoria, Western, Northern Territory | 1983–2017 | 1.2 | | | 1983–2014 | -0.4 | | |
| | | 1983–2017 | | 1.2* | 0.9 to 1.6 | 2014–2017 | 10.3* | | |
| | | 2003–2017 | | 1.9* | 0.4 to 3.7 | 1983–2017 | | 0.5 | -0.3 to 1.1 |
| | | | | | | 2003–2017 | | 1.5 | -0.9 to 3.5 |
| Austria | National | 1998–2017 | 0.2 | | | 1998–2017 | -1.6 | | |
| | | 1998–2017 | | 0.2 | -0.4 to 0.8 | 1998–2017 | | -1.6 | -4.2 to 0.9 |
| | | 2003–2017 | | 0.2 | -0.7 to 1.2 | 2003–2017 | | -8.1* | -11.5 to -5.8 |
| Belarus | National | 1989–1992 | 68.8* | | | 1989–2001 | 16.4* | | |
| | | 1992–2000 | 14.7 | | | 2001–2017 | 0.7 | | |
| | | 2000–2017 | -0.3 | | | 1989–2017 | | 7.2* | 4.7 to 12.2 |
| | | 1989–2017 | | 9.8* | 6.1 to 15.4 | 2003–2017 | | 0.4 | -2.0 to 2.8 |
| | | 2003–2017 | | -0.2 | -1.6 to 1.3 | | | | |
| Canada | Excl Nova Scotia, Northwest Territories, Nunavut, Quebec and Yukon | 1978–2003 | -0.1 | | | 1978–2008 | -1.4* | | |
| | | 2003–2017 | 3.3* | | | 2008–2017 | 4.9* | | |
| | | 1978–2017 | | 1.1* | 0.8 to 1.5 | 1978–2017 | | 0.1 | -0.5 to 0.6 |
| | | 2003–2017 | | 3.4* | 2.5 to 4.6 | 2003–2017 | | 1.6 | -0.7 to 5.6 |
| China | Beijing City, Qidong City, Shanghai City, Jiashan County, Wuhan City, Zhongshan City, Nangang District (Harbin City), Yanting County | 1999–2017 | 2.2* | | | 1998–2017 | 1.6 | | |
| | | 1999–2017 | | 2.2* | 0.2 to 4.7 | 1998–2017 | | 1.6 | -1.1 to 5.1 |
| | | 2003–2017 | | 1.9 | -1 to 5.2 | 2003–2017 | | 1.4 | -2.7 to 6.1 |
| Costa Rica | National | 1982–2016 | -0.1 | | | 1982–2005 | -6.9* | | |
| | | 1982–2016 | | -0.1 | -1.0 to 1.1 | 2005–2009 | 36.8* | | |
| | | 2003–2016 | | -2.7 | -5.8 to 0.6 | 2009–2016 | -5.4 | | |
| | | | | | | 1982–2016 | | -2.2* | -3.5 to -1.1 |
| | | | | | | 2003–2016 | | 9.4* | 3.4 to 20.4 |
| Croatia | National | 1988–2017 | 1.1* | | | 1988–2017 | -1.2 | | |
| | | 1988–2017 | | 1.1* | 0.5 to 1.8 | 1988–2017 | | -1.2 | -2.9 to 0.6 |

| | | | | | | | | | |
|---|---|---|---|---|---|---|---|---|---|
| | | 2003–2017 | | 1.5 | -0.04 to 3.2 | 2003–2017 | | -0.1 | -5.5 to 5.6 |
| Czech Republic | National | 1983–2017 | 0.8* | | | 1983–2017 | -1.1* | | |
| | | 1983–2017 | | 0.8* | 0.4 to 1.2 | 1983–2017 | | -1.1* | -1.7 to -0.5 |
| | | 2003–2017 | | 0.3 | -1.5 to 2.2 | 2003–2017 | | 0.4 | -1.9 to 2.6 |
| Denmark | National | 1978–2000 | -0.6 | | | 1978–2017 | -2.0* | | |
| | | 2000–2017 | 2.8* | | | | | | |
| | | 1978–2017 | | 0.9* | 0.4 to 1.4 | 1978–2017 | | -2.0* | -2.7 to -1.2 |
| | | 2003–2017 | | 3.2* | 2.1 to 4.3 | 2003–2017 | | -1.3 | -4.2 to 1.7 |
| France | Martinique, Bas-Rhin, Calvados, Doubs, Haut-Rhin, Isère, Somme, Hérault, Loire-Atlantique, Manche, Vendée | 1979–1991 | -4.1* | | | 1979–2015 | -2.7* | | |
| | | 1991–2017 | 0.2 | | | 2015–2017 | 37.9 | | |
| | | 1979–2017 | | -1.2* | -1.7 to -0.5 | 1979–2017 | | -0.9 | -2.8 to 0.2 |
| | | 2003–2017 | | 0.4 | -0.8 to 1.7 | 2003–2017 | | 2.7 | -0.9 to 5.1 |
| Germany | Hamburg, Bremen, Schleswig-Holstein, Saarland | 1998–2005 | 23.7* | | | 1998–2017 | 0.5 | | |
| | | 2005–2010 | 7.2* | | | 1998–2017 | | 0.5 | -2.0 to 3.6 |
| | | 2010–2017 | -1.6 | | | 2003–2017 | | -0.7 | -4.8 to 3.7 |
| | | 1998–2017 | | 9.5* | 8.1 to 12.4 | | | | |
| | | 2003–2017 | | 5.6* | 4.0 to 7.5 | | | | |
| India | Mumbai, Chennai, Barshi, Dindigul Ambilikkai | 1978–2017 | -1.8* | | | 1978–2013 | -1.1 | | |
| | | 1978–2017 | | -1.8* | -2.4 to -1.0 | 2013–2017 | -9.6* | | |
| | | 2003–2017 | | 0.9 | -3.7 to 6.1 | 1978–2017 | | -2.0* | -2.8 to -0.7 |
| | | | | | | 2003–2017 | | -3.6* | -5.8 to -1.0 |
| Ireland | National | 1994–2017 | 1.3* | | | 1994–2017 | -1.1 | | |
| | | 1994–2017 | | 1.3* | 0.1 to 2.7 | 1994–2017 | | -1.1 | -3.8 to 1.8 |
| | | 2003–2017 | | 0.7 | -1.6 to 3.2 | 2003–2017 | | -3.1 | -8.7 to 3.0 |
| Israel | National | 1978–2017 | -0.6 | | | 1978–2017 | 0.5 | | |
| | | 1978–2017 | | -0.6 | -1.0 to 0.04 | 1978–2017 | | 0.5 | -0.5 to 1.9 |
| | | 2003–2017 | | 0.5 | -2.0 to 3.3 | 2003–2017 | | -2.0 | -6.2 to 2.4 |
| Japan | Miyagi Prefecture, Osaka | 1978–1991 | -4.4* | | | 1978–2017 | -0.9* | | |
| | | 1991–2017 | 2.3* | | | | | | |

| | | | | | | | | | |
|---|---|---|---|---|---|---|---|---|---|
| | | 1978–2017 | | 0.01 | -0.9 to 1.0 | 1978–2017 | | -0.9* | -1.7 to -0.1 |
| | | 2003–2017 | | 2.0 | -2.4 to 6.5 | 2003–2017 | | 2.1 | -3.1 to 7.3 |
| Lithuania | National | 1988–2017 | 1.2* | | | 1988–2017 | 0.4 | | |
| | | 1988–2017 | | 1.2* | 0.2 to 2.3 | 1988–2017 | | 0.4 | -1.2 to 2.1 |
| | | 2003–2017 | | -1.1 | -2.9 to 0.7 | 2003–2017 | | 1.8 | -3.4 to 7.4 |
| New Zealand | National | 1983–2017 | -0.1 | | | 1983–1988 | 24.4* | | |
| | | 1983–2017 | | -0.1 | -0.7 to 0.8 | 1988–1996 | -12.0* | | |
| | | 2003–2017 | | -0.1 | -3.2 to 3.3 | 1996–2017 | 0.4 | | |
| | | | | | | 1983–2017 | | 0.4 | -1.0 to 3.3 |
| | | | | | | 2003–2017 | | 1.4 | -2.9 to 6.2 |
| Norway | National | 1978–2017 | 0.9* | | | 1978–2017 | 0.2 | | |
| | | 1978–2017 | | 0.9* | 0.6 to 1.4 | 1978–2017 | | 0.2 | -0.7 to 1.2 |
| | | 2003–2017 | | 0.6 | -1.1 to 2.4 | 2003–2017 | | 0.03 | -3.5 to 4.7 |
| Philippines | National | 1983–2017 | -1.8* | | | 1983–2017 | -3.3* | | |
| | | 1983–2017 | | -1.8* | -3.1 to -0.2 | 1983–2017 | | -3.3* | -5.2 to -1.4 |
| | | 2003–2017 | | -1.9 | -7.2 to 4.1 | 2003–2017 | | -11.9* | -17.8 to -8.5 |
| Republic of Korea | National | 1993–2017 | 2.1* | | | 1993–2017 | -1.1 | | |
| | | 1993–2017 | | 2.1* | 1.3 to 3.3 | 1993–2017 | | -1.1 | -2.2 to 0.4 |
| | | 2003–2017 | | 2.6* | 1.4 to 4.0 | 2003–2017 | | -0.8 | -3.1 to 1.6 |
| Spain | Tarragona, Granada, Murcia, Navarra, Basque Country, Girona, Canary Islands, La Rioja | 1986–2017 | -0.03 | | | 1987–2017 | -0.5 | | |
| | | 1986–2017 | | -0.03 | -0.6 to 0.6 | 1987–2017 | | -0.5 | -1.5 to 0.9 |
| | | 2003–2017 | | 0.9 | -1.2 to 3.1 | 2003–2017 | | 1.7 | -1.7 to 5.6 |
| Thailand | Chiang Mai, Khon Kaen, Songkhla, Lampang | 1993–2017 | -3.2* | | | 1993–2017 | -4.2* | | |
| | | 1993–2017 | | -3.2* | -5.0 to -1.1 | 1993–2017 | | -4.2* | -7.3 to -0.7 |
| | | 2003–2017 | | -1.9 | -6.3 to 2.7 | 2003–2017 | | -3.3 | -11.1 to 5.2 |
| The Netherlands | National | 1989–2005 | 0.5 | | | 1989–1997 | -3.8 | | |
| | | 2005–2009 | 8.7* | | | 1997–2017 | 0.3 | | |
| | | 2009–2017 | 0.3 | | | 1989–2017 | | -0.9 | -1.8 to 0.2 |

| | | | | | | | | | |
|---|---|---|---|---|---|---|---|---|---|
| | | 1989–2017 | | 1.6* | 1.1 to 1.9 | 2003–2017 | | 0.2 | -2.0 to 2.4 |
| | | 2003–2017 | | 2.8* | 0.2 to 6.1 | | | | |
| Türkiye | Izmir, Antalya | 1998–2000 | 43.8 | | | 1998–2017 | 0.4 | | |
| | | 2000–2017 | -0.1 | | | 1998–2017 | | 0.4 | -2.3 to 3.6 |
| | | 1998–2017 | | 3.8 | -1.2 to 8.7 | 2003–2017 | | 0.8 | -4.5 to 7.3 |
| | | 2003–2017 | | 0.4 | -2.4 to 3.8 | | | | |
| UK | England, Scotland, Northern Ireland, Wales | 1978–1992 | -0.5 | | | 1978–2017 | 0.01 | | |
| | | 1992–2013 | 1.6 | | | 1978–2017 | | 0.01 | -0.2 to 0.3 |
| | | 2013–2017 | -1.0 | | | 2003–2017 | | -0.6 | -1.4 to 0.2 |
| | | 1978–2017 | | 0.5* | 0.3 to 0.8 | | | | |
| | | 2003–2017 | | 0.9* | 0.1 to 1.8 | | | | |
| USA | California, Los Angeles County; USA, SEER (9 registries); USA, NPCR (45 States and Washington DC) | 1978–2017 | 0.9* | | | 1978–2017 | -0.6* | | |
| | | 1978–2017 | | 0.9* | 0.8 to 1.1 | 1978–2017 | | -0.6* | -0.8 to -0.4 |
| | | 2003–2017 | | 0.9* | 0.3 to 1.7 | 2003–2017 | | -0.4 | -1.0 to 0.2 |

APC, annual percentage change; AAPC, Average annual percentage change

* Statistically significant (P<0.05)

**Supplementary Table S4: International variations in average annual percentage change (AAPC) of HPV-related cancer incidence rates by site and sex (Continued)**

| Countries | Registries | Period | Cervix uteri, females | | | Period | Penis, males | | |
|---|---|---|---|---|---|---|---|---|---|
| | | | APC | AAPC(%) | 95%CI | | APC | AAPC(%) | 95%CI |
| Australia | New South Wales & Australian Capital Territory, Queensland, South Tasmania, Victoria, Western, Northern Territory | 1983–1994 | -1.5 | | | 1983–2017 | 0.5* | | |
| | | 1994–2001 | -7.3* | | | 1983–2017 | | 0.5* | 0.01 to 1.1 |
| | | 2001–2017 | 0.1 | | | 2003–2017 | | 1.4 | -0.3 to 3.4 |
| | | 1983–2017 | | -2.0* | -2.2 to -1.8 | | | | |
| | | 2003–2017 | | 0.2 | -0.4 to 0.8 | | | | |
| Austria | National | 1998–2010 | -3.6* | | | 1998–2017 | 0.6 | | |
| | | 2010–2017 | -0.2 | | | 1998–2017 | | 0.6 | -1.4 to 2.8 |
| | | 1998–2017 | | -2.4* | -3.1 to -1.7 | 2003–2017 | | 0.9 | -2.2 to 4.3 |
| | | 2003–2017 | | -2.1* | -2.9 to -1.2 | | | | |
| Belarus | National | 1983–1998 | -1.0* | | | 1983–1987 | -10.5 | | |
| | | 1998–2008 | 2.1* | | | 1987–2017 | 1.7* | | |
| | | 2008–2017 | -0.5 | | | 1983–2017 | | 0.2 | -0.7 to 1.5 |
| | | 1983–2017 | | 0.03 | -0.2 to 0.2 | 2003–2017 | | 2.0 | -0.5 to 4.8 |
| | | 2003–2017 | | 0.9* | 0.5 to 1.4 | | | | |
| Canada | Excl Nova Scotia, Northwest Territories, Nunavut, Quebec and Yukon | 1978–2004 | -2.0* | | | 1978–2011 | -0.8 | | |
| | | 2004–2017 | -0.3 | | | 2011–2014 | 15.2 | | |
| | | 1978–2017 | | -1.5* | -1.6 to -1.3 | 2014–2017 | -7.8 | | |
| | | 2003–2017 | | -0.4 | -1.2 to 0.5 | 1978–2017 | | -0.2 | -0.8 to 0.7 |
| | | | | | | 2003–2017 | | 1.0 | -0.3 to 2.1 |
| China | Beijing City, Qidong City, Shanghai City, Jiashan County, Wuhan City, Zhongshan City, Nangang District (Harbin City), Yanting County | 1998–2007 | 10.4* | | | 1998–2017 | -0.2 | | |
| | | 2007–2017 | 4.0* | | | 1998–2017 | | -0.2 | -1.8 to 1.6 |
| | | 1998–2017 | | 7.0* | 6.1 to 9.0 | 2003–2017 | | 0.6 | -0.8 to 2.2 |
| | | 2003–2017 | | 6.0* | 5.2 to 7.0 | | | | |
| Costa Rica | National | 1982–2000 | -1.8* | | | 1982–2016 | -0.7* | | |
| | | 2000–2006 | -6.5* | | | 1982–2016 | | -0.7 | -1.6 to 0.6 |
| | | 2006–2016 | -1.2 | | | 2003–2016 | | -1.9 | -5.0 to 1.5 |
| | | 1982–2016 | | -2.4* | -2.7 to -2.1 | | | | |

| | | | | | | | | | |
|---|---|---|---|---|---|---|---|---|---|
| | | 2003–2016 | | -2.9* | -4.2 to -1.3 | | | | |
| Croatia | National | 1988–2017 | -0.8* | | | 1988–2017 | 3.1 | | |
| | | 1988–2017 | | -0.8* | -1.2 to -0.3 | 1988–2017 | | 3.1 | 1.8 to 5.0 |
| | | 2003–2017 | | -0.1 | -2.3 to 1.6 | 2003–2017 | | 2.9 | -0.3 to 6.7 |
| Czech Republic | National | 1983–1994 | -0.1 | | | 1983–2017 | 1.9* | | |
| | | 1994–2010 | -1.5* | | | 1983–2017 | | 1.9 | 1.3 to 2.5 |
| | | 2010–2017 | -4.5* | | | 2003–2017 | | 1.3 | -0.6 to 3.4 |
| | | 1983–2017 | | -1.7* | -2.0 to -1.4 | | | | |
| | | 2003–2017 | | -2.9* | -3.8 to -2.2 | | | | |
| Denmark | National | 1978–2002 | -2.5* | | | 1978–2017 | 0.5* | | |
| | | 2002–2017 | -0.4 | | | 1978–2017 | | 0.5* | 0.04 to 1 |
| | | 1978–2017 | | -1.7* | -2 to -1.5 | 2003–2017 | | 1.3 | -0.4 to 3.1 |
| | | 2003–2017 | | -0.5 | -1.4 to 0.5 | | | | |
| France | Martinique, Bas-Rhin, Calvados, Doubs, Haut-Rhin, Isère, Somme, Hérault, Loire-Atlantique, Manche, Vendée | 1979–2010 | -3.0* | | | 1979–2017 | -0.5 | | |
| | | 2010–2017 | 1.4 | | | 1979–2017 | | -0.5 | -1.1 to 0.1 |
| | | 1979–2017 | | -2.2* | -2.5 to -1.9 | 2003–2017 | | -0.4 | -2.2 to 1.5 |
| | | 2003–2017 | | -0.8 | -2.1 to 0.5 | | | | |
| Germany | Hamburg, Bremen, Schleswig-Holstein, Saarland | 1998–2017 | -0.8* | | | 1998–2017 | 1.6* | | |
| | | 1998–2017 | | -0.8* | -1.3 to -0.2 | 1998–2017 | | 1.6* | 0.3 to 3.3 |
| | | 2003–2017 | | -0.8 | -2.0 to 0.4 | 2003–2017 | | 1.0 | -0.5 to 2.7 |
| India | Mumbai, Chennai, Barshi, Dindigul Ambilikkai | 1978–1985 | 7.3* | | | 1978–2017 | -2.5* | | |
| | | 1985–2007 | -2.6* | | | 1978–2017 | | -2.5* | -2.9 to -2.0 |
| | | 2007–2017 | -4.6* | | | 2003–2017 | | -3.4* | -4.6 to -2.3 |
| | | 1978–2017 | | -1.4* | -1.8 to -0.9 | | | | |
| | | 2003–2017 | | -3.5* | -4.6 to -2.6 | | | | |
| Ireland | National | 1994–1999 | -4.5 | | | 1994–2017 | 0.7 | | |
| | | 1999–2010 | 4.0 | | | 1994–2017 | | 0.7 | -0.5 to 2.2 |
| | | 2010–2017 | -3.9 | | | 2003–2017 | | 1.9* | 0.2 to 3.9 |
| | | 1994–2017 | | -0.4 | -1.6 to 1.0 | | | | |
| | | 2003–2017 | | 0.5 | -1.6 to 3.0 | | | | |
| Israel | National | 1978–1986 | -0.6 | | | 1988–2017 | 4.1 | | |

| | | | | | | | | | |
|---|---|---|---|---|---|---|---|---|---|
| | | 1986–1989 | 13.1 | | | 1988–2017 | | 4.1* | 2.2 to 7.7 |
| | | 1989–2017 | -0.1 | | | 2003–2017 | | 1.4 | -2.7 to 6.6 |
| | | 1978–2017 | | 0.7* | 0.3 to 1.3 | | | | |
| | | 2003–2017 | | -0.3 | -1.0 to 0.4 | | | | |
| Japan | Miyagi Prefecture, Osaka | 1978–1998 | -5.0* | | | 1978–1996 | -5.1* | | |
| | | 1998–2015 | 4.0* | | | 1996–2017 | 2.2* | | |
| | | 1983–2017 | | 0.6* | 0.2 to 1.0 | 1978–2017 | | -1.2* | -2.0 to -0.5 |
| | | 1978–2017 | | -2.3* | -3.2 to -1.7 | 2003–2017 | | 2.6 | -1.7 to 7.3 |
| | | 2003–2017 | | -0.4 | -3.9 to 1.2 | | | | |
| Lithuania | National | 1988–2004 | 3.7* | | | 1988–1998 | 11.6* | | |
| | | 2004–2017 | -2.6* | | | 1998–2017 | 1.2 | | |
| | | 1988–2017 | | 0.8* | 0.3 to 1.3 | 1988–2017 | | 4.7* | 2.8 to 8.3 |
| | | 2003–2017 | | -2.5* | -4.1 to -1.1 | 2003–2017 | | 1.9 | -2.2 to 6.3 |
| New Zealand | National | 1983–1988 | 2.0 | | | 1983–2017 | -0.6 | | |
| | | 1988–2005 | -4.7* | | | 1983–2017 | | -0.6 | -1.4 to 0.4 |
| | | 2005–2017 | -1.1 | | | 2003–2017 | | 1.9 | -1.1 to 5.5 |
| | | 1983–2017 | | -2.5* | -2.9 to -1.9 | | | | |
| | | 2003–2017 | | -1.2 | -2.6 to 0.3 | | | | |
| Norway | National | 1978–2008 | -1.8* | | | 1978–2017 | 1.4* | | |
| | | 2008–2017 | 2.5* | | | 1978–2017 | | 1.4* | 0.8 to 2.2 |
| | | 1978–2017 | | -0.8* | -1.1 to -0.5 | 2003–2017 | | 0.9 | -1.9 to 4.0 |
| | | 2003–2017 | | 1.4* | 0.1 to 2.7 | | | | |
| Philippines | National | 1983–2017 | -2.1* | | | 1983–1985 | 61.2 | | |
| | | 1983–2017 | | -2.1* | -2.4 to -1.8 | 1985–2017 | -4.0* | | |
| | | 2003–2017 | | -1.9* | -3.1 to -0.7 | 1983–2017 | | -1.0 | -4.3 to 1.8 |
| | | | | | | 2003–2017 | | -3.8 | -7.6 to 0.3 |
| Republic of Korea | National | 1993–2006 | -5.2* | | | 1993–2017 | -2.4* | | |
| | | 2006–2017 | -2.7* | | | 1993–2017 | | -2.4* | -3.6 to -0.8 |
| | | 1993–2017 | | -4.1* | -4.5 to -3.6 | 2003–2017 | | -2.1 | -4.3 to 0.3 |
| | | 2003–2017 | | -3.3* | -3.7 to -2.7 | | | | |
| Spain | Tarragona, Granada, Murcia, Navarra, | 1986–2017 | -0.7* | | | 1986–2017 | -0.3 | | |

| | | | | | | | | | |
|---|---|---|---|---|---|---|---|---|---|
| | Basque Country, Girona, Canary Islands, La Rioja | 1986–2017 | | -0.7* | -1.0 to -0.3 | 1986–2017 | | -0.3 | -1.0 to 0.6 |
| | | 2003–2017 | | -1.1* | -2.1 to -0.03 | 2003–2017 | | -1.3 | -3.4 to 0.8 |
| Thailand | Chiang Mai, Khon Kaen, Songkhla, Lampang | 1993–2000 | 0.3 | | | 1993–2008 | -6.1 | | |
| | | 2000–2017 | -4.7* | | | 2008–2017 | 1.0 | | |
| | | 1993–2017 | | -3.3* | -3.9 to -2.5 | 1993–2017 | | -3.5* | -5.4 to -0.01 |
| | | 2003–2017 | | -4.3* | -5.4 to -3.3 | 2003–2017 | | 1.3* | 0.7 to 2.1 |
| The Netherlands | National | 1989–1998 | -1.3 | | | 1989–2017 | 0.8* | | |
| | | 1998–2001 | -5.8 | | | 1989–2017 | | 0.8* | 0.3 to 1.4 |
| | | 2001–2017 | 1.5* | | | 2003–2017 | | 1.3* | 0.7 to 2.1 |
| | | 1989–2017 | | -0.2 | -0.5 to 0.1 | | | | |
| | | 2003–2017 | | 1.5* | 0.8 to 2.1 | | | | |
| Türkiye | Izmir, Antalya | 1998–2017 | 0.2 | | | 1998–2017 | -3.3 | | |
| | | 1998–2017 | | 0.2 | -0.5 to 0.9 | 1998–2017 | | -3.3 | -7.4 to 1.1 |
| | | 2003–2017 | | 0.01 | -0.8 to 0.9 | 2003–2017 | | -14.8* | -4.4 to -20.6 |
| UK | England, Scotland, Northern Ireland, Wales | 1978–1987 | 1.7* | | | 1978–1989 | -0.4 | | |
| | | 1987–2000 | -4.7* | | | 1989–2017 | 1.5* | | |
| | | 2000–2017 | 0.9* | | | 1978–2017 | | 0.9* | 0.6 to 1.3 |
| | | 1978–2017 | | -0.8* | -1.0 to -0.6 | 2003–2017 | | 2.4* | 1.2 to 3.5 |
| | | 2003–2017 | | 1.0* | 0.4 to 1.7 | | | | |
| USA | California, Los Angeles County; USA, SEER (9 registries); USA, NPCR (45 States and Washington DC) | 1978–1996 | -0.8* | | | 1978–2017 | -0.3 | | |
| | | 1996–2003 | -3.3* | | | 1978–2017 | | -0.3 | -0.5 to 0.1 |
| | | 2003–2013 | -1.2* | | | 2003–2017 | | -0.1 | -0.9 to 0.7 |
| | | 2013–2017 | 0.5* | | | | | | |
| | | 1978–2017 | | -1.2* | -1.3 to -1.1 | | | | |
| | | 2003–2017 | | -0.7* | -1.1 to -0.4 | | | | |

APC, annual percentage change; AAPC, Average annual percentage change

* Statistically significant (P<0.05)

**Supplementary Table S4: International variations in average annual percentage change (AAPC) of HPV-related cancer incidence rates by site and sex (Continued)**

| Countries | Registries | Period | Head and neck, females | | | Period | Head and neck, males | | |
|---|---|---|---|---|---|---|---|---|---|
| | | | APC | AAPC(%) | 95%CI | | APC | AAPC(%) | 95%CI |
| Australia | New South Wales & Australian Capital Territory, Queensland, South Tasmania, Victoria, Western, Northern Territory | 1983–1999 | 0.6 | | | 1983–2004 | -1.2* | | |
| | | 1999–2002 | -4.7 | | | 2004–2017 | 1.4* | | |
| | | 2002–2017 | 1.7* | | | 1983–2017 | | -0.2* | -0.3 to -0.1 |
| | | 1983–2017 | | 0.6* | 0.3 to 0.9 | 2003–2017 | | 1.2* | 1.0 to 1.5 |
| | | 2003–2017 | | 1.2* | 0.2 to 3 | | | | |
| Austria | National | 1998–2008 | 2.7* | | | 1998–2017 | -0.7* | | |
| | | 2008–2017 | -0.9 | | | 1998–2017 | | -0.7* | -1.1 to -0.3 |
| | | 1998–2017 | | 1.0* | 0.01 to 2.2 | 2003–2017 | | -0.6 | -1.2 to 0.1 |
| | | 2003–2017 | | 1.3* | 0.4 to 2.7 | | | | |
| Belarus | National | 1983–1989 | 6.3* | | | 1983–1989 | 6.1* | | |
| | | 1989–2003 | -1.1 | | | 1989–1997 | 2.3 | | |
| | | 2003–2017 | 6.1* | | | 1997–2002 | -2.4 | | |
| | | 1983–2017 | | 3.1* | 2.3 to 4.3 | 2002–2017 | 1.8* | | |
| | | 2003–2017 | | 6.2* | 4.7 to 8.0 | 1983–2017 | | 2.0* | 1.8 to 2.4 |
| | | | | | | 2003–2017 | | 1.8* | 1.4 to 2.3 |
| Canada | Excl Nova Scotia, Northwest Territories, Nunavut, Quebec and Yukon | 1978–2012 | -0.5* | | | 1978–1986 | 0.8 | | |
| | | 2012–2017 | 3.1* | | | 1986–2003 | -1.7* | | |
| | | 1978–2017 | | -0.04 | -0.4 to 0.2 | 2003–2017 | 1.3* | | |
| | | 2003–2017 | | 0.9* | 0.2 to 1.8 | 1978–2017 | | -0.1 | -0.2 to 0.1 |
| | | | | | | 2003–2017 | | 1.4* | 1.0 to 1.8 |
| China | Beijing City, Qidong City, Shanghai City, Jiashan County, Wuhan City, Zhongshan City, Nangang District (Harbin City), Yanting County | 1998–2017 | -0.3 | | | 1998–2003 | -10.1* | | |
| | | 1998–2017 | | -0.3 | -1.2 to 0.8 | 2003–2017 | 0.5 | | |
| | | 2003–2017 | | 0.7 | -1.2 to 2.2 | 1998–2017 | | -2.4 | -3.6 to 0.03 |
| | | | | | | 2003–2017 | | 1.3* | 0.9 to 1.9 |
| Costa Rica | National | 1982–1999 | -3.7* | | | 1982–2016 | -1.4* | | |
| | | 1999–2016 | 0.8 | | | 1982–2016 | | -1.4* | -1.9 to -0.8 |
| | | 1982–2016 | | -1.5* | -2.6 to -0.1 | 2003–2016 | | -0.5 | -1.7 to 1.1 |

| | | | | | | | | | |
|---|---|---|---|---|---|---|---|---|---|
| | | 2003–2016 | | -0.1 | -2.7 to 2.8 | | | | |
| Croatia | National | 1988–1999 | 4.8* | | | 1988–2000 | 0.6 | | |
| | | 1999–2017 | -0.2 | | | 2000–2017 | -2.5* | | |
| | | 1988–2017 | | 1.7* | 0.3 to 3.1 | 1988–2017 | | -1.2* | -1.7 to -0.8 |
| | | 2003–2017 | | 0.1 | -1.4 to 1.6 | 2003–2017 | | -2.4* | -3.0 to -1.8 |
| Czech Republic | National | 1983–2017 | 3.9* | | | 1983–1994 | 1.6* | | |
| | | 1983–2017 | | 3.9* | 3.7 to 4.4 | 1994–2017 | 0.03 | | |
| | | 2003–2017 | | 4.6* | 3.6 to 5.9 | 1983–2017 | | 0.5 | 0.4 to 0.8 |
| | | | | | | 2003–2017 | | -0.1 | -0.5 to 0.2 |
| Denmark | National | 1978–2013 | 1.8* | | | 1978–1994 | 2.2* | | |
| | | 2013–2017 | -1.3 | | | 1994–2017 | 0.9* | | |
| | | 1978–2017 | | 1.5* | 1.2 to 2.0 | 1978–2017 | | 1.4 | 1.2 to 1.7 |
| | | 2003–2017 | | 1.1* | 0.2 to 2.3 | 2003–2017 | | 0.8 | 0.2 to 1.8 |
| France | Martinique, Bas-Rhin, Calvados, Doubs, Haut-Rhin, Isère, Somme, Hérault, Loire-Atlantique, Manche, Vendée | 1979–2017 | 1.6* | | | 1979–1985 | 1.7 | | |
| | | 1979–2017 | | 1.6* | 1.4 to 2.0 | 1985–2017 | -2.8* | | |
| | | 2003–2017 | | 1.8* | 1.1 to 2.6 | 1979–2017 | | -2.1* | -2.3 to -1.8 |
| | | | | | | 2003–2017 | | -2.3* | -2.7 to -1.9 |
| Germany | Hamburg, Bremen, Schleswig-Holstein, Saarland | 1998–2017 | 0.3 | | | 1998–2017 | -1.8* | | |
| | | 1998–2017 | | 0.3 | -0.4 to 1.2 | 1998–2017 | | -1.8* | -2.0 to -1.6 |
| | | 2003–2017 | | -0.7 | -1.9 to 1.1 | 2003–2017 | | -1.8* | -2.1 to -1.4 |
| India | Mumbai, Chennai, Barshi, Dindigul Ambilikkai | 1978–2006 | -1.9* | | | 1978–1983 | -4.7* | | |
| | | 2006–2017 | 0.01 | | | 1983–2007 | -1.0 | | |
| | | 1978–2017 | | -1.3* | -1.6 to -1.1 | 2007–2017 | 1.8* | | |
| | | 2003–2017 | | -0.1 | -0.7 to 0.5 | 1978–2017 | | -0.8* | -1.1 to -0.3 |
| | | – | | | | 2003–2017 | | 1.4* | 0.8 to 2.1 |
| Ireland | National | 1994–2017 | 2.6* | | | 1994–1997 | -8.0 | | |
| | | 1994–2017 | | 2.6* | 1.8 to 3.5 | 1997–2017 | 1.7 | | |
| | | 2003–2017 | | 2.5* | 0.8 to 4.6 | 1994–2017 | | 0.4 | -0.3 to 1.5 |
| | | | | | | 2003–2017 | | 2.0* | 0.7 to 3.4 |
| Israel | National | 1978–2014 | 1.0* | | | 1978–1998 | 0.6 | | |
| | | 2014–2017 | -8.1 | | | 1998–2017 | -1.1* | | |

| | | | | | | | | | |
|---|---|---|---|---|---|---|---|---|---|
| | | 1978–2017 | | 0.2 | -0.4 to 1.3 | 1978–2017 | | -0.2 | -0.5 to 0.1 |
| | | 2003–2017 | | -1.1 | -2.9 to 1.1 | 2003–2017 | | -1.1* | -2.0 to -0.1 |
| Japan | Miyagi Prefecture, Osaka | 1978–2003 | 0.8* | | | 1978–2004 | 0.2 | | |
| | | 2003–2017 | 4.4* | | | 2004–2017 | 2.1* | | |
| | | 1978–2017 | | 2.1* | 1.7 to 2.4 | 1978–2017 | | 0.9* | 0.5 to 1.2 |
| | | 2003–2017 | | 4.3* | 3.2 to 5.5 | 2003–2017 | | 2.1* | 1.3 to 2.9 |
| Lithuania | National | 1988–2008 | 1.0 | | | 1988–1994 | 3.2* | | |
| | | 2008–2017 | 7.9* | | | 1994–2017 | -0.2 | | |
| | | 1988–2017 | | 3.1* | 1.9 to 4.3 | 1988–2017 | | 0.5* | 0.1 to 1.0 |
| | | 2003–2017 | | 6.0* | 3.0 to 9.5 | 2003–2017 | | 0.03 | -1.0 to 0.8 |
| New Zealand | National | 1983–2000 | -1.4* | | | 1983–2002 | -1.3* | | |
| | | 2000–2017 | 2.6* | | | 2002–2017 | 1.9* | | |
| | | 1983–2017 | | 0.6* | 0.2 to 1.0 | 1983–2017 | | 0.1 | -0.2 to 0.4 |
| | | 2003–2017 | | 2.6* | 1.6 to 3.8 | 2003–2017 | | 1.9* | 0.9 to 3.1 |
| Norway | National | 1978–2017 | 1.8* | | | 1978–1984 | 4.1* | | |
| | | 1978–2017 | | 1.8* | 1.4 to 2.4 | 1984–2017 | 0.3 | | |
| | | 2003–2017 | | 2.1 | -0.4 to 4.9 | 1978–2017 | | 0.9* | 0.4 to 1.4 |
| | | | | | | 2003–2017 | | 0.6 | -0.2 to 1.6 |
| Philippines | National | 1983–1996 | -2.4 | | | 1983–1995 | 0.7 | | |
| | | 1996–2010 | -9.1* | | | 1995–2017 | -4.1* | | |
| | | 2010–2017 | 1.8 | | | 1983–2017 | | -2.4* | -3 to -1.7 |
| | | 1983–2017 | | -4.4* | -5.6 to -3.3 | 2003–2017 | | -4.2* | -6.0 to -2.4 |
| | | 2003–2017 | | -4.3* | -8.3 to -0.3 | | | | |
| Republic of Korea | National | 1993–2007 | -2.1 | | | 1978–1984 | 4.1 | | |
| | | 2007–2017 | 2.7* | | | 1984–2017 | 0.3 | | |
| | | 1993–2017 | | -0.2 | -1.8 to 1.3 | 1978–2017 | | 0.9* | 0.4 to 1.4 |
| | | 2003–2017 | | 1.9* | 0.7 to 3.3 | 2003–2017 | | -1.0* | -1.5 to -0.7 |
| Spain | Tarragona, Granada, Murcia, Navarra, Basque Country, Girona, Canary Islands, La Rioja | 1986–2017 | 2.8* | | | 1986–1994 | 0.4 | | |
| | | 1986–2017 | | 2.8* | 2.3 to 3.5 | 1994–2017 | -2.9* | | |
| | | 2003–2017 | | 2.6* | 1.1 to 4.2 | 1986–2017 | | -2.0* | -2.2 to -1.8 |
| | | | | | | 2003–2017 | | -3.0* | -3.4 to -2.3 |

| | | | | | | | | | |
|---|---|---|---|---|---|---|---|---|---|
| Thailand | Chiang Mai, Khon Kaen, Songkhla, Lampang | 1993–2001 | 0.3 | | | 1993–2001 | -0.5 | | |
| | | 2001–2004 | -24.1* | | | 2001–2004 | -21.8* | | |
| | | 2004–2017 | -1.6 | | | 2004–2017 | 1.4* | | |
| | | 1993–2017 | | -4.2* | -5.1 to -2.6 | 1993–2017 | | -2.5* | -3.2 to -1.1 |
| | | 2003–2017 | | -2.1 | -4.5 to 0.5 | 2003–2017 | | 1.0 | -0.2 to 2.3 |
| The Netherlands | National | 1989–1996 | 3.9* | | | 1989–2017 | -0.7* | | |
| | | 1996–2014 | 1.0* | | | 1989–2017 | | -0.7* | -0.9 to -0.5 |
| | | 2014–2017 | -3.7 | | | 2003–2017 | | -1.2* | -1.8 to -0.6 |
| | | 1989–2017 | | 1.2* | 0.8 to 1.8 | | | | |
| | | 2003–2017 | | 0.1 | -0.5 to 0.6 | | | | |
| Türkiye | Izmir, Antalya | 1998–2004 | 5.9* | | | 1998–2002 | 5.4* | | |
| | | 2004–2007 | -11.1* | | | 2002–2017 | -3.1* | | |
| | | 2007–2017 | 0.8 | | | 1998–2017 | | -1.3* | -1.9 to -0.4 |
| | | 1998–2017 | | 0.4 | -0.3 to 1.7 | 2003–2017 | | -3.1* | -3.9 to -2.3 |
| | | 2003–2017 | | -2.2* | -3.7 to -0.4 | | | | |
| UK | England, Scotland, Northern Ireland, Wales | 1978–2005 | 2.0* | | | 1978–2005 | 1.1* | | |
| | | 2005–2012 | 4.1* | | | 2005–2013 | 2.9* | | |
| | | 2012–2017 | 1.1 | | | 2013–2017 | 0.7 | | |
| | | 1978–2017 | | 2.3* | 2.2 to 2.4 | 1978–2017 | | 1.4* | 1.3 to 1.6 |
| | | 2003–2017 | | 2.8* | 2.4 to 3.4 | 2003–2017 | | 2.2* | 2.0 to 2.4 |
| USA | California, Los Angeles County; USA, SEER (9 registries); USA, NPCR (45 States and Washington DC) | 1978–2001 | -1.5* | | | 1978–1983 | 0.8 | | |
| | | 2001–2017 | 0.01 | | | 1983–1994 | -1.8 | | |
| | | 1978–2017 | | -0.9* | -1.0 to -0.8 | 1994–2017 | 0.2* | | |
| | | 2003–2017 | | -0.05 | -0.2 to 0.1 | 1978–2017 | | -0.3* | -0.4 to -0.1 |
| | | | | | | 2003–2017 | | 0.2* | 0.03 to 0.4 |

APC, annual percentage change; AAPC, Average annual percentage change

* Statistically significant (P<0.05)
